# Biochar-based organic fertilizers: Influence on yield and concentration of antioxidants in the stigma of saffron and rhizosphere bacterial diversity of slightly saline and non-saline soils

**DOI:** 10.1016/j.sjbs.2023.103922

**Published:** 2024-01-05

**Authors:** Shagufta Qasim, Shamim Gul, Abdul Hanan Buriro, Fahad Shafiq, Tariq Ismail

**Affiliations:** a Department of Botany, University of Balochistan, Pakistan; b Department of Natural Resource Sciences, McGill University, QC, Canada; c Shaheed Zulfiqar Ali Bhutto Agricultural College, Larkana, a constituent college of Sindh Agriculture University, Tando Jam, Sindg, Pakistan; d Institute of Molecular Biology and Biotechnology, The University of Lahore, Pakistan; e Department of Botany, Government College University, Lahore, Punjab, Pakistan

**Keywords:** *Crocus sativus*, Wood-derived biochar, Flavonoids, Carotenoids, Total polyphenolics, Co-composted biochar, 16s rRNA metagenomics

## Abstract

Being the most expensive spice, saffron has great economic importance. This crop grows well in cold arid deserts. Salinity is one of the important limiting factors for the cultivation of this crop. However, the use of composted manured and co-composted biochar and fertilizers can play a role in attenuating the salinity stress on this crop. In this two-year field study, manures from three sources: sheep and goat (*SG*), cow and buffalo (*FYM*), and poultry (*PM*) farms, and their co-compost with slow-pyrolyzed wood-derived biochar (B) were used for saffron cultivation in slightly saline (electrical conductivity 1.95 dS m^−1^) and non-saline soils. Yield and concentration of antioxidants of stigma and bacterial diversity in the rhizosphere of this crop, under salinity and non-salinity conditions, were evaluated. Results revealed that in non-saline soil of first-year crops, all fertilizers decreased the yield of stigma than control by 15–49 % (P ≤ 0.05) but increased the concentration of carotenoids and total polyphenolics (P ≤ 0.05). In saline soil, no difference in yield was observed between treatments for the first-year crop; however, for the second-year crop, as compared to control, *PM* and *FYM* significantly increased yield by 41 % and 44 % respectively, whereas *FYM* also increased the concentration of total polyphenolics (P ≤ 0.05). The *FYM* fertilizer was found suitable for the yield and quality of saffron stigma for second-year crops in both soils (non-saline and saline). The observed OTUs, Chao1, Fischer, and ACE indexes based on 16 s rRNA metagenomic analysis revealed 2–4 times greater bacterial diversity in the rhizosphere soil of PM-B and SG-B treatments than in the control. Furthermore, 347 bacterial species were found in PM-B- or SG-B-amended soils absent in control treatments.

## 1 Introduction

The perennial medicinal herb *Crocus sativus* L. (Saffron) belongs to the Iridaceae family. Its stigma is used in pharmaceuticals, confectionery, cosmetics, and textile dye industries (Kothari et al., 2021). It is a triploid plant, and its propagation is carried out through corms (Bayat et al., 2016, Caser et al., 2020). Being the most expensive crop, this spice is also called red gold. The price of saffron stigmas varies from 1500 to 2200 Euro per kilogram (Mykhailenko et al., 2020). This medicinal plant is expensive because each corm can produce one to four flowers; each flower has only three long orange-colour stigmas, 200 to 300 dry stigmas weight 1 g; therefore, it takes approximately 200,000 flowers to obtain I kg dry stigma (Mykhailenko et al., 2020, Gheshm and Brown, 2021) Iran, Italy, Spain, India and Morocco are the major producers of this crop (Kothari et al., 2021). The quality of saffron stigma depends on its aroma and the concentration of antioxidants such as total polyphenolics, carotenoids, and flavonoids (Gresta et al., 2009). Due to high profitability and demand in pharmaceuticals, confectionary, and textile dye industries, cultivation of this crop is gathering momentum worldwide (Mohammadi and Reed, 2020).

Climatic conditions, altitude, and soil pedology, especially texture, are important factors that influence the production of saffron (Rahimi et al., 2017, Siracusa et al., 2020, Cardone et al., 2020). A cold, dry Mediterranean climate is suitable for the growth of this medicinal crop and requires less irrigation (Gresta et al., 2008, Shokrpour, 2019, Salas et al., 2020). Similarly, an altitude of 1300 m to 2300 m is suitable for its cultivation (Fallahi et al., 2021, Zamani et al., 2022). Generally, this crop grows well in soils with high sand fraction (sandy, sandy loam, sandy silt loam soils) (Cardone et al., 2020, Shahandeh, 2020). For instance, according to the International Trade Centre report (International Trade Centre, Afghanistan’s National Export Strategy 2018–2022; Saffron Sector: Geneva, Switzerland, 2018) in Afghanistan, saffron grows best in calcium-rich soil and has a sandy-loam texture. Likewise, in two years field study in Potenza, Southern Italy which has a Mediterranean climate, Cardone et al. (2020) found significantly higher growth of saffron in soils with 29–52 % sand and 20–40 % clay than the soils with 90.6 % sand.

Balochistan, with an area of approximately 34.7 million hectares, occupies ∼ 44 % of the total land area of Pakistan. Approximately, 50 % of the land area of this province falls in the Mediterranean climate and lies between 1000 m and 1300 m elevation (Ahmad et al., 2012, Ahmed et al., 2014, Saeed et al., 2017). The climate, pedology (as most of the soils of these cold deserts have high sand fraction) and altitude of these regions are well-suited for the growth of saffron. Right now, saffron is cultivated on test trial bases in agricultural farms of Mastung city and in Balochistan Agricultural Research and Development Centre, Quetta, Balochistan. On a commercial basis, this crop is still not cultivated in this province. One of the reasons is that many parts of these cold dry regions of Balochistan have saline soils (Syed et al., 2020), which is one of the important limiting factors for the growth of saffron. A saline soil is defined as one, which has an electrical conductivity of 2 dS m^−1^ and above (Corwin and Scudiero, 2019, Hopm, xxxx). A significant decline in the yield of corn was observed when electrical conductivity was increased to 1 dS m^−1^ in sandy loam and to 2 dS m^−1^ in clay loam soil (Beltrão and Asher, 1997). This indicates that crops are more vulnerable to salt stress when they are grown in soils, which have high sand fraction and as stated above, generally, soils of cold deserts of Balochistan have high sand fraction. However, the salinity factor can be controlled with well-suited agricultural management practices such as the use of biochar-based organic fertilizers (Song et al., 2022, Qian et al., 2023).

Biochar is a pyrogenous black biomass. It is produced from the partial burning of biomass (such as wood, manures, crop stover, animal bones, and algae) under oxygen-deficient conditions (Gul et al., 2015). Biochar is considered a soil conditioner that improves crop yield and physico-chemical and biological properties of saline soils of dry regions (Feng et al., 2020, Mao et al., 2022). However, for soils that have a high sand fraction, a positive influence of biochar has been observed when this bioresource is applied in the soil as a mixture with other synthetic or organic fertilizers (Gul and Whalen, 2016, Achakzai et al., 2023). Likewise, biochar amendment in agricultural lands as a mixture or co-compost with organic wastes such as manures tends to improve crop growth and health of saline soils of dry regions (Wu et al., 2022, Al-Omran et al., 2023, El-Wahed et al., 2023).

Microorganisms are important soil health indicators. Microorganisms in the rhizosphere of crop roots play an important positive role in nutrient cycling, plant defence mechanism and ultimately plant health. Root microbiome also plays an important role in protecting plants from various stresses such as salinity and drought (Caddell et al., 2019). Biochar-based organic amendments tend to enhance bacterial diversity, microbial activities in the rhizosphere and crop growth under saline stress conditions (Lu et al., 2015). For instance, Lu et al. (2015) reported a significant increase in the bacterial diversity in the rhizosphere soil of maize and its yield in saline soil, in response to the amendment of biochar, which was co-composted with cattle manure. For saffron, Ghanbari et al. (2019) and Ghanbari and Khajoei-Nejad (2022) reported a significant influence of fertilizers from cattle manure compost and compost + biochar mixture on soil quality and the stigma yield in sandy loam soil under field conditions. Moreover, Ghanbari et al. (2019) also found an increase in the quality of saffron stigma in terms of a high concentration of total polyphenolics in the stigma of saffron in response to the amendment of biochar manure compost mixture in soil under field conditions. However, no empirical evidence exists regarding the influence of biochar mixture or its co-compost with manures from different sources (poultry, cow and buffalo, and sheep and goat farms) on the yield and quality of saffron and bacterial diversity in the rhizosphere of this crop under saline soil conditions.

In Balochistan, wood-derived biochar from *Acacia nilotica* L. is available on commercial bases for Bar-BQ purposes. As compared to synthetic fertilizer, small broken leftover pieces of this biochar are approximately three times less expensive (Hameeda et al., 2019). Furthermore, in this province, manures from poultry, sheep and goat, and cow and buffalo farms are available at low prices. These biowastes (manures) and broken leftover pieces of biochar can be utilized to produce co-composted biochar to promote agriculture in saline soils of this region besides reducing pollution caused by their improper disposal. Taking into consideration the use of slightly saline sandy loam soil (electrical conductivity 1.95 dS m^−1^), this two-year field-based study aims to investigate the influence of manures from the farms of 1) cow and buffalo, 2) sheep and goat and 3) poultry; as well as, their co-compost with wood-derived biochar on the yield and concentration of antioxidants of stigma of saffron and 4) diversity of bacteria in the rhizosphere of saffron corms. We hypothesized that 1) co-composted biochars increase the yield and improve the quality of saffron stigma and 2) increase the bacterial diversity of rhizosphere soil as compared to control treatments. For bacterial diversity assessment, we considered co-composted biochar with poultry manure and co-composted biochar with manure from sheep and goat farms. Due to fund limitation and since poultry manure is the most inexpensive fertilizer; whereas manure from sheep and goat farms is the most expensive and preferred for agriculture, we selected these two treatments under consideration for metagenome analysis. This is the first study that evaluated the influence of three commonly available manures and their co-compost with biochar on saffron stigma yield and rhizosphere bacterial community under salinity stress conditions.

## 2 Materials and methods

### 2.1 Study site

**Fig. 1:**
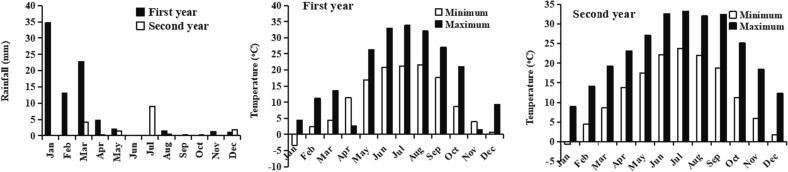
Total rainfall and average minimum (night-time) and average maximum (daytime) temperature of Quetta city in 2020 and 2021.

This experiment was performed in the research field of Balochistan Agriculture Research and Development Centre (BARDC) Quetta, 30°11ʹ38″ N, 66°57ʹ19″ E. This research was conducted for two growing seasons; mid of August (when leaf emergence started) from 2020 to mid-April 2021 and mid of August 2021 to mid of April 2022. With cold rainy winters and dry summers, the climate of the study site is Mediterranean (Fig. 1). This region also receives snowfall in winter.

### 2.2 Biochar, manures, their co-composting, and chemical analysis

In this study, biochar produced from *Acacia nilotica* L. wood was used. This slow-pyrolyzed biomass was purchased from the timber market of Quetta, Balochistan, Pakistan. The method of production of this biochar is given in Achakzai et al. (2023). The manures used as fertilizers and for the composting process were obtained from sheep/goat (*SG*), cow/buffalo (*FYM*) and Poultry (*PM*) farms in Quetta and the outskirts of this city. These fertilizers were air-dried and used for direct amendment in soil and for co-composting with crushed biochar (particle size ≤ 4 mm). These organic fertilizers were mixed separately with biochar at a 1:1 ratio in 200 L large plastic drums filled with tap water. These mixtures were left for six months in the open air for aerobic decomposition from January to June. Every week, these mixtures were thoroughly mixed with wooden sticks during the entire composting period.

Approximately 5 g of Air-dried composted manures and their co-compost with biochar were further oven-dried at 60 °C for 24 h and analyzed for nitrogen, phosphorus, potassium, and sodium following the protocol described in Estefan et al. (2013). The pH of these fertilizers was measured as follows; a mixture of fertilizer and deionized water was made at a 1:10 fertilizer: water (w: v) ratio and checked for pH after 18 h of incubation at room temperature (Ghori et al., 2019). Ash of fertilizers was analyzed by weight loss-on-ignition, gravimetric method (Estefan et al., 2013).

### 2.3 Experimental design and treatments

In this experiment, saline soil was transported from Mulkiyar, Pishin, Balochistan from an agricultural farm; however, for non-saline soil treatment, the native soil of the study site was used. Saline soil was spread over the soil surface as an approximately 10 cm thick layer. The properties of soil are given in Table 1. All chemical properties of soils mentioned in Table 1 are based on analyses made following the protocol of Estefan et al. (2013). The electrical conductivity of saline soil was approximately 2 dS m^−1^ (1.95 dS m^−1^), and the concentration of total dissolved salts was 812 ppm. This type of soil is considered as slightly acidic. A total of 21 plots of 1x1 m size were established in saline and 21 plots of the same size were established in non-saline soil. There was a buffer of ∼ 0.25 m between each plot and there was no outlet for parallel flow of water between plots (Supplementary Fig. 1). The experiment was performed in a randomized complete block design (RCBD), in which each treatment was replicated three times. The treatments were as follows; (1) Control (fertilizers were not applied), (2) small ruminant (sheep and goats) manure (*SG*), (3) farmyard manure (manure from cow and buffalo) (*FYM*), (4) poultry manure (*PM*), (5) co-composted biochar with *SG* (*SG-B*), (6) co-composted biochar with *FYM* (*FYM-B*), (7) co-composted biochar with *PM* (*PM-B*). The literature review by Gul and Whalen (2016) suggested that biochar-based organic amendments tend to have a positive influence on the yield of crops when they are applied at rates higher than 10 t ha^−1^. The *meta*-analysis of Wang et al. (2019) suggested that the application rate of co-composted biochars less than 20 t ha^−1^ and more than 30 t ha^−1^ improved yield of crops. Considering these studies, for the first-year crop, 15 t ha^−1^ of these organic fertilizers were added in plots in RCBD design. Because the application rate of fertilizers as 15 t ha^−1^ did not increase the yield of the stigma of the first-year crop, we increased the application rate of fertilizers to 50 t ha^−1^ for the second-year crop. The same fertilizer was added in each plot as was amended for first-year crops. Fertilizers for both cropping years were added in mid-August. These amendments were mixed at 1–3 cm depth of soil in each plot (see Table 2).

**Table 1:** Properties of non-saline and saline soil used in this study.

Physico-chemical properties	Non-saline soil*	Saline soil
pH	7.9	7.23
Electrical conductivity (dS m^−1^)	0.25	1.95
Total dissolve salts (ppm)	–	812
Organic matter (g kg^−1^)	9.9	30.9
Silt (g kg^−1^)	500	475
Clay (g kg^−1^)	50	50

* Soil properties of non-saline soil is taken from Ghani et al., 2022.

**Table 2:** Chemical properties of organic fertilizers used in this study.

Organic fertilizers	Nitrogen (mg/g)	Phosphorus (mg/g)	Potassium (mg/g)	Sodium (mg/g)	pH	Ash (mg/g)
Composted sheep/goat (*SG*) manure	5.16	2.18	178.8	156.4	8.40	285
Composted farmyard manure (*FYM*)	6.94	3.83	142.2	173.9	7.88	849
Composted poultry manure (*PM*)	14.8	7.82	133.0	114.2	7.96	789
*SG* + biochar co-compost	5.10	3.40	134.0	137.5	8.09	176
*FYM* + biochar co-compost	1.36	1.36	127.9	106.9	7.81	218
*PM* + biochar co-compost	3.09	1.90	88.30	137.5	8.37	753

### 2.4 Saffron cultivation

Saffron corms were obtained from BARDC (Balochistan Agricultural Research and Development Centre) Quetta. The corms were obtained from 2 − 3-year-old crops with a weight of 18–20 g approximately and a width of 3–4 cm, having no cuts or wounds. They were treated with fungicide for almost 30 min before sowing to protect them from diseases/infections. Corms were planted in the soil at a depth of ∼ 5–6 cm. Inside each plot, four rows were made, and within each row, four corms with 25 cm were sown. A total of 16 corms per replication and 48 corms per treatment were planted. After the sowing of corms, plots were irrigated immediately.

### 2.5 Harvest of stigmas

For the first growing season, germination of corms started on October 18, 2020, and flowering started on October 28, 2020. The total flowering period for the first growing season was about 19 days and ended on November 23, 2020. The emergence of leaves for the second growing season started on October 8, 2021, and flowering began on October 20, 2021, which lasted for 18 days and ended on November 12, 2021. Flowers were picked every day early in the morning, and fresh weight of saffron stigma was recorded immediately.

### 2.6 Harvest of corms

In April 2021 and April 2022, corms from three sowing points in each plot were harvested. The number of corms from each sowing point was recorded. The fresh weight of corms of the first-year crop was also recorded and divided into four weight classes: 0–5 g, >5–10 g, >10–15 g and > 15–20 g.

### 2.7 Estimation of antioxidants in stigmas

Air-dried stigmas were sent to the Institute of Molecular Biology and Biotechnology, The University of Lahore, Lahore, Pakistan for the estimation of carotenoids, total polyphenolics, and flavonoids. The total polyphenolics and flavonoids were estimated using the method of Bray and Thorpe (1995); and Pekal and Pyrzynska (2014) as described in Shafiq et al. (2019). The carotenoids were determined with the method of Kirk and Allen (1965).

### 2.8 Rhizosphere soil sample collection

In March 2021 (the second year of cropping), corms from three sowing spots per plot were dug and the corms were isolated. Corms were gently shaken and the soil that was adhered to corms was carefully collected in labelled Ziplock bags. The soil samples were stored at −20 °C before further processing for metagenomics analysis. Soil samples were sent to ABO Laboratories, Islamabad, Pakistan within one month of collection for metagenomics analysis.

### 2.9 Metagenomic analysis of saffron rhizosphere soil samples

The rhizosphere soil samples collected from the corms of the second-year crop in March were subjected to DNA extraction with a DNA isolation kit (PowerLyzer® Power Soil®) according to the instructions of the manufacturer. The purity of DNA was assessed before its storage at −20 °C. DNA samples were stored before sequencing at −20 °C.

To perform the metagenomics sequencing of 16S rRNA, DNA samples were sent to Novogene, Beijing. V3-V4 variable region primers were used to make amplicons. For this purpose, the hypervariable V3-V4 region of the 16S rRNA gene was amplified with the pair of primers i.e., 341F:5′-CCTAYGGGRBGCASCAG-3′ and 806R:5′-GGACTACNNGGGTATCTAAT-3′. The Polymerase Chain Reactions (PCR) were performed with Phusion® High-Fidelity PCR Master Mix (New England Biolabs). The same volume of 1X-loading buffer, which contained SYBR green was mixed with PCR products, followed by electrophoresis using agarose gel. Thereafter, samples with 400–470 bp size were selected for further analysis. The Ion Plus Fragment Library kits were considered for generating libraries and were thereafter sequenced using the IonS5TMXL (Thermofisher, USA) platform (Novogene, Beijing, China).

The pre-processed single-end reads and 16S rRNA demultiplexed were obtained from Novogene, Beijing. These reads were analysed using Quantitative Insights into Microbial Ecology (QIIME ver.2.2020.6) software. The single-end FASTQ reads were imported in QIIME with the method of q2 manifest file import and quality filtered with the help of the q2-dada2 denoising method. The reads longer than 425 bp size were removed, whereas chimeric sequences were filtered. The QIIME tool was used to produce Amplicon Sequence Variants (ASV) frequency and their representative sequences tables. A q2-feature-classifier, machine learning method (classify-sklearn naive Bayes taxonomy classifier) was used for assigning the taxonomy to the ASV representative sequences using the method described in https://www.arb-silva.de/. The q2 taxa barplot method was used taxonomic composition of samples from phylum to species levels. For bacterial diversity analysis, the core-metrics-phylogenetic method, under the q2-diversity plugin in QIIME was used. For the estimation of alpha diversity (i.e., within sample diversity), the following diversity metrics were considered; Chao1 (observed taxonomic units (OTUs) richness), Shannon index (quantitative measure of the number of OTUs and their relative abundance (community richness) and Simpson metrics. For Beta diversity, which is the diversity between samples, distance metrics were measured including unweighted and weighted UniFrac (a qualitative measure of community dissimilarity based on phylogenetic relationships between the features).

### 2.10 Statistical analysis

The data of fresh yield of stigma, weight classes of corms, total number of corms, polyphenolics, carotenoids, and flavonoids were tested for normality before analysis of variance test. The data of fresh yield of stigma were subjected to two-way ANOVA under randomized complete block design, where the factors were soil type and fertilizer treatments. Because for rhizosphere soil collection, corms were collected from three spots from each plot, which might have affected yield, the factor of the year was not considered for the analysis of stigma yield. The data of various corm weight classes were subjected to two-way ANOVA under a randomised complete block design, where the factors were fertilizer treatments and soils (non-saline and saline). The data of a total number of corms were subjected to three-way ANOVA under randomised complete block design, where the factors were fertilizer treatments, soils, and year of cropping. The data of antioxidants were subjected to three-way ANOVA under a randomised complete block design where the factors were years of cultivation, soil type and fertilizer treatments. The differences between treatment means were measured using the least significance difference (LSD) test. The data of fresh yield of stigma, weight classes of corms, the total number of corms, polyphenolics, carotenoids, and flavonoids were analysed using Costate and Microsoft Excel. Alpha-diversity indexes were compared using a nonparametric Kruskal– Walli’s test. To visualize beta diversity relationships, principal coordinate analysis (based on unweighted and weighted UniFrac measures) was used. Permutational ANOVA (PERMANOVA) test was used for Beta diversity metrics analysis.

## 3 Results

### 3.1 Yield of stigma

The yield of stigma in non-saline soil was higher than in saline soil (P < 0.05). Soil type × fertilizer treatment interaction was non-significant (P < 0.05). In non-saline soil, as compared to the control treatment, amendment of all fertilizers significantly decreased the yield of fresh stigma by 15 – 49 % (P ≤ 0.05; Table 3). For first-year crops grown in non-saline soil, the yield of stigma was lower in *PM-B* treatment than in all other treatments (P ≤ 0.05; Table 3). The difference between treatments was non-significant for the first-year crop of saline soil and for the second-year crop of non-saline soil (P ≤ 0.05; Table 3). For the second-year crop of saline soil, amendments of *FYM* and *PM* fertilizers significantly increased yield than control by 44 and 41 % respectively (P ≤ 0.05; Table 3). No difference in fresh stigma yield was observed between saline versus non-saline soil for a given treatment was observed except SG fertilizer treatment. The yield of the first-year crop grown in *SG*-amended non-saline soil was significantly higher than the yield of the first-year crop grown in *SG*-amended saline soil by 32 % (P ≤ 0.05; Table 3).

**Table 3:** Mean (±standard deviation) fresh weight of stigmas (g/m^−2^(−|-)).

**Treatments**	**First year crop**		**Second year crop**	
	**Non-saline soil**	**Saline soil**	**Non-saline soil**	**Saline soil**
***Control***	1.04 ± 0.21**^a^**	0.76 ± 0.30	1.65 ± 0.25	0.73 ± 0.19**^b^**
***SG***	0.79 ± 0.08**^b^**	0.54 ± 0.06	1.18 ± 0.43	0.99 ± 0.30**^ab^**
***FYM***	0.70 ± 0.11**^b^**	0.63 ± 0.27	1.27 ± 0.25	1.30 ± 0.37**^a^**
***PM***	0.77 ± 0.13**^b^**	0.70 ± 0.16	1.32 ± 0.47	1.24 ± 0.33**^a^**
***SG-B***	0.89 ± 0.36**^b^**	0.44 ± 0.11	1.49 ± 0.56	0.73 ± 0.24**^ab^**
***FYM-B***	0.74 ± 0.07**^b^**	0.64 ± 0.19	1.60 ± 1.10	1.13 ± 0.45**^ab^**
***PM-B***	0.42 ± 0.12**^c^**	0.65 ± 0.13	1.21 ± 0.64	0.96 ± 0.34**^ab^**

Within the column, values with different letters indicate a significant difference between treatments (P ≤ 0.05).

### 3.2 External morphology, various weight classes, and total number of saffron corms

**Fig. 2:**
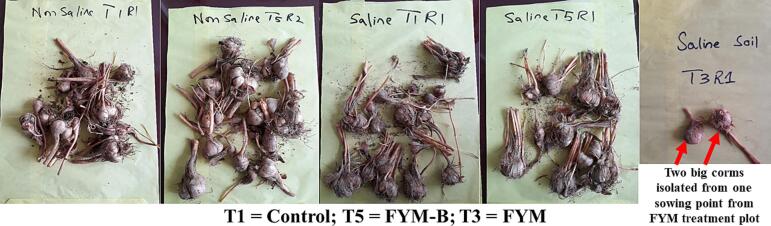
Corms collected from a single sowing point from various plots after second-year growth in April 2022. The surface of corms selected from non-saline soil is smooth, whereas corms collected from saline soil are covered with dead tissues.

Corms grown in non-saline soil had smooth surfaces, whereas corms grown in saline soil had dead covers (Fig. 2). Crop grown in non-saline soil, no difference between treatments was observed regarding the weight of corms of 0–5, >5–10 and > 10–15 g weight classes. However, corms of > 15–20 g weight class of control and *PM-B* treatments were significantly higher as compared to *FYM* treatment by 64 and 69 % respectively, as compared to *SG-B* treatment by 93 and 94 % respectively and as compared to PM treatment by 72 and 75 % respectively (P ≤ 0.05; Table 4).

**Table 4:** Mean (±standard deviation) number of corms of various weight classes and total number of corms collected from the spots where corms were first placed for sowing (to assess production of corms from mother corm).

**Treatments**	**Number of corms in various weight classes (1st year data only)**								**Total number of corms**		**Total number of corms**	
	Non-saline soil				Saline soil				Non-saline soil		Saline soil	
	≤5g	>5–10 g	>10–15 g	>15–20 g	≤5g	>5–10 g	>10–15 g	>15–20 g	1st yr.	2nd yr.	1st yr.	2nd yr.
***Control***	1.33 ± 1.2	2.88 ± 1.0	2.22 ± 1.4	1.55 ± 1.1^ab**^	0.77 ± 0.6^b^	1.77 ± 1.9	1.77 ± 1.3^ab^	0.33 ± 0.7^ab^	8.00 ± 2.18^a^	17.5 ± 4.6**^bA^**	4.66 ± 2.3^bc^	15.6 ± 5.5**^abA^**
***SG***	1.22 ± 0.9	1.88 ± 1.4	1.77 ± 1.2	0.88 ± 1.0**^abc^**	0.77 ± 0.6^b^	1.77 ± 1.2	1.11 ± 1.0^bc^	1.33 ± 1.8^a^	5.77 ± 2.7^ab^	18.6 ± 8.1**^abA^**	5.00 ± 2.9^abc^	15.3 ± 6.1**^bA^**
***FYM***	1.22 ± 0.6	2.55 ± 2.2	2.22 ± 1.7	0.55 ± 0.5**^c^**	1.22 ± 1.1^ab^	2 ± 1.5	1.44 ± 0.7^abc^	0.77 ± 1.1^ab^	6.55 ± 3.1^ab^	18.4 ± 6.2**^abA^**	5.44 ± 1.1^abc^	15.2 ± 5.3**^bA^**
***PM***	2.11 ± 2.4	1.88 ± 1.6	1.88 ± 1.1	0.44 ± 0.7**^c^**	1.77 ± 1.3^a^	1.55 ± 1.9	0.66 ± 1^bc^	0.22 ± 0.6^b^	6.33 ± 1.7^ab^	24.7 ± 12.3**^aA^**	4.22 ± 1.9^bc^	21.7 ± 13.2**^aA^**
***SG-B***	1.66 ± 1.0	2.44 ± 2.3	1.11 ± 1.1	0.11 ± 0.3**^c^**	0.77 ± 1.2^b^	2.44 ± 1.0	2.66 ± 2.5^a**^	1.11 ± 1.4^ab^	5.33 ± 2.1^b^	19 ± 5.1**^abA^**	7.00 ± 4.0^a^	13.8 ± 2.8**^bA^**
***FYM-B***	1.22 ± 1.2	2.22 ± 2.1	1.22 ± 1.4	0.66 ± 1.1**^bc^**	1.22 ± 0.8^ab^	2.88 ± 2.0	1.33 ± 1.1^bc^	0.77 ± 1.1^ab^	5.33 ± 1.8^b^	14.8 ± 3.0**^bA^**	6.22 ± 1.9^ab^	16.6 ± 5.3**^abA^**
***PM-B***	1.77 ± 1.1	1.33 ± 0.8	1.66 ± 1.3	1.77 ± 1.7^a**^	1.22 ± 1.1^ab^	1.44 ± 1.3	0.44 ± 0.8^c^	0.44 ± 0.5^ab^	6.55 ± 2.8^ab^	17 ± 7.0**^bA^**	3.55 ± 1.5^c^	17.5 ± 3.7**^abA^**

Within column, values with different lower-case letters are significantly different (P ≤ 0.05), ^**^ indicates significance difference between non-saline and saline soils for a given treatment (P ≤ 0.05); whereas uppercase letters show significance difference between first- and second-year crop (P ≤ 0.05) for a given soil type.

For the crop grown in saline soil, no difference between treatments was observed for the corm weight class of > 5–10 g. However, the corm weight class of 0–5 g was significantly higher for the crop that was grown under *PM* fertilizer treatment than control, *SG* and *SG-B* treatments, by approximately 56 % (P ≤ 0.05; Table 4). Similarly, corm weight class of > 10–15 g was significantly higher under *SG-B* fertilizer treatment than *SG*, *PM*, *FYM-B* and *PM-B* by 58, 75, 50 and 83 % respectively (P ≤ 0.05; Table 4). Likewise, the corm weight class of > 15–20 g was significantly higher under the treatment of *SG* than *PM-B* by 83 % (P ≤ 0.05; Table 4). The fertilizer treatments × soil interaction was significant only for corm weight class > 10–15 g (P < 0.05). The corm weight class of > 15–20 g was significantly higher under control and PM-B treatments in non-saline soil as compared to the corm weight class of > 15–20 g under control and PM-B treatments in saline soil (P < 0.05; Table 4).

The fertilizer treatment × soil interaction for the data of total number of corms was not significant; however, the fertilizer treatment × year interaction was significant (P < 0.05). The total number of corms was significantly higher for the second-year crop in both soils (non-saline and saline) than for the first-year crop (Table 4). For the crop grown in non-saline soil, the total number of corms of first-year crop under treatments of *SG-B* and *FYM-B* was 33 % significantly lower than control treatment (P ≤ 0.05; Table 4). For the second-year crop, the total number of corms under treatment of PM fertilizer was significantly higher than control, *FYM-B* and *PM-B* treatments by 29, 40 and 31 % respectively (P ≤ 0.05; Table 4).

For the crop grown in saline soil, for the first-year crop, the number of corms under *SG-B* fertilizer treatment was significantly higher than control, *PM*, and *PM-B* treatments by 33, 40 and 49 % respectively. For the second-year crop, the number of corms was significantly higher in *PM* treatment than in *SG*, *FYM* and *SG-B* treatments by 30, 30 and 36 % respectively (P ≤ 0.05; Table 4). The number of corms in the second-year crop was 2–4 times higher than the first-year crop for both soils (non-saline and saline); furthermore, there was no difference between soils (non-saline and saline) in this regard (P ≤ 0.05; Table 4).

### 3.3 Concentration of carotenoids, total polyphenolics, and flavonoids in stigmas

#### 3.3.1 Carotenoids

**Fig. 3:**
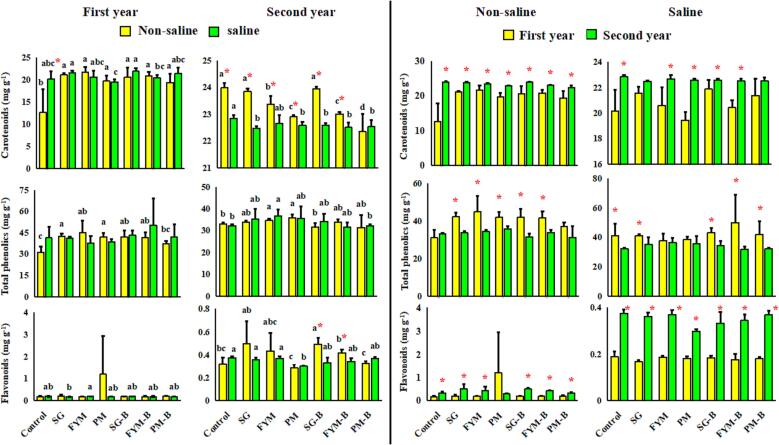
The concentration of carotenoids, flavonoids, and total polyphenolics in saffron stigma under the influence of various fertilizer treatments and salinity or non-salinity stress conditions. Values are mean ± SD. Bars with different letters show significant differences at P ≤ 0.05 between factors (control and different fertilizers) of a given treatment (saline or non-saline), whereas * indicates a significant difference at P ≤ 0.05 between treatments (saline versus non-saline and first versus second year of saffron cultivation) for a given factor (control or other fertilizer treatments).

The fertilizer treatments × soil interaction was non-significant for all three antioxidants. The fertilizer treatments × year interaction for all three antioxidants was significant (P < 0.05). The fertilizer treatments × soil × year interaction for all three antioxidants were non-significant. For the first-year crop grown in non-saline soil, as compared to the control treatment, all fertilizers increased the concentration of carotenoids in stigmas by 35–42 % (P ≤ 0.05; Fig. 3); however, for the second-year crop, except *SG* and *SG-B* treatments, all other fertilizers reduced the concentration of carotenoids by ∼ 3 to 7 % than control (P ≤ 0.05; Fig. 3). For the second-year crop, the concentration of carotenoids under *FYM-B* was lower by 1.5 % than the *FYM* treatment. Similarly, for the second-year crop, the concentration of carotenoids under *PM-B* treatment was lower by 2.4 % than in *PM* treatment (P ≤ 0.05; Fig. 3).

For the first-year crop grown in saline soil, the concentration of carotenoids in stigma was lower in response to *PM* and *FYM-B* treatments than *SG* and *SG-B* treatments by 5 and 11 % respectively (P ≤ 0.05; Fig. 3). For the second-year crop, all treatments accepted *FYM* (which caused no influence) and reduced the concentration of carotenoids by 1.1–1.6 % than the control (P ≤ 0.05; Fig. 3).

When comparing non-saline versus saline soils for the first-year crop, a difference was observed for control treatment only. The concentration of carotenoids was 37 % higher in stigmas of the control treatment of saline soil than in the control treatment of non-saline soil (P ≤ 0.05; Fig. 3). However, when comparing non-saline versus saline soils for the second-year crop, the concentration of carotenoids was significantly higher by 1–6 % under non-saline than saline conditions for all treatments except for *PM-B* treatment (P ≤ 0.05; Fig. 3).

#### 3.3.2 Total polyphenolics

For the first-year crop grown in non-saline soil, except *PM-B* treatment, all other fertilizer treatments significantly increased the concentration of total polyphenolics in stigma as compared to control by 25–31 % (P ≤ 0.05; Fig. 3). For the second-year crop grown in non-saline soil, *FYM* and *PM* amendments significantly increased the concentration of total polyphenolics than control treatment by 4 and 7 % respectively (P ≤ 0.05; Fig. 3). All other treatments did not show a significant difference than control in this regard (Fig. 3).

For first-year crop grown in saline soil, there was no difference between treatments regarding the concentration of total polyphenolics. For the second-year crop, as compared to the control, *FYM* treatment significantly increased the concentration of total polyphenolics by 12 % (P ≤ 0.05; Fig. 3).

When comparing non-saline with saline soil for a given treatment, no differences were observed for both year's crops (Fig. 3). When comparing first versus second-year crops, for non-saline soil, except control treatment, the concentration of total polyphenolics was higher by 14.5–23 % in various treatments for first than second-year crops. Likewise, for saline soil, except *FYM* and *PM* treatments, the concentration of total polyphenolics in the first-year crop was higher by 20 to 37 % for various treatments than second-year crop (P ≤ 0.05; Fig. 3).

#### 3.3.3 Flavonoids

For the first-year crop grown in non-saline soil, no difference between treatments was observed; whereas for the second-year crop, amendment of *SG-B* increased the concentration of flavonoids by 35, 43, 15 and 33 % than control, *PM*, *FYM-B* and *PM-B* treatments respectively (P ≤ 0.05; Fig. 3).

For the first-year crop grown in saline soil, no difference between treatments was observed except that under *FYM* amendment, the concentration of flavonoids was significantly higher by ∼ 11 % than *SG* treatment (P ≤ 0.05; Fig. 3). For the second-year crop grown in saline soil, the concentration of flavonoids in stigma under *PM* treatment was significantly lower than control, *SG* and *FYM* treatments by 20.6, 8 and 7 % respectively (P ≤ 0.05; Fig. 3).

When comparing non-saline versus saline soil, the concentration of flavonoids in stigma under *SG-B* and *FYM-B* treatments was significantly higher by 33 and 18 % respectively in non-saline than saline soil (P ≤ 0.05; Fig. 3). As observed for carotenoids, second-year crops had significantly higher concentrations of flavonoids than first-year crops in both soils (non-saline and saline) (P ≤ 0.05; Fig. 3) except for PM treatment in non-saline soil, which showed a nonsignificant difference (Fig. 3).

### 3.4 Diversity indices of bacteria in the rhizosphere soil of saffron corms

**Fig. 4:**
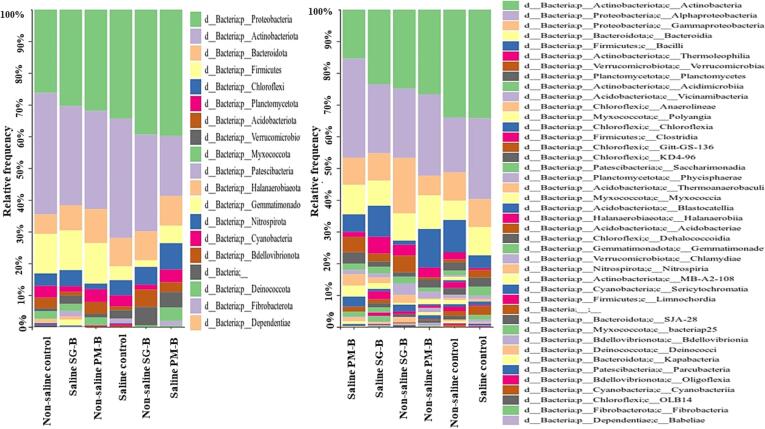
Bacterial groups in rhizosphere soils of various treatment groups, i.e., *control*, *SG-B*, and *PM-B* treatments. Groups are made of non-saline and saline soils for a given treatment.

**Fig. 5:**
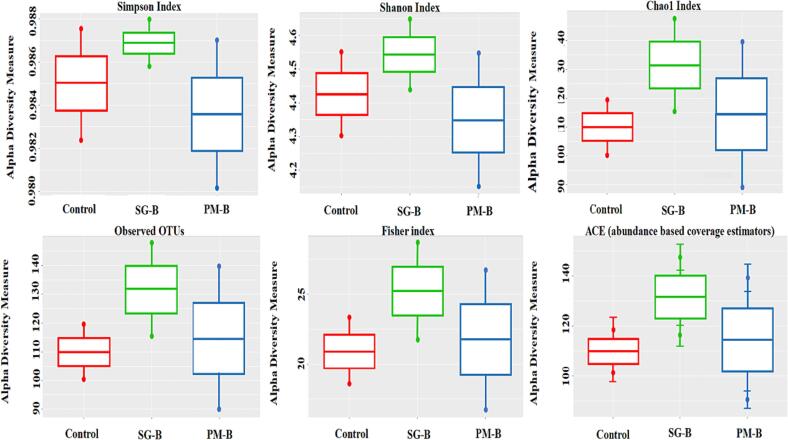
Simpson, Shannon, Chao1, Observed OTUs, Fisher and ACE alpha diversity indexes of rhizosphere soils of three groups, i.e., *Control*, *SG-B*, and *PM-B* treatments. Groups are made of non-saline and saline soils for a given treatment.

**Fig. 6:**
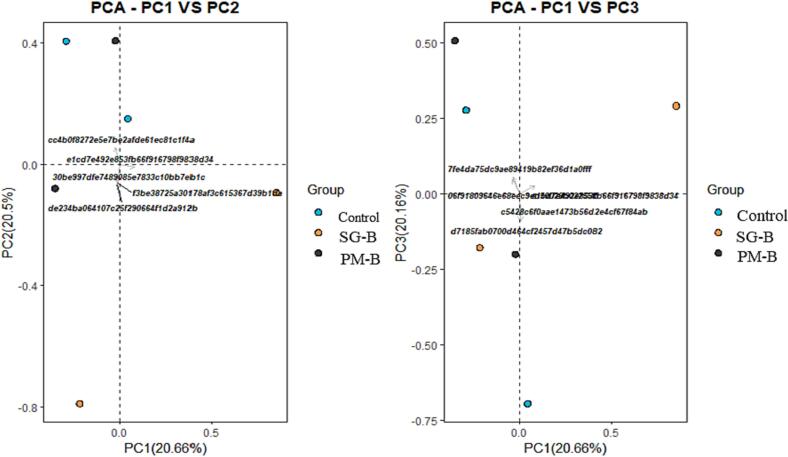
Principal component analysis of beta diversity of bacterial species in various treatments, i.e., *Control* of saline and non-saline soils, *PM-B* of saline and non-saline soils, and *SG-B* of saline and non-saline soils.

The rhizosphere samples had 19 bacterial phyla. The abundance of Chloroflexi over Firmicutes was higher in saline control treatment. The amendment of *PM-B* in saline soil did not reduce, whereas the amendment of *SG-B* in saline soil reduced the abundance of Chloroflexi over Firmicutes (Fig. 4). For both non-saline and saline soils, diversity index such as the community richness (Shannon), Observed OTUs, estimated OTUs richness (Chao 1) and Simpson matrices were 3–4 times higher in *PM-B* and *SG-B* treatments than control treatment (Fig. 5 and Fig. 6). Moreover, 32 bacterial families and 347 bacterial species were found in the rhizosphere soils of *PM-B* and *SG-B* treatments that were not present in control treatments (Table 5 and Table 6). Interestingly, findings are not consistent for a given treatment across soils (non-saline and saline); for instance, if a bacterial family or bacterial species that is found in *PM-B*-amended saline rhizosphere soil is not found in *PM-B*-amended non-saline rhizosphere soil.

**Table 5:** Bacterial families that were not found in the rhizosphere soil of control treatments and were found in the fertilizer-amended rhizosphere soils (collected after the second cropping season).

**Bacterial family**	***SG-B-NS***	***PM-B-NS***	***SG-B-S***	***PM-B-S***
Acetobacteraceae	–	✔	–	–
Anaerolineaceae	–	✔	–	–
Azospirillaceae	–	–	–	–
Babeliaceae	–	–	✔	–
Cellulomonadaceae	✔	–	–	–
Clostridiaceae	–	✔	–	–
Devosiaceae	✔	✔	✔	✔
Fibrobacteraceae	–	–	✔	–
Flavobacteriaceae	–	✔	✔	✔
Gaiellaceae	–	✔	✔	–
Garciellaceae	–	–	✔	–
Gemmatimonadaceae	–	–	✔	–
Haloplasmataceae	–	–	✔	–
Hungateiclostridiaceae	–	–	✔	–
Inquilinaceae	✔	–	–	✔
Legionellaceae	–	–	–	–
Limnochordia	–	–	✔	–
Microbulbiferaceae	–	–	✔	–
Myxococcaceae	–	–	–	–
Nannocystaceae	✔	–	✔	✔
Neisseriaceae	✔	–	–	–
Nocardiopsaceae		✔	–	–
Opitutaceae	✔	–	✔	✔
Oxalobacteraceae	✔	✔	–	✔
Peptococcaceae	–	–	✔	–
Phycisphaeraceae	–	–	–	✔
Promicromonosporaceae	–	✔	✔	–
Pseudohongiellaceae	✔	–	✔	–
Rhodobacteraceae	✔	✔	✔	✔
Trueperaceae	–	–	–	✔
Verrucomicrobiaceae	✔	✔	✔	✔
Weeksellaceae	–	–	✔	–

SG; sheep/goat manure, B; biochar, NS; non-saline, S; saline, PM; poultry manure, -- No data (not present), ✔ indicates the presence of family.

**Table 6:** Bacterial species that did not found in the rhizosphere soil of control treatments and were found in fertilizers amended rhizosphere soils (collected after second cropping year).

**Bacterial species**	***SG-B-NS***	***PM-B-NS***	***SG-B-S***	***PM-B-S***
*Clostridium ultunense*	–	–	✔	–
*Acidobacteria bacterium*	✔	–	–	–
*Agromyce indicus*	✔	✔	✔	–
*Algoriphagus resistens*	–	–	✔	✔
*Allorhizobium oryzae*	–	–	✔	–
*Aquimonas* sp.	✔	–	–	–
*Arenimonas daejeonensis*	✔	–	✔	–
*Arthrobacter crystallopoietes*	–	–	–	✔
*Bacillus alkalitelluris*	–	✔	–	✔
*Bacillus funiculus*	–	✔	–	–
*Bacillus sinesaloumensis*	–	–	✔	–
*Candidate division*	–	–	✔	–
*Devosia neptuniae*	–	–	✔	–
*Empedobacter* sp.	–	–	✔	–
*Flavobacteriaceae bacterium*	–	✔	–	–
*Flavobacterium qiangtangense*	–	✔	–	–
*Hyphomicrobium* sp.	–	–	–	✔
*Luteibacter* sp.	✔	–	–	–
*Luteolibacter arcticus*	✔	–	–	–
*Microbulbifer okinawensis*	–	–	✔	–
*Mycoavidus cysteinexigens*	✔	–	–	–
*Nannocystis exedens*	–	–	–	✔
*Niastella* sp.	–	–	✔	–
*Nocardioides dilutus*	✔	–	–	–
*Parachlamydia* sp.	✔	–	✔	–
*Parapedobacter pyrenivorans*	–	–	✔	–
*Planctomyces* sp.	✔	✔	–	–
*Pseudomonas* sp.	✔	✔	–	–
*Pseudoxanthomonas dokdonensis*	✔	–	–	–
*Roseimicrobium gellanilyticum*	–	–	–	✔
*Roseomonas lacus*	–	–	✔	–
*Rubrobacterineae bacterium*	✔	–	–	–
*Solirubrobacterales bacterium*	–	–	✔	✔
*Sphingomonas sanxanigenens*	–	–	✔	–
*Tissierella* sp.	–	✔	–	–
*Uncultured Acidimicrobiales*	–	–	✔	–
*Uncultured Actinobacteridae*	✔	–	–	–
*Uncultured beta*	✔	–	–	–
*Uncultured Candidatus*	–	–	✔	–
*Uncultured Chlorobiales*	–	✔	–	–
*Uncultured delta*	–	–	✔	–
*Uncultured Desulfovibrionales*	–	✔	–	–
*Uncultured Flavobacteriaceae*	–	–	✔	✔
*Uncultured Flexibacter*	–	–	✔	–
*Uncultured forest*	–	✔	–	–
*Uncultured gamma*	✔	✔	–	–
*Uncultured Hyphomicrobium*	–	–	–	✔
*Uncultured organism*	✔	–	–	–
*Uncultured Pedobacter*	–	–	✔	–
*Uncultured Pirellula*	✔	–	–	–
*Uncultured Planctomycetales*	✔	✔	✔	–
*Uncultured planctomycete*	–	✔	✔	–
*Uncultured proteobacterium*	–	✔	–	–
*Uncultured Rhodospirillales*	–	–	✔	–
*Uncultured sludge*	–	✔	–	–
*Uncultured Sphingobacterium*	–	–	✔	–
*Uncultured Thermomonas*	–	✔	–	✔
*Uncultured Verrucomicrobia*	✔	–	✔	✔
*Wastewater metagenome*	–	✔	–	–

SG; sheep/goat manure, B; biochar, NS; non-saline, S; saline, PM; poultry manure, -- No data (not found), ✔ indicates the presence of bacterial species.

## 4 Discussion

### 4.1 Fresh yield of stigma

In non-saline soil, for first-year crop only, all fertilizers significantly reduced the yield of saffron stigma than the control treatment. The reduction was in the range of 15–49 % for various treatments. Furthermore, in our study, the highest reduction in yield of first-year crops was observed for *PM-B* than control treatment (49 % reduction) in non-saline soil. Biochar can improve crop yield when it is applied to soil with synthetic and organic fertilizers or as a co-composted fertilizer (Gul and Whalen, 2016, El-Mageed et al., 2021). There are empirical pieces of evidence that suggest the negative influence of biochar on various crops when it was applied as a sole fertilizer in soil (Semida et al., 2019, Wang et al., 2019). However, no published report is available to date that shows the negative influence of biochar on the yield of saffron, when it was applied as a co-compost or as a mixture with poultry manure. Contrary to that, there is ample data, which show the positive influence of a mixture of poultry manure with biochar on other crops (Lashari et al., 2014, Adekiya et al., 2019, Altaf et al., 2021, Siddiqui et al., 2021). For example, in a study conducted in a Research Farm at Landmark University, Nigeria, Adekiya et al. (2019) reported that the application of slow-pyrolyzed wood-derived biochar (production source; hardwood from *Khaya senegalensis*, *Parkia biglobosa*, *Terminalia glaucescens* and *Prosopis africana*), when mixed with poultry manure at 25 t ha^−1^ biochar and 2.5 t ha^−1^ manure or at 25 t ha^−1^ biochar and 5 t ha^−1^ manure rates, enhanced radish root weight by 62 and 65 % respectively for first-year crop and by 67 and 71 % respectively for the second-year crop as compared to control. In another study conducted in arid El Fayoum province, Cairo, Egypt, El-Mageed et al. (2021) found that the application of poultry manure biochar as co-compost with soil, geranium waste, and mature compost at 5 and 10 t ha^−1^ rates, increased the yield of eggplant by 24.5 and 40 % respectively, then control. Our results are in contradiction to the published reports that found the positive influence of poultry manure-biochar-based fertilizers on crop growth. However, a reduction of 35–36 % in the grain yield of maize was reported by Mekuria et al. (2014) in response to the amendment of rice husk biochar, co-composted with cow manure.

Crop grown in saline soil, no reduction in the yield of saffron stigma was observed in response to the amendment of organic fertilizers. The non-saline soil of our study had a lower concentration of organic matter (9.9 g kg^−1^ soil) than saline soil (30.9 g kg^−1^ soil). The literature review by Gul and Whalen (2016) suggests that the beneficial influence of biochar-based fertilizers is more evident in soils that have high concentrations of organic matter. Van Zweiten et al. (2010) reported a significant reduction in the biomass of wheat and radish when slow-pyrolyzed paper-mill biochar was applied in calcarosol, which had 20.3 g kg^−1^ soil organic carbon. However, the same biochar significantly improved the biomass of soybean and radish in ferrosol, which had a higher concentration of organic carbon (36.0 g kg^−1^ soil). We applied organic fertilizers in mid-August (18 – 08 – 2020), just a few weeks before the emergence of leaves (observed on October 18, 2020). It is reported that biochar captures nutrients and acts as a slow-release fertilizer (Kammann et al., 2015). It is possible that low doses (15 t ha ^1^) of these fertilizers might have adsorbed nutrients from non-saline soil, which had already a low concentration of organic matter and in return caused nutrient limitation to plants, which may explain the reduction in the yield of stigma. Unfortunately, no empirical evidence exists that proves our hypothesis, this area of study is worth future investigation regarding the application rate of biochar-based composted fertilizers in low-fertility soils.

In non-saline soil, no difference between treatments was found for the yield of the saffron stigma of the second-year crop, whereas fertilizers significantly reduced the yield of the first-year crop. This indicates that continuous second-year amendment of these fertilizers did not further reduce yield than control. Contrary to the findings for non-saline soil, no difference between treatments was observed for the yield of the stigma of first-year crop in saline soil. However, for the second-year crop grown in saline soil; as compared to the control, *FYM* and *PM* treatments significantly increased stigma yield by 44 and 41 % respectively. These results for both soils indicate that continuous amendments of these fertilizers over time improve the yield of this crop. Furthermore, our findings also indicate that manures from cow and buffalo farms and poultry manure have some potential to improve saffron stigma yield in this saline soil. Our results agree with the previous findings. For instance, in a three-year field study in Shirvan county, Iran, Bicharanloo et al. (2021) found that poultry manure applied at 5,859 kg ha^−1^ rate caused a significant 30 % increase in the dry stigma yield than a control treatment for the first-year crop. In a two-year field study conducted in Shahrekord, Iran, Aboueshaghi et al. (2022) found that the amendment of poultry manure at 3 t ha^−1^ rate increased stigma fresh weight by 22 and 25 % than control for first- and second-year crop respectively. No study to date is available about the influence of co-composted farmyard manure on saffron stigma yield. In our study, although PM increased the stigma yield of saffron in saline soil; this fertilizer, however, reduced the concentration of carotenoids and flavonoids in the stigma. On the other side, *FYM* only reduced the concentration of carotenoids but increased total polyphenolics and had no influence on the concentration of flavonoids, for second-year crop in saline soil. This study shows that *FYM* can be an option to improve the stigma yield of saffron in this saline soil.

### 4.2 Morphology and number of corms

Corms grown in non-saline soil had smooth surfaces; whereas corms grown in saline soil had covers of dead tissues, which may be an adaptive feature of saffron to protect living tissues of corm from salinity. For the crop grown in non-saline soil, no difference between treatments was observed regarding the weight of corms for 0–5, >5–10 and > 10–15 g weight classes. Our results for non-saline soil are not in agreement with the findings of Daneshmandi and Seyyedi (2019). They reported a significant increase in the number of corms of each weight class (0–5, >5–10 and > 10–15 g) and observed this increase with increasing the amendment rate of poultry manure (3, 6 and 9 t ha^−1^). However, we found that corms of a larger weight class of > 15–20 g were higher in *PM-B* than *PM* treatment, which shows that in non-saline soil, co-composted biochar with poultry manure improved corm quality than poultry manure. The size and weight of saffron corm are important determinants of the multiplication of progeny, flower production and length of stigma (Douglas et al., 2014, Cardone et al., 2021). Corms with larger weights and sizes produce longer stigma lengths and dry weight (Douglas et al., 2014, Cardone et al., 2021).

In our study, however, for saline soil, the number of corms of lower weight class (0–5 g) was significantly higher under *PM* treatment than control, *SG* and *SG-B* treatments by approximately 56 %. The number of corms of higher weight class (>10–15 g) was significantly higher under treatment of *SG-B* than *SG*, *PM*, *FYM-B* and *PM-B* by 58, 75, 50 and 83 % respectively, whereas corm weight class of > 15–20 g was significantly higher under treatment of *SG* than *PM-B* by 83 %. It shows the differential influence of fertilizers on corm weight classes; where *PM* promoted the number of corms of lower weight while *SG* and *SG-B* promoted the number of corms of higher weight than other organic fertilizers.

Interestingly, for the first-year crop in non-saline soil, the total number of corms under treatments of *SG-B* and *FYM-B* was significantly lower than the control by 33 %. However, no difference was observed for the second-year crop in this regard. This shows the positive influence of these co-composted fertilizers on corm production over time. In non-saline soil, PM fertilizer did not improve the total number of corms for the first-year crop; however, for the second-year crop, this fertilizer significantly increased the total number of corms as compared to control, *FYM-B* and *PM-B* by 29, 40 and 31 % respectively. Bicharanloo et al. (2021) also reported a non-significant influence of poultry manure on the total number of corms for the first-year crop but found a significant increase for the second and third-year crop as compared to the control treatment. In our study, for the first-year crop grown in saline soil, amendment of *SG-B* fertilizer significantly increased the number of corms than control, *PM*, and *PM-B* by 33, 40 and 49 %; whereas, for the second-year crop, the total number of corms was significantly higher under treatment of *PM* fertilizer amendment than *SG*, *FYM* and *SG-B* by 30, 30 and 36 % respectively. Our findings indicate that these fertilizers did not show a consistent trend for the growth of corms over space (soils with different salinity levels) and time as was observed for the fresh yield of stigma.

### 4.3 Carotenoids, total polyphenolics, and flavonoids in saffron stigma

#### 4.3.1 Carotenoids

In our study, significant differences were observed regarding the concentration of carotenoids in stigmas of saffron in response to the amendment of different fertilizers in both soils (non-saline and saline). Biochar-based organic amendments influence the concentration of carotenoids in the stigmas of saffron. For example, a field study of over three years, conducted at Shahid Bahonar University, Kerman, Iran, demonstrated that the cattle manure compost mixture with slow-pyrolyzed biochar from wood wastes of forest as soil amendment did not change the concentration of crocin and picrocrocin but significantly increased the concentration of safranal in the stigmas of saffron than control treatment (2019). In our study, as observed for the fresh yield of stigma and the number and weight of corms, our results for carotenoids and other antioxidants were not consistent over space (soils with different salinity levels) and time (years of cultivation). For instance, for the first-year crop grown in non-saline soil, all fertilizers significantly increased the concentration of carotenoids by 35 to 42 % than the control. However, for the second-year crop, except for *SG* and its co-compost with biochar (*SG-B*), all other fertilizers significantly reduced the concentration of carotenoids by ∼ 3 to 7 % than the control. The stigma of this expensive spice has a high concentration of carotenoids, and these antioxidants have potent antitumor/anticancer properties (Hoshyar and Bathaie, 2013, José Bagur et al., 2018). Carotenoids are also the direct and indirect precursors of biosynthesis of other important secondary metabolites such as α-cyclocitral and β-Cyclocitral, which give colour, aroma, and flavour to this spice (Condurso et al., 2017). In our study, although the reduction in the concentration of carotenoids in stigmas of second-year crop grown in non-saline soil, under various organic fertilizers was marginal (∼3 to 7 %), such reductions can alter the quality of saffron and its commercial value. Moradi et al. (2019) also reported a reduction in the concentration of crocin (a kind of carotenoid) and an increase in the concentration of picrocrocin (a precursor of safranal biosynthesis) in stigmas of saffron in beeswax biochar-amended soil. In our study, co-composted biochar *FYM-B* and *PM-B* had 1.5 and 2.4 % significantly lower concentrations than *FYM* and *PM* fertilizers respectively for second-year crop cultivated. The long-term effects of co-composted biochar on the quality of saffron stigma merits further investigations to get an insight into the utility of these fertilizers for saffron cultivation on a commercial scale.

In saline soil, for the first-year crop, plants grown under *PM* and *FYM-B* had 5 to 11 % lower concentrations of carotenoids in stigmas than *SG* and *SG-B*, whereas no difference between fertilizers and control was observed in this regard. For the second-year crop, as observed for non-saline soil, most of the fertilizer treatments (except *FYM*), reduced the concentration of carotenoids by 1.1 to 1.6 % than the control. However, for the second-year crop, *SG* and *SG-B* treatments in non-saline and *FYM* treatment in saline soil caused no difference from than control. The *meta*-analysis of Gul and Whalen (2016) suggested that biochar-based fertilizers exert differential influence on cop growth performance; whereas this study indicates that differential influence can be observed under different soil conditions also.

When comparing non-saline versus saline soil conditions for a given treatment, for the second-year crop for all treatments (except *PM-B* treatment), the concentration of carotenoids was significantly higher by 1 to 6 % under non-saline than saline soil conditions. This indicates that salinity stress may have played a role in this regard and application of these organic fertilizers did not play a positive role. Differential influence of organic fertilizers especially biochar-based amendments on crop growth performance is expected (Gul and Whalen, 2016) and is frequently reported (Mavi et al., 2018, Abagandura et al., 2019, Omara et al., 2020). However, the quality of antioxidants of saffron stigma being differentially influenced by organic fertilizers regarding soil type is not documented.

#### 4.3.2 Total polyphenolics

As was observed for carotenoids, for first-year crop grown in non-saline soil, all fertilizer treatments (except *PM-B* with no significant difference), significantly increased the concentration of total polyphenolics in stigma than control by 25 to 31 % while no difference between fertilizer treatments was seen. For the second-year crop; however, *FYM* and *PM* amendments significantly increased the concentration of total polyphenolics than in the control treatment by 4 and 7 % respectively while no difference between fertilizer treatments was observed. These results are not in agreement with the results of the effect of these fertilizers on the concentration of carotenoids in second-year crop, where these treatments caused a significant reduction to control. These findings show the non-consistent influence of these organic amendments, over space and time, on the concentration of various antioxidants in the stigma of saffron. Our results agree with the findings of Ghanbari et al. (2019). They found that under field conditions, amendment of cattle manure compost at 20 t ha^−1^ rate caused no influence on the concentration of total polyphenolics and flavonoids in the saffron stigma: whereas co-amendment of compost with wood-based biochar as 10 (compost) + 8 (biochar) t ha^−1^ significantly reduced polyphenolics but increased flavonoids in stigma of saffron.

For the first-year crop grown in saline soil, there was no difference between treatments for the concentration of total polyphenolics in saffron stigma, whereas, for the second-year crop, differences were observed only between *FYM* and control and *PM-B* treatments. The concentration under *FYM* treatment was significantly higher than control and *PM-B* treatments by ∼ 12 %. Interestingly, this treatment also significantly increased the concentration of polyphenolics in second-year crops grown in non-saline soil. This consistent result may have implications for its use as a soil amendment for saffron cultivation. Our findings are in agreement with the results of Ghanbari et al. (2019). These authors reported a significant positive influence of cattle manure compost and its mixture with biochar on the concentration of total polyphenolics in the stigma of saffron for second-year crop.

#### 4.3.3 Flavonoids

No difference between treatments was observed for the first-year crop grown in non-saline soil, whereas, for the second-year crop, amendment of *SG-B* increased the concentration of flavonoids by 35, 43, 15 and 33 % than control, *PM*, *FYM-B* and *PM-B* respectively. Interestingly, as compared to *PM* fertilizer, *SG-B* also significantly increased the concentration of carotenoids for the second-year crop in non-saline soil. Our results indicate that co-composted biochar with the manure from sheep and goats increased the concentration of flavonoids and carotenoids than *PM* fertilizer. Although the findings of Bicharanlou et al. (2017) suggested that manure from cattle is better than the manure from sheep regarding the yield of saffron, our results show that over two years of study, *FYM* and its co-composting with biochar did not improve saffron stigma yield but as compared to SG and *SG-B*, reduced the concentration of carotenoids in non-saline soil. However, in contrast to the results for non-saline soil, for first-year crop grown in saline soil, *FYM* amendment increased the concentration of flavonoids than *SG* fertilizer by 11 %; whereas no difference between these treatments was observed for fresh yield of stigma. It suggests that fertilizer-specific influences on the quality of saffron stigma also depend to some extent on soil type. Another interesting result is that, for the second-year crop in saline soil, the concentration of flavonoids in the stigma of saffron grown under *PM* treatment, was significantly lower than control, *SG* and *FYM* by 20.6, 8 and 7 % respectively. The *PM* fertilizer also reduced the concentration of carotenoids for second-year crop than control treatment in both soils (non-saline and saline soils). Although for second-year crop, in saline soil, this treatment significantly increased stigma yield than control and did not influence stigma yield in non-saline soil, its significantly negative influence on the concentration of carotenoids and flavonoids merits further investigation regarding its overall influence on the quality of stigma of this expensive spice.

### 4.4 Bacterial diversity in the rhizosphere of saffron corms

The metagenome analysis for bacteria revealed that rhizosphere soils of tested samples had 19 bacterial phyla, 42 orders, 146 families, more than 200 genera and more than 300 species. The dominant phyla in all samples were Proteobacteria > Actinobacteria > Bacteroidota. In non-saline soil, an abundance of Firmicutes was greater than Chloroflexi, whereas, in saline soil, Chloroflexi was in greater abundance than Firmicutes except for the treatment *SG-B*. Our results are consistent with published empirical evidence that bacterial phyla Chloroflexi tolerate saline soils (Yousuf et al., 2012). However, in saline soil, *SG-B* treatment was reduced, whereas PM-B increased the abundance of Chloroflexi more than control. These treatments however did not cause any influence on soil electrical conductivity (data unpublished). Therefore, these findings cannot be related to the factor of salinity.

Another interesting finding was that 347 bacterial species were found only in the rhizosphere of corms grown under *SG-B* and *PM-B* treatments and those species were not found in control treatments. However, not all these species were found in each sample, which indicates that these fertilizers may cause a differential influence on bacterial community structure in different soils. Bacterial community structure plays an important role in the quality of saffron stigma (Ghayoumi et al., 2022). However, despite promoting a very high number of bacterial species and high bacterial diversity in the rhizosphere of saffron corms, these treatments did not increase stigma yield. Our findings are based on two years study. Published reports reveal the positive influence of biochar-based organic fertilizers on the yield and quality of saffron stigma after long-term (two to three years or more) continuous amendments in soil (Ghanbari et al., 2019, Bicharanloo et al., 2021). Due to time and fund limitations, we did not perform this experiment for more than two years and did not carry out rhizosphere metagenome analysis for all the treatments over time. It merits further investigation to evaluate microbial diversity dynamics over time after continuous amendments of co-composted biochar fertilizers in saline soil and to study if bacterial diversity dynamics influence saffron stigma yield and its quality.

## 5 Conclusions

The influence of various organic fertilizers on the yield and concentration of carotenoids, total polyphenolics and flavonoids was different over space (soils with different salinity levels) and time. In non-saline soil, reduced stigma yield for the first year and nonsignificant influence for the second-year crop were observed in response to the amendment of organic fertilizers. For the second-year crop in non-saline soil, co-composted biochar with the manure from sheep/goat (*SG-B*), although did not increase yield and total number of corms, but significantly increased the concentration of carotenoids and flavonoids, suggests that this treatment improved the quality of saffron stigma in this soil. For the saline soil, although PM fertilizer significantly increased the yield of saffron stigma and the total number of corms than a control for the second-year crop, this treatment, however, reduced the concentration of carotenoids and flavonoids in stigma. Contrary to this effect of *PM* treatment, *FYM* in saline soil significantly increased the yield and the concentration of total polyphenolics for the second-year crop. This suggests that *FYM* may be an option to improve yield and the quality of saffron stigma in saline soil. Although, *SG-B* and *PM-B* greatly increased bacterial diversity in the rhizosphere of corms in both soils, this positive influence did not improve the yield or quality of saffron stigma. The long-term (more than 2 years) influence of these fertilizers on the yield and quality of saffron stigma may be investigated about their influence on bacterial diversity in the rhizosphere soil of this medicinal crop in saline soil.

## Declaration of competing interest

The authors declare that they have no known competing financial interests or personal relationships that could have appeared to influence the work reported in this paper.
