# Supplementary material for: Biochar-based organic fertilizers: Influence on yield and concentration of antioxidants in the stigma of saffron and rhizosphere bacterial diversity of slightly saline and non-saline soils
Source: Saudi J Biol Sci. 2024 Jan 5;31(3):103922. doi: 10.1016/j.sjbs.2023.103922 (PMC10826820; doi:10.1016/j.sjbs.2023.103922)
Supplement: Supplementary data 1 [file mmc1.docx]

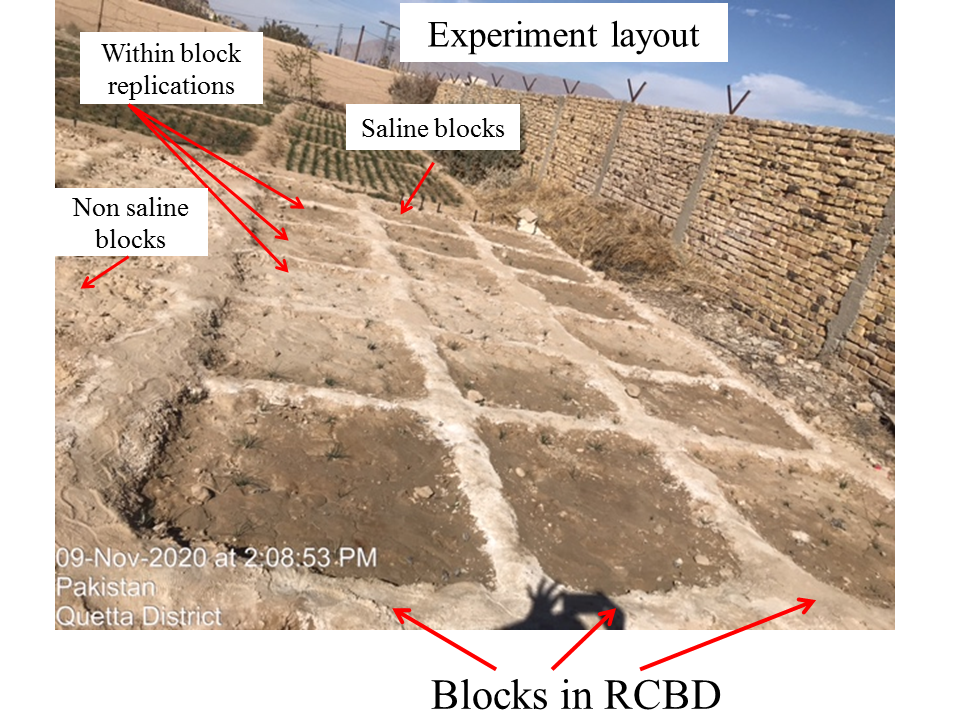


Supplementary Figure S1: Experiment layout for saffron growth


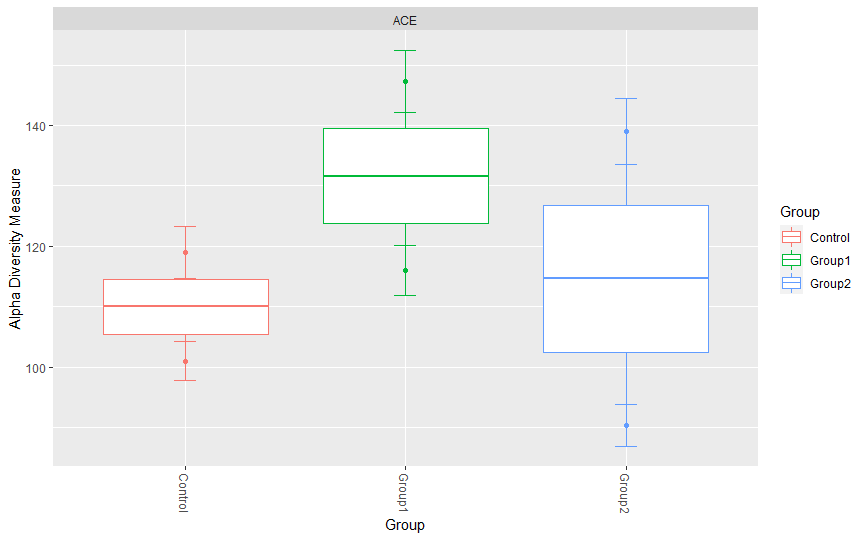


Group 1 - SG-B, Group 2 - PM-B


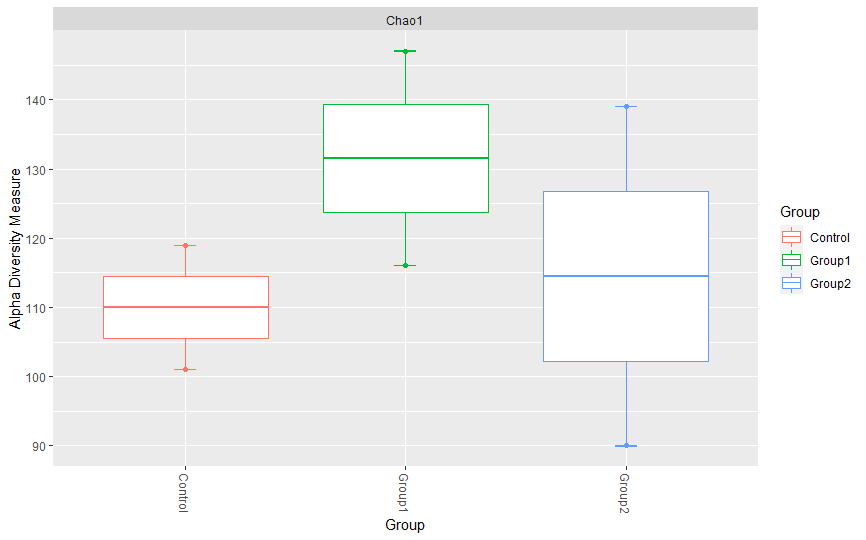


Group 1 is SG-B and group 2 is PM-B


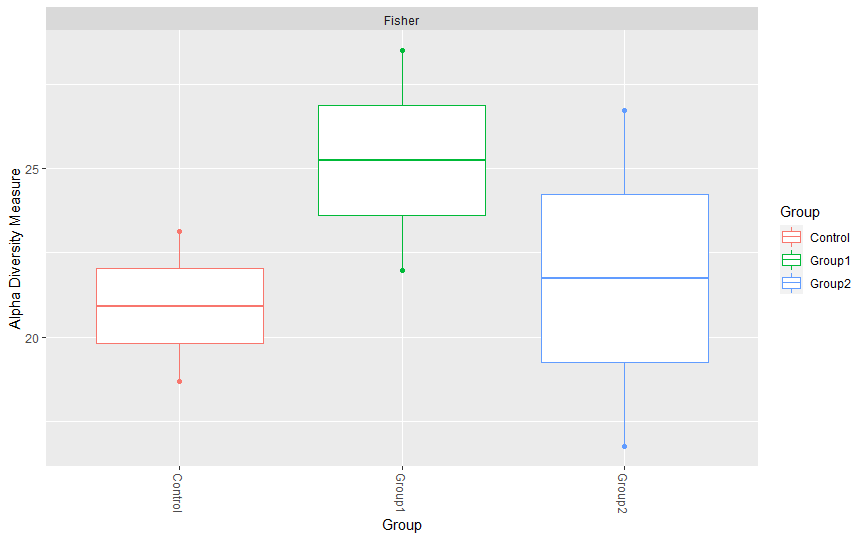


Group 1 is SG-B and group 2 is PM-B


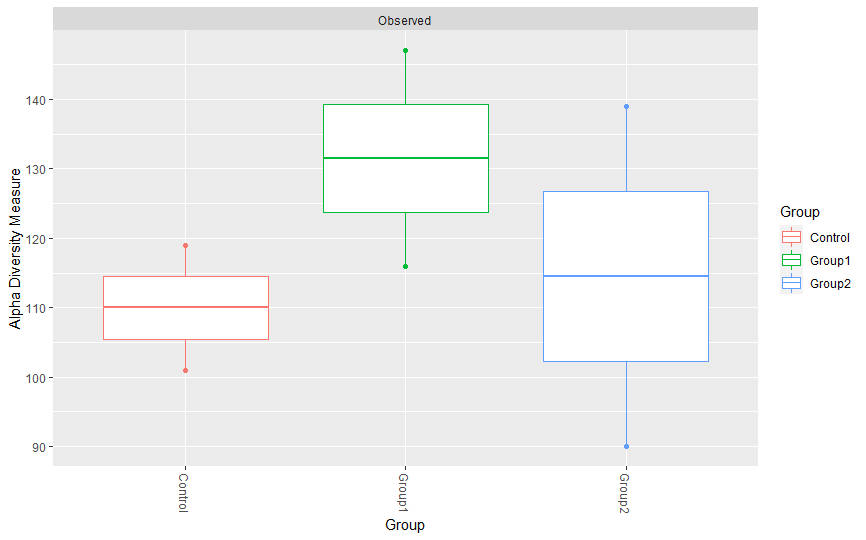


Group 1 is SG-B and group 2 is PM-B


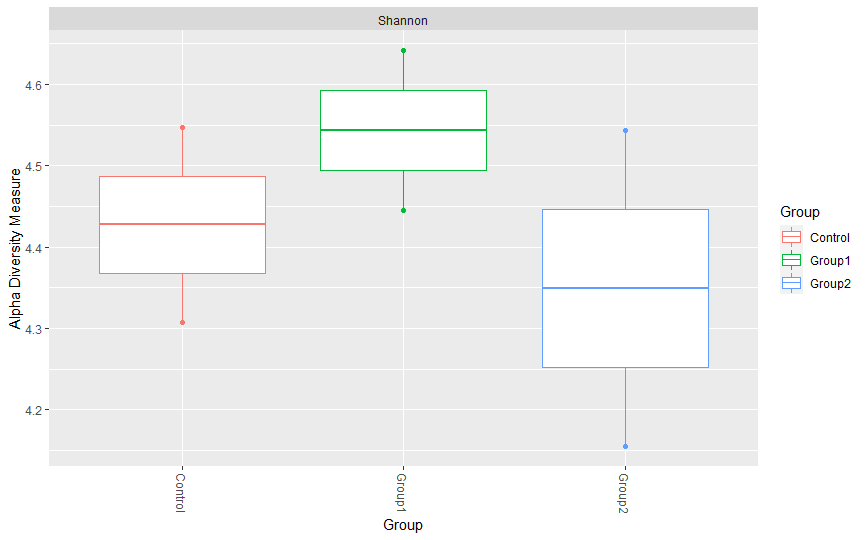


Group 1 is SG-B and group 2 is PM-B


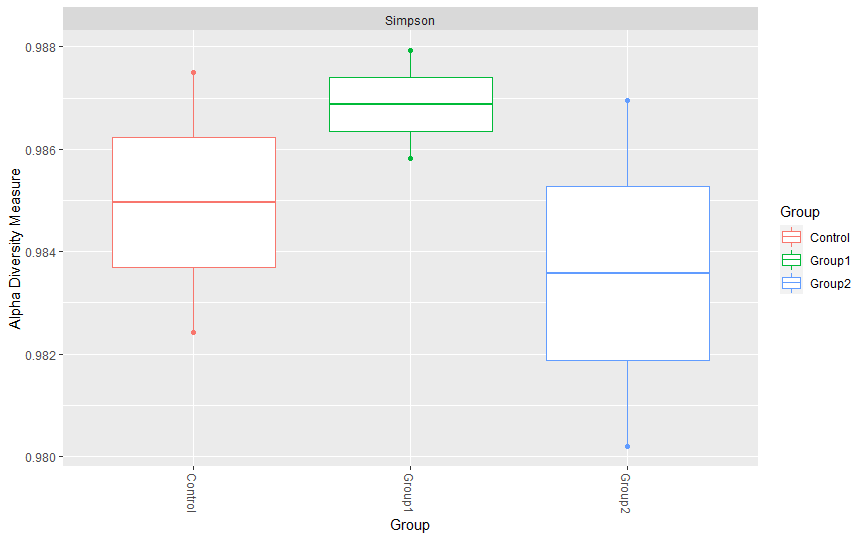


Group 1 is SG-B and group 2 is PM-B

| **Table S1: Stigma fresh weight (g) non-saline soil first year sampling (2020)** | | | | | | | | | | | | | | | | | | | | | |
| --- | --- | --- | --- | --- | --- | --- | --- | --- | --- | --- | --- | --- | --- | --- | --- | --- | --- | --- | --- | --- | --- |
|  | **Control** | | | **SG** | | | **FYM** | | | **SG-B** | | | **FYM-B** | | | **PM-B** | | | **PM** | | |
| Date | **R1** | **R2** | **R3** | **R1** | **R2** | **R3** | **R1** | **R2** | **R3** | **R1** | **R2** | **R3** | **R1** | **R2** | **R3** | **R1** | **R2** | **R3** | **R1** | **R2** | **R3** |
| 28.10.2020 | 0 | 0 | 0 | 0 | 0 | 0 | 0 | 0 | 0 | 0 | 0 | 0 | 0 | 0.06 | 0 | 0 | 0 | 0 | 0 | 0 | 0 |
| 29.10.2020 | 0 | 0 | 0 | 0 | 0 | 0 | 0 | 0 | 0 | 0 | 0.1 | 0 | 0 | 0 | 0 | 0 | 0 | 0 | 0 | 0 | 0 |
| 30.10.2020 | 0 | 0 | 0 | 0 | 0 | 0 | 0 | 0 | 0 | 0 | 0 | 0 | 0 | 0 | 0 | 0 | 0 | 0 | 0 | 0 | 0 |
| 31.10.2020 | 0.1 | 0.1 | 0.5 | 0.2 | 0 | 0 | 0.02 | 0.06 | 0 | 0 | 0.1 | 0 | 0 | 0.1 | 0.02 | 0.006 | 0 | 0 | 0.07 | 0 | 0.04 |
| 1&2. 11.2020 | 0.24 | 0.3 | 0.09 | 0.1 | 0.09 | 0.1 | 0.1 | 0.2 | 0.05 | 0.07 | 0.1 | 0.05 | 0.2 | 0.3 | 0.08 | 0.1 | 0.04 | 0.1 | 0.1 | 0.2 | 0.1 |
| 3& 4. 11.2020 | 0.2 | 0.3 | 0.2 | 0.2 | 0.1 | 0.09 | 0.2 | 0.2 | 0.1 | 0.07 | 0.2 | 0.07 | 0.2 | 0.05 | 0.07 | 0.1 | 0.1 | 0.1 | 0.2 | 0.1 | 0.2 |
| 5.11.2020 | 0.1 | 0.1 | 0.06 | 0.1 | 0.2 | 0.2 | 0.02 | 0.1 | 0.1 | 0.08 | 0.1 | 0.02 | 0.05 | 0.1 | 0.1 | 0.02 | 0.1 | 0.1 | 0.02 | 0.05 | 0.2 |
| 6.11.2020 | 0.09 | 0 | 0.07 | 0.04 | 0.1 | 0.06 | 0.04 | 0 | 0.04 | 0.02 | 0.1 | 0.09 | 0.1 | 0.1 | 0.1 | 0.05 | 0.05 | 0.02 | 0.006 | 0.07 | 0.1 |
| 9.11.2020 | 0.1 | 0.1 | 0.2 | 0.1 | 0.1 | 0.2 | 0.04 | 0.2 | 0.1 | 0.1 | 0.1 | 0.1 | 0.1 | 0.02 | 0.09 | 0.02 | 0.05 | 0.1 | 0.08 | 0.1 | 0.1 |
| 10.11.2020 | 0.06 | 0 | 0.03 | 0.05 | 0.02 | 0.07 | 0.09 | 0 | 0 | 0.9 | 0.03 | 0.06 | 0.02 | 0 | 0.02 | 0 | 0 | 0.02 | 0 | 0.1 | 0.03 |
| 11.11.2020 | 0 | 0 | 0.02 | 0.02 | 0.05 | 0.08 | 0.04 | 0.02 | 0.08 | 0.01 | 0 | 0.02 | 0 | 0 | 0 | 0 | 0 | 0 | 0.09 | 0.05 | 0.02 |
| 12.11.2020 | 0 | 0 | 0.04 | 0 | 0.02 | 0.06 | 0.02 | 0 | 0.1 | 0 | 0 | 0.09 | 0 | 0.05 | 0.08 | 0 | 0.04 | 0.04 | 0 | 0.09 | 0.04 |
| 13.11.2020 | 0.06 | 0 | 0.05 | 0 | 0.02 | 0 | 0 | 0 | 0.1 | 0.02 | 0 | 0.07 | 0.05 | 0.01 | 0.09 | 0 | 0.04 | 0.02 | 0.03 | 0.03 | 0 |
| 16.11.2020 | 0 | 0 | 0.02 | 0 | 0 | 0 | 0 | 0 | 0.08 | 0.01 | 0 | 0 | 0.06 | 0 | 0 | 0 | 0 | 0.04 | 0.02 | 0.06 | 0.02 |
| 17.11.2020 | 0 | 0 | 0 | 0 | 0 | 0 | 0 | 0 | 0 | 0 | 0 | 0 | 0 | 0 | 0 | 0 | 0 | 0 | 0 | 0 | 0 |
| 18.11.2020 | 0 | 0 | 0 | 0 | 0 | 0 | 0 | 0 | 0 | 0 | 0 | 0 | 0 | 0 | 0.01 | 0 | 0 | 0 | 0 | 0 | 0 |
| 23.11.2020 | 0 | 0 | 0.01 | 0 | 0 | 0 | 0 | 0 | 0 | 0 | 0 | 0 | 0 | 0 | 0 | 0 | 0 | 0 | 0 | 0 | 0 |
| **Sum** | **0.95** | **0.9** | **1.29** | **0.81** | **0.7** | **0.86** | **0.57** | **0.78** | **0.75** | **1.28** | **0.83** | **0.57** | **0.78** | **0.79** | **0.66** | **0.296** | **0.42** | **0.54** | **0.616** | **0.85** | **0.85** |

| **Table S2: Fresh weight (g) stigma of saffron grown in saline soil, first year data (2020)** | | | | | | | | | | | | | | | | | | | | |
| --- | --- | --- | --- | --- | --- | --- | --- | --- | --- | --- | --- | --- | --- | --- | --- | --- | --- | --- | --- | --- |
| **Control** | | | **SG** | | | **FYM** | | | **SG-B** | | | **FYM-B** | | | **PM-B** | | | **PM** | | |
| **R1** | **R2** | **R3** | **R1** | **R2** | **R3** | **R1** | **R2** | **R3** | **R1** | **R2** | **R3** | **R1** | **R2** | **R3** | **R1** | **R2** | **R3** | **R1** | **R2** | **R3** |
| 0 | 0 | 0 | 0 | 0 | 0 | 0 | 0 | 0 | 0 | 0 | 0 | 0 | 0 | 0 | 0 | 0 | 0 | 0 | 0 | 0 |
| 0 | 0 | 0 | 0 | 0 | 0 | 0 | 0 | 0 | 0 | 0 | 0 | 0 | 0 | 0 | 0 | 0 | 0 | 0 | 0 | 0 |
| 0 | 0 | 0 | 0 | 0 | 0 | 0 | 0 | 0 | 0 | 0 | 0 | 0 | 0 | 0 | 0 | 0 | 0 | 0 | 0 | 0 |
| 0 | 0 | 0 | 0 | 0 | 0 | 0 | 0 | 0 | 0 | 0 | 0 | 0 | 0 | 0 | 0 | 0 | 0 | 0 | 0 | 0 |
| 0.04 | 0.009 | 0.06 | 0.09 | 0.07 | 0.03 | 0.08 | 0.05 | 0.01 | 0 | 0.07 | 0.02 | 0.09 | 0.02 | 0.05 | 0.1 | 0 | 0.1 | 0.1 | 0.08 | 0.09 |
| 0.3 | 0.1 | 0.1 | 0.2 | 0.1 | 0.1 | 0.1 | 0.3 | 0.06 | 0.08 | 0.1 | 0.1 | 0.3 | 0.2 | 0.1 | 0.2 | 0.1 | 0.2 | 0.2 | 0.2 | 0.2 |
| 0.07 | 0.1 | 0.05 | 0.07 | 0.07 | 0.07 | 0.09 | 0.06 | 0.1 | 0.01 | 0.1 | 0.03 | 0.1 | 0.09 | 0.02 | 0.08 | 0.08 | 0.1 | 0.07 | 0.1 | 0.02 |
| 0.05 | 0.05 | 0.1 | 0.02 | 0.1 | 0.09 | 0.05 | 0.2 | 0.02 | 0 | 0.08 | 0.06 | 0.1 | 0.06 | 0.05 | 0.1 | 0.1 | 0.05 | 0.02 | 0.1 | 0.1 |
| 0.4 | 0.2 | 0.07 | 0.1 | 0.1 | 0.1 | 0.2 | 0.04 | 0.09 | 0.05 | 0.1 | 0.1 | 0.1 | 0.2 | 0.1 | 0.1 | 0.1 | 0.1 | 0.09 | 0.2 | 0.1 |
| 0.09 | 0.04 | 0 | 0.02 | 0 | 0.05 | 0.02 | 0 | 0.04 | 0 | 0 | 0.04 | 0.03 | 0.02 | 0 | 0.07 | 0.07 | 0.06 | 0.04 | 0 | 0.04 |
| 0.07 | 0 | 0.02 | 0.02 | 0.03 | 0 | 0.04 | 0.06 | 0 | 0.02 | 0.04 | 0 | 0.04 | 0.02 | 0 | 0.04 | 0.02 | 0 | 0 | 0.04 | 0.1 |
| 0.04 | 0.07 | 0.02 | 0.03 | 0.04 | 0 | 0.02 | 0.09 | 0 | 0.1 | 0.04 | 0 | 0.01 | 0.07 | 0.05 | 0.04 | 0.007 | 0.04 | 0.01 | 0.05 | 0.02 |
| 0 | 0.07 | 0.03 | 0 | 0.04 | 0.02 | 0.06 | 0.07 | 0.02 | 0.07 | 0.02 | 0.02 | 0 | 0.02 | 0 | 0 | 0.04 | 0 | 0.01 | 0.03 | 0 |
| 0 | 0.08 | 0.01 | 0.03 | 0.02 | 0 | 0.04 | 0 | 0 | 0.02 | 0.02 | 0 | 0 | 0 | 0.05 | 0.06 | 0.01 | 0 | 0.01 | 0 | 0 |
| 0 | 0 | 0 | 0 | 0 | 0 | 0 | 0 | 0 | 0 | 0 | 0 | 0 | 0 | 0 | 0 | 0 | 0 | 0 | 0 | 0 |
| 0 | 0.04 | 0 | 0 | 0 | 0 | 0 | 0 | 0 | 0.02 | 0 | 0 | 0 | 0 | 0 | 0 | 0 | 0 | 0 | 0.02 | 0.01 |
| 0 | 0 | 0 | 0 | 0 | 0 | 0 | 0 | 0 | 0.02 |  | 0.01 | 0 | 0.02 | 0 | 0 | 0 | 0 | 0 | 0.05 | 0.01 |
| **1.06** | **0.759** | **0.46** | **0.58** | **0.57** | **0.46** | **0.7** | **0.87** | **0.34** | **0.39** | **0.57** | **0.38** | **0.77** | **0.72** | **0.42** | **0.79** | **0.527** | **0.65** | **0.55** | **0.87** | **0.69** |

| **Table S3: Stigma fresh wright, non-saline soil, second year data (2021)** | | | | | | | | | | | | | | | | | | | | | |
| --- | --- | --- | --- | --- | --- | --- | --- | --- | --- | --- | --- | --- | --- | --- | --- | --- | --- | --- | --- | --- | --- |
|  | **Control** | | | **SG** | | | **FYM** | | | **SG-B** | | | **FYM-B** | | | **PM-B** | | | **PM** | | |
| Date | **R1** | **R2** | **R3** | **R1** | **R2** | **R3** | **R1** | **R2** | **R3** | **R1** | **R2** | **R3** | **R1** | **R2** | **R3** | **R1** | **R2** | **R3** | **R1** | **R2** | **R3** |
| 20.10.2021 | 0 | 0 | 0 | 0 | 0 | 0 | 0 | 0 | 0 | 0 | 0 | 0 | 0.06 | 0 | 0 | 0 | 0 | 0 | 0 | 0 | 0 |
| 21.10.2021 | 0 | 0 | 0 | 0 | 0 | 0 | 0 | 0 | 0 | 0 | 0 | 0 | 0.04 | 0 | 0 | 0 | 0 | 0 | 0 | 0 | 0 |
| 22.10.2021 | 0 | 0 | 0 | 0 | 0 | 0 | 0 | 0 | 0 | 0 | 0 | 0 | 0.22 | 0 | 0 | 0 | 0 | 0 | 0 | 0 | 0 |
| 25.10.2021 | 0.08 | 0.12 | 0 | 0 | 0 | 0 | 0 | 0 | 0 | 0.2 | 0 | 0 | 0.72 | 0.54 | 0 | 0 | 0.14 | 0 | 0 | 0.05 | 0 |
| 26.10.2021 | 0.2 | 0 | 0 | 0 | 0 | 0 | 0 | 0 | 0 | 0 | 0 | 0 | 0.18 | 0 | 0 | 0 | 0 | 0 | 0 | 0 | 0 |
| 27.10.2021 | 0.06 | 0.07 | 0.08 | 0.08 | 0.06 | 0.14 | 0.05 | 0 | 0.08 | 0.07 | 0.11 | 0.1 | 0.07 | 0.08 | 0.08 | 0.08 | 0.19 | 0.07 | 0.08 | 0.06 | 0.07 |
| 28.10.2021 | 0.11 | 0.27 | 0.03 | 0 | 0.04 | 0 | 0 | 0.08 | 0.13 | 0.11 | 0.17 | 0 | 0.57 | 0.25 | 0 | 0.18 | 0.19 | 0 | 0 | 0.05 | 0.27 |
| 29.10.2021 | 0.28 | 0.14 | 0.29 | 0.18 | 0.26 | 0.09 | 0.21 | 0.48 | 0.46 | 0.18 | 0.38 | 0.14 | 0.2 | 0.13 | 0.1 | 0.07 | 0.09 | 0.07 | 0.05 | 0.2 | 0.43 |
| 01.11.2021 | 0.4 | 0.303 | 0.65 | 0.29 | 0.19 | 0.04 | 0.26 | 0.45 | 0.14 | 0.41 | 0.36 | 0.16 | 0.09 | 0.22 | 0.11 | 0.233 | 0.08 | 0.11 | 0.166 | 0.37 | 0.38 |
| 02.11.2021 | 0.16 | 0.129 | 0.33 | 0.27 | 0.12 | 0.22 | 0.28 | 0.13 | 0.19 | 0.49 | 0.11 | 0.13 | 0.1 | 0.13 | 0.11 | 0.917 | 0.13 | 0.13 | 0.412 | 0.17 | 0.2 |
| 03.11.2021 | 0.04 | 0.052 | 0.04 | 0.13 | 0.03 | 0.1 | 0.11 | 0.16 | 0 | 0.08 | 0.13 | 0.05 | 0.02 | 0 | 0 | 0.077 | 0.31 | 0.07 | 0.026 | 0.07 | 0.06 |
| 04.11.2021 | 0.11 | 0.107 | 0.11 | 0.09 | 0.11 | 0.15 | 0.08 | 0.02 | 0.13 | 0.11 | 0.06 | 0.11 | 0.05 | 0.31 | 0.02 | 0.143 | 0.11 | 0.02 | 0.172 | 0.11 | 0.1 |
| 05.11.2021 | 0 | 0 | 0.09 | 0 | 0 | 0 | 0.03 | 0.04 | 0 | 0 | 0 | 0 | 0 | 0 | 0 | 0.05 | 0 | 0 | 0 | 0.01 | 0 |
| 08.11.2021 | 0.03 | 0 | 0.02 | 0 | 0 | 0 | 0.02 | 0.09 | 0.02 | 0.03 | 0.12 | 0.21 | 0.02 | 0 | 0 | 0.047 | 0 | 0 | 0.036 | 0.04 | 0 |
| 09.11.2021 | 0 | 0.3 | 0.31 | 0.31 | 0.3 | 0.05 | 0.04 | 0.09 | 0 | 0.39 | 0 | 0.05 | 0.31 | 0 | 0.05 | 0 | 0.03 | 0.04 | 0 | 0 | 0.34 |
| 10.11.2021 | 0.03 | 0 | 0 | 0.29 | 0 | 0 | 0 | 0 | 0 | 0 | 0.01 | 0 | 0 | 0 | 0 | 0 | 0 | 0 | 0.013 | 0.01 | 0 |
| 11.11.2021 | 0.02 | 0 | 0 | 0 | 0 | 0 | 0 | 0 | 0 | 0 | 0 | 0 | 0.03 | 0 | 0 | 0.027 | 0 | 0 | 0 | 0 | 0 |
| 12.11.2021 | 0 | 0 | 0 | 0 | 0 | 0 | 0 | 0 | 0.05 | 0 | 0 | 0 | 0 | 0 | 0 | 0 | 0 | 0.05 | 0 | 0 | 0 |
| **SUM** | **1.52** | **1.491** | **1.95** | **1.64** | **1.11** | **0.79** | **1.07** | **1.55** | **1.19** | **2.08** | **1.45** | **0.95** | **2.67** | **1.66** | **0.46** | **1.824** | **1.27** | **0.55** | **0.955** | **1.14** | **1.85** |

| **Table S4: Saffron stigma weight second year crop (2021)** | | | | | | | | | | | | | | | | | | | | | |
| --- | --- | --- | --- | --- | --- | --- | --- | --- | --- | --- | --- | --- | --- | --- | --- | --- | --- | --- | --- | --- | --- |
|  | **Control** | | | **SG** | | | **FYM** | | | **SG-B** | | | **FYM-B** | | | **PM-B** | | | **PM** | | |
| Date | **R1** | **R2** | **R3** | **R1** | **R2** | **R3** | **R1** | **R2** | **R3** | **R1** | **R2** | **R3** | **R1** | **R2** | **R3** | **R1** | **R2** | **R3** | **R1** | **R2** | **R3** |
| 20.10.2021 | 0 | 0 | 0 | 0 | 0 | 0 | 0 | 0 | 0 | 0 | 0 | 0 | 0 | 0 | 0 | 0 | 0 | 0 | 0 | 0 | 0 |
| 21.10.2021 | 0 | 0 | 0 | 0 | 0 | 0 | 0 | 0 | 0 | 0 | 0 | 0 | 0 | 0 | 0 | 0 | 0 | 0 | 0 | 0 | 0 |
| 22.10.2021 | 0 | 0 | 0 | 0.04 | 0 | 0 | 0 | 0 | 0 | 0 | 0 | 0 | 0 | 0 | 0 | 0 | 0 | 0 | 0 | 0 | 0 |
| 25.10.2021 | 0.12 | 0 | 0 | 0.25 | 0.03 | 0.04 | 0.18 | 0.36 | 0 | 0 | 0.06 | 0 | 0.08 | 0 | 0 | 0.1 | 0 | 0 | 0 | 0 | 0 |
| 26.10.2021 | 0 | 0 | 0 | 0 | 0 | 0.12 | 0.03 | 0 | 0 | 0 | 0.11 | 0 | 0 | 0 | 0 | 0 | 0 | 0 | 0 | 0 | 0 |
| 27.10.2021 | 0.13 | 0.06 | 0.06 | 0.05 | 0.14 | 0.17 | 0.12 | 0.13 | 0.02 | 0.07 | 0.03 | 0.02 | 0.12 | 0.14 | 0.04 | 0.12 | 0.1 | 0.1 | 0.14 | 0.17 | 0.15 |
| 28.10.2021 | 0 | 0.06 | 0.1 | 0.04 | 0 | 0.23 | 0.21 | 0.38 | 0.14 | 0 | 0.03 | 0.06 | 0.15 | 0.1 | 0.03 | 0.14 | 0 | 0 | 0 | 0 | 0 |
| 29.10.2021 | 0.1 | 0.04 | 0.06 | 0.1 | 0.02 | 0.21 | 0.02 | 0.02 | 0 | 0 | 0.13 | 0.13 | 0.25 | 0.13 | 0.07 | 0.21 | 0.02 | 0 | 0.04 | 0.23 | 0.11 |
| 01.11.2021 | 0.12 | 0.116 | 0.12 | 0.12 | 0.04 | 0.09 | 0.05 | 0.19 | 0.15 | 0.05 | 0.05 | 0.09 | 0.46 | 0.33 | 0.25 | 0.27 | 0.186 | 0.17 | 0.22 | 0.13 | 0.2 |
| 02.11.2021 | 0.3 | 0.125 | 0.2 | 0.19 | 0.28 | 0.13 | 0.2 | 0.41 | 0.18 | 0.27 | 0.31 | 0.09 | 0.42 | 0.28 | 0.13 | 0.33 | 0.286 | 0.34 | 0.28 | 0.39 | 0.55 |
| 03.11.2021 | 0.07 | 0.041 | 0.04 | 0.3 | 0.03 | 0.02 | 0.05 | 0.1 | 0.2 | 0.03 | 0.22 | 0.04 | 0.02 | 0.06 | 0.06 | 0.07 | 0.053 | 0.07 | 0.02 | 0.12 | 0.05 |
| 04.11.2021 | 0.08 | 0.071 | 0.07 | 0.1 | 0.09 | 0.03 | 0.24 | 0.08 | 0.2 | 0.08 | 0.04 | 0.1 | 0.05 | 0.07 | 0.08 | 0.05 | 0.079 | 0.08 | 0.2 | 0.5 | 0.08 |
| 05.11.2021 | 0 | 0.013 | 0.02 | 0 | 0 | 0 | 0 | 0 | 0 | 0 | 0 | 0 | 0.03 | 0 | 0 | 0.02 | 0 | 0 | 0 | 0.03 | 0 |
| 08.11.2021 | 0.02 | 0 | 0 | 0.02 | 0.02 | 0 | 0 | 0 | 0 | 0 | 0 | 0 | 0 | 0 | 0 | 0 | 0 | 0 | 0 | 0 | 0 |
| 09.11.2021 | 0 | 0.024 | 0.02 | 0.02 | 0.02 | 0 | 0.2 | 0.02 | 0.02 | 0.03 | 0.02 | 0.07 | 0 | 0 | 0.02 | 0.03 | 0.021 | 0.02 | 0.02 | 0 | 0.02 |
| 10.11.2021 | 0 | 0 | 0 | 0.01 | 0 | 0 | 0 | 0 | 0.02 | 0 | 0 | 0 | 0 | 0.03 | 0 | 0 | 0 | 0 | 0 | 0.02 | 0 |
| 11.11.2021 | 0 | 0 | 0 | 0.02 | 0 | 0 | 0 | 0 | 0 | 0.02 | 0 | 0 | 0 | 0 | 0 | 0.02 | 0 | 0 | 0.02 | 0 | 0 |
| 12.11.2021 | 0 | 0 | 0 | 0 | 0 | 0 | 0 | 0 | 0 | 0 | 0 | 0.04 | 0 | 0 | 0 | 0 | 0 | 0 | 0 | 0 | 0.04 |
| **SUM** | **0.94** | **0.55** | **0.69** | **1.27** | **0.68** | **1.03** | **1.29** | **1.69** | **0.93** | **0.56** | **1** | **0.63** | **1.58** | **1.15** | **0.67** | **1.36** | **0.745** | **0.79** | **0.95** | **1.6** | **1.19** |

| **Table S5: Number of corms per sowing spot of second year data (2021)** | | |
| --- | --- | --- |
|  | **Non-saline soil** | **Saline soil** |
| Treatment | **Number of Corms** | **Number .of Corms** |
| Control | 16 | 16 |
| Control | 14 | 22 |
| Control | 12 | 24 |
| Control | 17 | 18 |
| Control | 22 | 18 |
| Control | 19 | 10 |
| Control | 14 | 11 |
| Control | 17 | 15 |
| Control | 27 | 7 |
| SG | 31 | 13 |
| SG | 29 | 27 |
| SG | 17 | 16 |
| SG | 10 | 14 |
| SG | 26 | 9 |
| SG | 9 | 23 |
| SG | 14 | 15 |
| SG | 18 | 12 |
| SG | 14 | 9 |
| FYM | 14 | 13 |
| FYM | 31 | 27 |
| FYM | 16 | 18 |
| FYM | 15 | 15 |
| FYM | 14 | 8 |
| FYM | 24 | 14 |
| FYM | 14 | 12 |
| FYM | 14 | 17 |
| FYM | 24 | 13 |
| SG-B | 22 | 17 |
| SG-B | 12 | 16 |
| SG-B | 14 | 12 |
| SG-B | 22 | 18 |
| SG-B | 29 | 14 |
| SG-B | 18 | 10 |
| SG-B | 15 | 12 |
| SG-B | 21 | 15 |
| SG-B | 18 | 11 |
| FYM-B | 15 | 16 |
| FYM-B | 16 | 20 |
| FYM-B | 20 | 22 |
| FYM-B | 15 | 11 |
| FYM-B | 9 | 15 |
| FYM-B | 15 | 26 |
| FYM-B | 12 | 18 |
| FYM-B | 16 | 10 |
| FYM-B | 16 | 12 |
| PM-B | 26 | 16 |
| PM-B | 28 | 21 |
| PM-B | 15 | 19 |
| PM-B | 17 | 17 |
| PM-B | 13 | 13 |
| PM-B | 11 | 15 |
| PM-B | 22 | 24 |
| PM-B | 14 | 20 |
| PM-B | 7 | 13 |
| PM | 16 | 12 |
| PM | 13 | 9 |
| PM | 20 | 13 |
| PM | 16 | 19 |
| PM | 23 | 19 |
| PM | 41 | 46 |
| PM | 38 | 30 |
| PM | 43 | 38 |
| PM | 13 | 10 |

| Table S6: Number of corms of each weight class; ≤5g, >5-10g, >10-15g, >15-20g, in non-saline soil. First year crop data only. | | | | | | | | | | | | |
| --- | --- | --- | --- | --- | --- | --- | --- | --- | --- | --- | --- | --- |
|  | R1 | R1 | R1 | R1 | R2 | R2 | R2 | R2 | R3 | R3 | R3 | R3 |
| Treatments | 5g | 10g | 15g | 20g | 5g | 10g | 15g | 20g | 5g | 10g | 15g | 20g |
| Control | 0 | 5 | 3 | 0 | 2 | 3 | 2 | 0 | 0 | 3 | 3 | 1 |
| Control | 1 | 2 | 1 | 3 | 3 | 2 | 5 | 3 | 0 | 3 | 2 | 2 |
| Control | 3 | 4 | 1 | 2 | 1 | 2 | 3 | 1 | 2 | 2 | 0 | 2 |
| SG | 1 | 2 | 4 | 1 | 1 | 4 | 3 | 0 | 1 | 4 | 1 | 3 |
| SG | 3 | 2 | 2 | 0 | 1 | 0 | 1 | 0 | 0 | 1 | 1 | 1 |
| SG | 2 | 2 | 2 | 2 | 2 | 2 | 0 | 0 | 0 | 0 | 2 | 1 |
| FYM | 1 | 1 | 1 | 1 | 1 | 2 | 1 | 0 | 2 | 5 | 2 | 1 |
| FYM | 2 | 1 | 1 | 0 | 1 | 4 | 2 | 0 | 1 | 1 | 6 | 1 |
| FYM | 2 | 1 | 1 | 0 | 0 | 7 | 4 | 1 | 1 | 1 | 2 | 1 |
| SG-B | 1 | 2 | 0 | 0 | 1 | 8 | 0 | 0 | 1 | 1 | 1 | 0 |
| SG-B | 1 | 1 | 2 | 0 | 4 | 0 | 3 | 0 | 2 | 3 | 1 | 0 |
| SG-B | 2 | 2 | 2 | 1 | 1 | 2 | 0 | 0 | 2 | 3 | 1 | 0 |
| FYM-B | 0 | 7 | 1 | 0 | 1 | 3 | 1 | 0 | 3 | 1 | 1 | 0 |
| FYM-B | 2 | 1 | 0 | 0 | 1 | 2 | 0 | 0 | 3 | 2 | 1 | 1 |
| FYM-B | 1 | 3 | 0 | 2 | 0 | 0 | 4 | 0 | 0 | 1 | 3 | 3 |
| PM-B | 3 | 0 | 0 | 1 | 3 | 2 | 0 | 0 | 1 | 2 | 1 | 0 |
| PM-B | 3 | 2 | 3 | 3 | 2 | 2 | 4 | 3 | 1 | 1 | 2 | 0 |
| PM-B | 1 | 2 | 1 | 1 | 2 | 0 | 2 | 4 | 0 | 1 | 2 | 4 |
| PM | 1 | 3 | 2 | 0 | 0 | 5 | 2 | 1 | 0 | 2 | 2 | 0 |
| PM | 7 | 0 | 1 | 0 | 5 | 0 | 0 | 0 | 3 | 3 | 3 | 0 |
| PM | 1 | 0 | 4 | 2 | 1 | 2 | 1 | 1 | 1 | 2 | 2 | 0 |

| Table S7: Number of corms of each weight class; ≤5g, >5-10g, >10-15g, >15-20g, in saline soil. First year crop data only. | | | | | | | | | | | | |
| --- | --- | --- | --- | --- | --- | --- | --- | --- | --- | --- | --- | --- |
|  | R1 | R1 | R1 | R1 | R2 | R2 | R2 | R2 | R3 | R3 | R3 | R3 |
| Treatments | 5g | 10g | 15g | 20g | 5g | 10g | 15g | 20g | 5g | 10g | 15g | 20g |
| Control | 2 | 1 | 1 | 0 | 1 | 0 | 1 | 1 | 1 | 0 | 1 | 0 |
| Control | 0 | 2 | 2 | 2 | 0 | 2 | 0 | 0 | 1 | 1 | 5 | 0 |
| Control | 1 | 0 | 2 | 0 | 0 | 5 | 2 | 0 | 1 | 5 | 2 | 0 |
| SG | 1 | 2 | 1 | 3 | 0 | 1 | 0 | 0 | 1 | 3 | 3 | 0 |
| SG | 0 | 4 | 2 | 3 | 1 | 0 | 1 | 0 | 1 | 2 | 0 | 5 |
| SG | 1 | 2 | 2 | 0 | 0 | 1 | 0 | 1 | 2 | 1 | 1 | 0 |
| FYM | 1 | 2 | 2 | 0 | 1 | 1 | 2 | 0 | 1 | 1 | 2 | 1 |
| FYM | 1 | 3 | 0 | 0 | 1 | 4 | 1 | 0 | 1 | 0 | 1 | 3 |
| FYM | 1 | 3 | 2 | 0 | 0 | 4 | 1 | 2 | 4 | 0 | 2 | 1 |
| SG-B | 0 | 4 | 4 | 0 | 0 | 3 | 1 | 2 | 0 | 3 | 5 | 0 |
| SG-B | 0 | 1 | 0 | 0 | 2 | 2 | 8 | 4 | 0 | 3 | 0 | 2 |
| SG-B | 2 | 2 | 2 | 0 | 0 | 1 | 2 | 2 | 3 | 3 | 2 | 0 |
| FYM-B | 1 | 0 | 3 | 0 | 2 | 2 | 2 | 2 | 1 | 3 | 2 | 0 |
| FYM-B | 1 | 3 | 2 | 0 | 1 | 1 | 0 | 1 | 1 | 3 | 0 | 3 |
| FYM-B | 0 | 7 | 2 | 0 | 3 | 2 | 0 | 0 | 1 | 5 | 1 | 1 |
| PM-B | 1 | 1 | 0 | 0 | 3 | 1 | 0 | 1 | 0 | 0 | 2 | 0 |
| PM-B | 1 | 1 | 0 | 0 | 3 | 1 | 0 | 1 | 0 | 0 | 2 | 0 |
| PM-B | 1 | 3 | 0 | 1 | 1 | 4 | 0 | 0 | 1 | 2 | 0 | 1 |
| PM | 2 | 0 | 0 | 0 | 1 | 2 | 0 | 2 | 5 | 0 | 0 | 0 |
| PM | 1 | 0 | 0 | 0 | 1 | 3 | 2 | 0 | 1 | 6 | 0 | 0 |
| PM | 2 | 1 | 0 | 0 | 1 | 1 | 2 | 0 | 2 | 1 | 2 | 0 |

| Table S8: Concentration of carotenoids, total polyphenolics and flavonoids in the stigma of saffron grown in non-saline soil | | | | | | |
| --- | --- | --- | --- | --- | --- | --- |
| Non saline | 2020 | 2021 | 2020 | 2021 | 2020 | 2021 |
|  | Carotenoids (mg/g DW) | Carotenoids (mg/g DW) | Phenolics (mg/g DW) | Phenolics (mg/g DW) | Flavonoids (mg / g DW) | Flavonoids (mg /g DW) |
| Control | 14.18 | 24.12 | 27.63 | 32.72 | 0.164 | 0.276 |
| Control | 6.70 | 23.77 | 30.00 | 189.8 | 0.117 | 0.294 |
| Control | 16.81 | 24.01 | 35.81 | 33.45 | 0.204 | 0.384 |
| SG | 20.96 | 23.93 | 44.72 | 32.90 | 0.175 | 0.413 |
| SG | 21.42 | 23.86 | 40.54 | 34.00 | 0.150 | 0.359 |
| SG | 20.76 | 23.70 | 41.63 | 34.54 | 0.269 | 0.716 |
| FYM | 20.48 | 23.70 | 38.18 | 34.72 | 0.186 | 0.254 |
| FYM | 22.87 | 23.20 | 54.36 | 35.27 | 0.168 | 0.511 |
| FYM | 21.70 | 23.16 | 42.18 | 33.63 | 0.186 | 0.540 |
| PM | 18.49 | 22.94 | 41.45 | 34.18 | 0.168 | 0.312 |
| PM | 19.63 | 22.82 | 39.45 | 36.72 | 0.175 | 0.276 |
| PM | 20.85 | 22.90 | 45.09 | 36.72 | 3.211 | 0.254 |
| SG-B | 18.55 | 23.83 | 38.54 | 32.54 | 0.186 | 0.478 |
| SG-B | 22.75 | 23.98 | 47.27 | 29.63 | 0.193 | 0.446 |
| SG-B | 20.47 | 23.97 | 40.18 | 32.90 | 0.171 | 0.554 |
| FYM-B | 21.85 | 22.95 | 44.18 | 32.36 | 0.168 | 0.442 |
| FYM-B | 20.10 | 22.95 | 43.09 | 34.00 | 0.208 | 0.428 |
| FYM-B | 20.39 | 23.09 | 37.81 | 35.09 | 0.150 | 0.384 |
| PM-B | 19.73 | 21.57 | 38.54 | 24.72 | 0.197 | 0.323 |
| PM-B | 21.09 | 22.61 | 38.00 | 34.36 | 0.222 | 0.348 |
| PM-B | 17.24 | 22.81 | 34.72 | 35.09 | 0.175 | 0.312 |

|  | | | | | | |
| --- | --- | --- | --- | --- | --- | --- |
| Table S9: Concentration of carotenoids, total polyphenolics and flavonoids in the stigma of saffron grown in saline soil | | | | | | |
|  | 2020 | 2021 | 2020 | 2021 | 2020 | 2021 |
| Treatments | Carotenoids (mg/g DW) | Carotenoids (mg/g DW) | Phenolics (mg/g DW) | Phenolics (mg/g DW) | Flavonoids (mg / g DW) | Flavonoids (mg /g DW) |
| Control | 19.28 | 22.90 | 37.82 | 32.00 | 0.16 | 0.37 |
| Control | 19.14 | 22.90 | 35.82 | 31.64 | 0.21 | 0.39 |
| Control | 22.05 | 22.70 | 50.18 | 32.91 | 0.19 | 0.36 |
| SG | 22.10 | 22.56 | 39.82 | 207.82 | 0.18 | 0.34 |
| SG | 21.46 | 22.43 | 42.36 | 38.55 | 0.16 | 0.37 |
| SG | 21.13 | 22.39 | 40.55 | 32.00 | 0.16 | 0.37 |
| FYM | 19.07 | 22.89 | 33.09 | 38.00 | 0.18 | 0.38 |
| FYM | 20.76 | 22.76 | 37.27 | 33.27 | 0.19 | 0.37 |
| FYM | 21.93 | 22.31 | 42.55 | 38.55 | 0.19 | 0.35 |
| SG-B | 22.15 | 22.49 | 42.91 | 38.00 | 0.19 | 0.38 |
| SG-B | 22.41 | 22.56 | 46.55 | 33.27 | 0.18 | 0.31 |
| SG-B | 21.06 | 22.67 | 39.64 | 31.45 | 0.18 | 0.29 |
| FYM-B | 20.99 | 22.31 | 40.55 | 29.82 | 0.20 | 0.33 |
| FYM-B | 20.41 | 22.49 | 72.00 | 31.64 | 0.15 | 0.33 |
| FYM-B | 19.84 | 22.69 | 37.64 | 33.64 | 0.18 | 0.37 |
| PM-B | 21.59 | 22.56 | 40.00 | 31.82 | 0.19 | 0.37 |
| PM-B | 22.55 | 22.24 | 51.64 | 243.82 | 0.18 | 0.35 |
| PM-B | 19.94 | 22.76 | 33.82 | 32.73 | 0.18 | 0.38 |
| PM | 18.74 | 22.44 | 38.18 | 31.45 | 0.18 | 0.30 |
| PM | 19.95 | 22.62 | 40.73 | 41.64 | 0.19 | 0.30 |
| PM | 19.62 | 22.67 | 36.91 | 33.64 | 0.17 | 0.29 |

| Table S 10: Bacterial families detected in various treatments | | | | | | | |
| --- | --- | --- | --- | --- | --- | --- | --- |
| #OTU ID | SG-B-NS | SG-B-S | PM-B-NS | PM-B-S | Control Saline Soil | Non-saline soil | family |
| d7185fab0700d464cf2457d47b5dc082 | 0 | 0 | 124 | 0 | 0 | 234 | f__Sphingomonadaceae |
| 7fe4da75dc9ae89419b82ef36d1a0fff | 0 | 0 | 0 | 214 | 0 | 0 | f__Sphingomonadaceae |
| cc4b0f8272e5e7be2afde61ec81c1f4a | 0 | 0 | 91 | 0 | 116 | 0 | f__Nocardioidaceae |
| e1cd7e492e853fb66f916798f9838d34 | 196 | 0 | 0 | 0 | 0 | 0 | f__Nocardioidaceae |
| 7923980b51ffb4ccb6f18d1e53a0be86 | 19 | 25 | 31 | 28 | 62 | 26 | f__Micrococcaceae |
| c5428c6f0aae1473b56d2e4cf67f84ab | 0 | 0 | 0 | 0 | 0 | 172 | f__Nocardioidaceae |
| bc1430bdc9a2b6cb953436e71f969745 | 0 | 33 | 59 | 73 | 0 | 0 | f__Rhizobiaceae |
| c55225af0e75ad87bc10c0fc3e719948 | 9 | 29 | 33 | 34 | 31 | 25 | f__Micrococcaceae |
| de234ba064107c25f290664f1d2a912b | 0 | 151 | 0 | 0 | 0 | 0 | f__Geodermatophilaceae |
| f3be38725a30178af3c615367d39b1da | 0 | 146 | 0 | 0 | 0 | 0 | f__Solirubrobacteraceae |
| fc99988b65c9772f674e63a73d5c2b40 | 0 | 0 | 142 | 0 | 0 | 0 | f__Devosiaceae |
| b3cf4a5c3531d61ec80a52fa950714d9 | 0 | 0 | 0 | 142 | 0 | 0 | f__Streptomycetaceae |
| c170a275313d3b07b992368d4f362923 | 0 | 0 | 135 | 0 | 0 | 0 | f__Bacillaceae |
| eb24f9a8cb47003458aaf1f3dd293264 | 0 | 0 | 0 | 0 | 0 | 131 | f__Micromonosporaceae |
| 10df45687fc10d18327f470e38e431b6 | 0 | 0 | 0 | 0 | 0 | 131 | f__Bacillaceae |
| 244d781e5d3b4ae6cb73675661d064de | 0 | 48 | 23 | 0 | 16 | 44 | f__Nocardiaceae |
| 06f91809646e68eec9ed50029b03255b | 0 | 0 | 0 | 71 | 60 | 0 | f__Mycobacteriaceae |
| 30be997dfe7489085e7833c10bb7eb1c | 0 | 125 | 0 | 0 | 0 | 0 | f__Devosiaceae |
| ea0a61307d6dc17c662000b0199e23ba | 0 | 125 | 0 | 0 | 0 | 0 | f__Micrococcaceae |
| c97397ac6462bb8ec967290f811dfddd | 0 | 0 | 0 | 85 | 0 | 39 | f__Xanthobacteraceae |
| 8aa0db72991b55d6b082999f4a9dab6d | 0 | 0 | 122 | 0 | 0 | 0 | f__Geodermatophilaceae |
| 8f8075c02cab44a7cd0b8e7d852abd25 | 0 | 0 | 0 | 0 | 0 | 120 | f__Micrococcaceae |
| a6baa6926204fad76b8acb0d12de5afe | 0 | 0 | 0 | 114 | 0 | 0 | f__Caldilineaceae |
| f907e399af9e0dbf1a6bb91263e382cf | 0 | 0 | 0 | 0 | 113 | 0 | f__Gitt-GS-136 |
| 556b3495ddee561a8676044529b8f6f8 | 0 | 0 | 0 | 113 | 0 | 0 | f__Micrococcaceae |
| 0e6fe837f17fd744fedf314ee992ea39 | 0 | 0 | 109 | 0 | 0 | 0 | f__Nocardioidaceae |
| bbd5fd0bd3c1c5a80ba40caeb392bad3 | 0 | 0 | 108 | 0 | 0 | 0 | f__67-14 |
| bff45b8c5c070a2cb7698645ea1d52fd | 0 | 0 | 0 | 0 | 106 | 0 | f__Sphingomonadaceae |
| 8aa161f26698c863f7d0b457e8d23abd | 0 | 0 | 66 | 0 | 39 | 0 | f__Sphingomonadaceae |
| 0091a0d823647c3159434d7c42ecce36 | 40 | 0 | 64 | 0 | 0 | 0 | f__Xanthomonadaceae |
| 916e822008220f5af0f5bb24fab388d0 | 0 | 104 | 0 | 0 | 0 | 0 | f__Rhizobiaceae |
| 2855119d0b771458ba21dbb711039ead | 39 | 0 | 0 | 0 | 0 | 63 | f__Rhodomicrobiaceae |
| 8d157bda3235ad6a63998384e053fc65 | 0 | 0 | 102 | 0 | 0 | 0 | f__Rhizobiaceae |
| b31bcd8ffe56b005b89aaa69e376246a | 0 | 0 | 102 | 0 | 0 | 0 | f__Nocardioidaceae |
| 5a5d78bfd48bf801bf611deb7620eb20 | 0 | 0 | 0 | 0 | 0 | 101 | f__Nocardioidaceae |
| d05e89d615a1acf9f70ce2d6b1b7fb1f | 0 | 0 | 0 | 0 | 0 | 100 | f__Intrasporangiaceae |
| 396a9909455aceef07c8c680d602fdaf | 0 | 0 | 100 | 0 | 0 | 0 | f__Rhizobiaceae |
| aa92f210f241812cd569c30968caceb9 | 0 | 0 | 0 | 0 | 0 | 97 | f__Geodermatophilaceae |
| 37e8d1991f498e6688512607af8f4823 | 0 | 0 | 0 | 0 | 97 | 0 | f__Sphingobacteriaceae |
| 658919892336d3e1d6dfa44c147ed95b | 96 | 0 | 0 | 0 | 0 | 0 | f__Sphingomonadaceae |
| 9b598230c51aab782c6b2f446ad94061 | 0 | 0 | 0 | 96 | 0 | 0 | f__Sphingomonadaceae |
| 3b2413b4c009bb6fba27b8e014196baf | 0 | 0 | 0 | 0 | 0 | 93 | f__Solirubrobacteraceae |
| 9c4932717b4611696362a46ea5831906 | 0 | 0 | 0 | 93 | 0 | 0 | f__Bacillaceae |
| a7a716d3f1f5068d75ff52092da5b868 | 0 | 0 | 0 | 92 | 0 | 0 | f__Rhizobiaceae |
| 2cb9b8cc10da9544ff8ac2e97be0388f | 0 | 0 | 57 | 0 | 0 | 34 | f__Beijerinckiaceae |
| f7fea7e337301dbc31032687018b70e0 | 91 | 0 | 0 | 0 | 0 | 0 | f__Mycobacteriaceae |
| ff8f1635fad3fc4962045c700d3ca8ec | 0 | 91 | 0 | 0 | 0 | 0 | f__Micrococcaceae |
| 599acc6f1afdd44cc78904c0fe8980fb | 0 | 0 | 0 | 0 | 0 | 89 | f__KD4-96 |
| cc2f9378d537491cd5630efc9fd73f2c | 89 | 0 | 0 | 0 | 0 | 0 | f__Ilumatobacteraceae |
| 11a00aa173e1a059179353abbcdd385e | 0 | 89 | 0 | 0 | 0 | 0 | f__Streptomycetaceae |
| eb817b665babf101b39a77112cd45eac | 0 | 0 | 0 | 88 | 0 | 0 | f__D05-2 |
| 598a91b2745e606e2592a69cb543f1c1 | 0 | 0 | 87 | 0 | 0 | 0 | f__uncultured |
| f28bbc4f2f8cb3acefc461c8fc4f6897 | 0 | 0 | 34 | 0 | 53 | 0 | f__Streptomycetaceae |
| a9def51ab2c51af3ab6012116acd76ea | 45 | 0 | 41 | 0 | 0 | 0 | f__Cyclobacteriaceae |
| de5b441374ec6d5677e1f5ae3c5a6262 | 0 | 86 | 0 | 0 | 0 | 0 | f__Microscillaceae |
| 61c062f4b290724e46f5b65d74767639 | 0 | 0 | 0 | 0 | 0 | 84 | f__Pirellulaceae |
| 294378e89161a13b5f0fb35a1f5179af | 84 | 0 | 0 | 0 | 0 | 0 | f__Rhizobiaceae |
| b2812018067a3f2f9905a48848b8b3bc | 84 | 0 | 0 | 0 | 0 | 0 | f__Micrococcaceae |
| b4dd5a553d1ee799cd4cd784e4eee36c | 0 | 0 | 0 | 84 | 0 | 0 | f__Steroidobacteraceae |
| 3afd6400a21c5af9e56f7b383af9375f | 83 | 0 | 0 | 0 | 0 | 0 | f__Microscillaceae |
| 648c3ac221be7bc96b685d5dd5c51c0a | 0 | 0 | 0 | 83 | 0 | 0 | f__Rhizobiaceae |
| 777cbdcfaecb19ab4a39d7a442743153 | 0 | 83 | 0 | 0 | 0 | 0 | f__Bacillaceae |
| b76cb035aa71a3ea186e1108e04155a6 | 0 | 0 | 54 | 28 | 0 | 0 | f__Beijerinckiaceae |
| 3dd174f634f3ab7817fd9be3ad8d209e | 0 | 82 | 0 | 0 | 0 | 0 | f__Nocardioidaceae |
| b11c5875d09a0b64518a469d346716a3 | 81 | 0 | 0 | 0 | 0 | 0 | f__Beijerinckiaceae |
| b8fc8a43954916ba7a1e3e2649af45f9 | 81 | 0 | 0 | 0 | 0 | 0 | f__Vicinamibacteraceae |
| 043210b0814a3e729fca885444189b3f | 0 | 0 | 0 | 81 | 0 | 0 | f__Rhizobiaceae |
| 5500942e158a52f90622d6d4ce9bcfea | 0 | 0 | 0 | 81 | 0 | 0 | f__Nannocystaceae |
| 815ad6a35693289b5090a5a4f67f8710 | 0 | 0 | 80 | 0 | 0 | 0 | f__Planococcaceae |
| b87467abc21050a7cc8f7fcdfc8e67b1 | 80 | 0 | 0 | 0 | 0 | 0 | f__Azospirillaceae |
| 55e2a73b4d6c091290157e49e6f5caa3 | 0 | 80 | 0 | 0 | 0 | 0 | f__Caldilineaceae |
| 9d788648abe8c195c8de0c390ad7e7a0 | 0 | 0 | 79 | 0 | 0 | 0 | f__Mycobacteriaceae |
| 557fcab2f879b90df5d27d489e5648ee | 0 | 0 | 79 | 0 | 0 | 0 | f__Micrococcaceae |
| d0b6598c22877613069d58dbab2ee899 | 0 | 79 | 0 | 0 | 0 | 0 | f__Gaiellaceae |
| c886aac54206b432a0974d3701274ba7 | 0 | 79 | 0 | 0 | 0 | 0 | f__Sphingomonadaceae |
| 9206a600e32dfa88c94eea9b2f4969c1 | 14 | 7 | 12 | 16 | 20 | 9 | f__Micrococcaceae |
| a3f470b531f45487fe79b5daa3c7f1f9 | 0 | 78 | 0 | 0 | 0 | 0 | f__Sphingomonadaceae |
| 462cadae2665f8d5b0c3a70455629eeb | 0 | 78 | 0 | 0 | 0 | 0 | f__Azospirillaceae |
| caec11b476ff87554acff397ea9e9a08 | 77 | 0 | 0 | 0 | 0 | 0 | f__Solirubrobacteraceae |
| 9ae85ad1250e7ded525a397258a28b5e | 77 | 0 | 0 | 0 | 0 | 0 | f__Opitutaceae |
| 7924307411823b85c3907b1be1b28c62 | 0 | 77 | 0 | 0 | 0 | 0 | f__Streptomycetaceae |
| cf6e6aa8c2d7c329b2496aa077998c8e | 76 | 0 | 0 | 0 | 0 | 0 | f__Nocardioidaceae |
| 1db2422455e8f3df912ab3437a00f06a | 75 | 0 | 0 | 0 | 0 | 0 | f__Hyphomicrobiaceae |
| 2acd6450737e25c9699c8a72e442a172 | 75 | 0 | 0 | 0 | 0 | 0 | f__Mycobacteriaceae |
| 30f43081afcb8086a42aca8e27398e3f | 0 | 75 | 0 | 0 | 0 | 0 | f__Gemmatimonadaceae |
| b3dd863b19db3801af3fa2149563606d | 0 | 0 | 74 | 0 | 0 | 0 | f__Nocardioidaceae |
| 90d7f11719d67def2d2e5cebad369428 | 0 | 0 | 74 | 0 | 0 | 0 | f__Bacillaceae |
| fe63b9e034690a4076b090684effbab3 | 73 | 0 | 0 | 0 | 0 | 0 | f__Devosiaceae |
| 1fefcfaf79cbc4101da0d9f1aa8b1332 | 0 | 0 | 0 | 73 | 0 | 0 | f__Rhizobiaceae |
| cc425fb21f457644b34c6c1678c4ac34 | 72 | 0 | 0 | 0 | 0 | 0 | f__JG30-KF-CM45 |
| 1d6238af1c4b79ad0a4d301ec45edcf1 | 0 | 0 | 71 | 0 | 0 | 0 | f__Nocardioidaceae |
| add8bef816320532820580b08ef57a47 | 0 | 0 | 71 | 0 | 0 | 0 | f__Micrococcaceae |
| c283c69f6716a7ece7e92615d30d2ced | 0 | 0 | 0 | 0 | 71 | 0 | f__Sphingobacteriaceae |
| 0d8ce88f3f9fad127f790f08e52b3514 | 0 | 0 | 0 | 71 | 0 | 0 | f__Devosiaceae |
| 27a48a5fe4071678e76b832fa505fc42 | 70 | 0 | 0 | 0 | 0 | 0 | f__Nocardioidaceae |
| cdb0a1d1d4bc081806b44029bcecd72b | 0 | 0 | 0 | 0 | 70 | 0 | f__Sphingomonadaceae |
| c5c3cb2784127776f95dbb2f117ca221 | 0 | 0 | 0 | 70 | 0 | 0 | f__Pirellulaceae |
| cd1eda4643c23b038819442c68c58a4c | 0 | 0 | 0 | 0 | 0 | 69 | f__Rhizobiaceae |
| 965c32de4c91bac3aa5b343df8e79d2c | 0 | 0 | 0 | 0 | 69 | 0 | f__Planococcaceae |
| 6535e32087e6a29af670d5599131e6d2 | 0 | 0 | 0 | 0 | 69 | 0 | f__Microbacteriaceae |
| f681268aecb67982c8dbea9141dfd0b8 | 0 | 0 | 68 | 0 | 0 | 0 | f__WD2101_soil_group |
| 4bb0726e20f79116635a615f2dd9cc1f | 67 | 0 | 0 | 0 | 0 | 0 | f__Streptomycetaceae |
| 360eaffcc796450933e87c41417f5372 | 67 | 0 | 0 | 0 | 0 | 0 | f__Nocardioidaceae |
| ab795859bfb221917c4869bcda97a327 | 67 | 0 | 0 | 0 | 0 | 0 | f__Caldilineaceae |
| 9c99727d43bff46769b70b91054f5b25 | 0 | 0 | 0 | 67 | 0 | 0 | f__Planococcaceae |
| 6501bceab3629a591b9deb7b1f07761c | 0 | 0 | 0 | 0 | 0 | 66 | f__Nocardioidaceae |
| bc73c66027bacd5cd2d789ef11d440ce | 0 | 0 | 0 | 0 | 0 | 66 | f__Nocardioidaceae |
| 2299fb9ffd84737e9ff4ed08a30bb20a | 0 | 0 | 0 | 0 | 0 | 66 | f__Mycobacteriaceae |
| afa225e647e496ec745521370ed62431 | 0 | 0 | 0 | 0 | 0 | 66 | f__Paenibacillaceae |
| 3b5ebf65eecebf5c7736046f87ad8cd7 | 66 | 0 | 0 | 0 | 0 | 0 | f__Devosiaceae |
| 3e3e4eec9e50a4872136f86f2c241a2b | 0 | 66 | 0 | 0 | 0 | 0 | f__Steroidobacteraceae |
| 2783941706df39d4138058184c23af4c | 0 | 0 | 65 | 0 | 0 | 0 | f__Bacillaceae |
| b87172d81a55fdd290a67f821cd1082a | 65 | 0 | 0 | 0 | 0 | 0 | f__Nocardioidaceae |
| ea010fc184c0e4afee84186bfdfc7b63 | 0 | 65 | 0 | 0 | 0 | 0 | f__KD4-96 |
| 5468d90654c8538e615198ffa331bc53 | 0 | 0 | 0 | 0 | 64 | 0 | f__Sphingomonadaceae |
| 77f278a9b694b4e24d423d3412f2c69c | 0 | 0 | 0 | 0 | 64 | 0 | f__Micrococcaceae |
| 85509d4725df11ea80195e285c31214d | 0 | 64 | 0 | 0 | 0 | 0 | f__Bacillaceae |
| 6b25eed6df765ce879d98a9a3da2d8ed | 0 | 0 | 63 | 0 | 0 | 0 | f__Microscillaceae |
| 91e3267f5ccaea0e662547b07ffe92df | 63 | 0 | 0 | 0 | 0 | 0 | f__Hyphomicrobiaceae |
| 14f321fed6cc24069aa14c327c90884e | 63 | 0 | 0 | 0 | 0 | 0 | f__Thermoanaerobaculaceae |
| 0ca447c539007a0b8610419c7178f751 | 0 | 0 | 0 | 0 | 63 | 0 | f__Rhizobiaceae |
| b335e6c4eaccc9e34161a6f265e4fa67 | 0 | 0 | 0 | 0 | 63 | 0 | f__Nocardioidaceae |
| da1eb90b40a6e4198c0ce7ca54c9d4d1 | 0 | 63 | 0 | 0 | 0 | 0 | f__Rhizobiaceae |
| 5a4b81c23c0c94391937a2d09c11c292 | 0 | 0 | 0 | 0 | 0 | 62 | f__Azospirillaceae |
| 5e813041b43184acd847d8c43daa04e5 | 0 | 0 | 62 | 0 | 0 | 0 | f__Devosiaceae |
| 03baef37d44e996f98f12ed5050bff87 | 0 | 0 | 0 | 0 | 62 | 0 | f__Rhizobiaceae |
| 24dc458655e7885e09183d4401352642 | 0 | 0 | 0 | 62 | 0 | 0 | f__Ilumatobacteraceae |
| 83837c5b6fd6381c9db676256289db72 | 0 | 0 | 0 | 62 | 0 | 0 | f__Microscillaceae |
| 34eaae98386024cd3ae032af7b1eecc4 | 0 | 62 | 0 | 0 | 0 | 0 | f__Gitt-GS-136 |
| 1a9b10d5ed0696d198b1317715ada85b | 0 | 62 | 0 | 0 | 0 | 0 | f__Nocardioidaceae |
| e45d7a2e0c415f5f80840d9aeccfddae | 0 | 0 | 0 | 0 | 0 | 61 | f__Rhizobiaceae |
| a488390ab16ac5529e166a698e380a33 | 0 | 0 | 61 | 0 | 0 | 0 | f__Rhizobiaceae |
| ed4069b2e063a83a43a4b37859c8d15c | 0 | 61 | 0 | 0 | 0 | 0 | f__Promicromonosporaceae |
| 2be9be387e8549f2a12d85fd8ee169c8 | 0 | 61 | 0 | 0 | 0 | 0 | f__Planococcaceae |
| c11918a1924a31fff9b6049733dfeb3a | 0 | 61 | 0 | 0 | 0 | 0 | f__Rhizobiaceae |
| 4cee34a09f59b236a61d34d5e9f39cdf | 60 | 0 | 0 | 0 | 0 | 0 | f__Comamonadaceae |
| d4e027c4ef517cd601bdde815f88bdc5 | 0 | 0 | 0 | 60 | 0 | 0 | f__Hyphomicrobiaceae |
| 9920d00c31da464fa3fe94be8a01a208 | 0 | 0 | 0 | 0 | 0 | 59 | f__Steroidobacteraceae |
| bcd9700d4fc15c838bfe01d0c6b6f0d2 | 0 | 0 | 0 | 0 | 0 | 59 | f__Planococcaceae |
| e42d24cfb93825366121de08f3c9fe98 | 0 | 31 | 0 | 0 | 0 | 28 | f__Peptostreptococcaceae |
| 24d566ca93f1c19f72de1f40c4bb0f22 | 0 | 0 | 59 | 0 | 0 | 0 | f__Pirellulaceae |
| 25003d46de6e6a69d3b9867990b94a0f | 0 | 0 | 0 | 0 | 59 | 0 | f__Alicyclobacillaceae |
| 907b33aca73d876cbd2b2c8c98973462 | 0 | 0 | 0 | 59 | 0 | 0 | f__Mycobacteriaceae |
| c01f26d7a670bebc1b3836841ab30dda | 0 | 59 | 0 | 0 | 0 | 0 | f__uncultured |
| 8b71d2a9f93ad9cafc16609a5c19d569 | 0 | 0 | 0 | 0 | 58 | 0 | f__Sphingomonadaceae |
| cd30c4660f15897daff64ba23c3edf02 | 0 | 0 | 0 | 0 | 58 | 0 | f__Sphingomonadaceae |
| f3c665d9b597e0775cabaa9c6738c31d | 0 | 58 | 0 | 0 | 0 | 0 | f__Pseudonocardiaceae |
| 6ae126bc191cf9205c1cad45defed455 | 0 | 0 | 0 | 0 | 57 | 0 | f__Chitinophagaceae |
| 773266dded97b367a06b5bb7c252cbab | 0 | 0 | 0 | 57 | 0 | 0 | f__Gitt-GS-136 |
| 361ad502a751294aa41caa728170bc67 | 0 | 57 | 0 | 0 | 0 | 0 | f__Xanthomonadaceae |
| 9126defecac389c138629d30c982eb48 | 0 | 57 | 0 | 0 | 0 | 0 | f__uncultured |
| f3386a8006432d95687523c66a66f751 | 0 | 0 | 0 | 0 | 0 | 56 | f__Unknown_Family |
| 3c25a6498a98a8800fd7143739770d6a | 0 | 0 | 0 | 0 | 0 | 56 | f__Microbacteriaceae |
| b98dcf537a4c0c2b395fbfad85512c4d | 0 | 0 | 56 | 0 | 0 | 0 | f__Blastocatellaceae |
| 32e75966e275b68c3c07bfa4d095a9da | 56 | 0 | 0 | 0 | 0 | 0 | f__Xanthomonadaceae |
| 2c3af845cfa49dd79ed12007da5c5122 | 56 | 0 | 0 | 0 | 0 | 0 | f__Streptomycetaceae |
| 8cfe07b3d55b8ce346b9cc42e3a5ec47 | 0 | 56 | 0 | 0 | 0 | 0 | f__Paenibacillaceae |
| 326ad8a86baac6ad65d81bbed87ae506 | 0 | 0 | 0 | 0 | 0 | 55 | f__Halanaerobiaceae |
| edd7b582bbc98963401fb7e38d3391a8 | 0 | 0 | 44 | 0 | 11 | 0 | f__Nocardioidaceae |
| 69bc3b35ea6dbf843a1e5b9a0beb0bdc | 55 | 0 | 0 | 0 | 0 | 0 | f__Nocardioidaceae |
| 594a5ffc5125217753696c0ccb2b8e8b | 55 | 0 | 0 | 0 | 0 | 0 | f__Unknown_Family |
| 9a116d9b108bf6b6f3626bdb6d5b0137 | 55 | 0 | 0 | 0 | 0 | 0 | f__Rhizobiaceae |
| 63dc5d9214c58331a26be0f4dce79c10 | 0 | 55 | 0 | 0 | 0 | 0 | f__Cyclobacteriaceae |
| 460aa5d3918f22d14dfcf5c4be46477f | 0 | 0 | 0 | 0 | 54 | 0 | f__Nocardioidaceae |
| 4e3802a01bc841da4da6e1b758f0043b | 0 | 0 | 0 | 0 | 54 | 0 | f__Glycomycetaceae |
| c7128600d15a28d6616da72f3f3dcc5e | 0 | 0 | 0 | 54 | 0 | 0 | f__Pirellulaceae |
| 2bd4c659591dbc6837cc569fac6c6c47 | 0 | 0 | 0 | 0 | 0 | 53 | f__Streptomycetaceae |
| 4f62887ed0d53e45540384175e83450b | 0 | 0 | 0 | 0 | 19 | 34 | f__Nocardioidaceae |
| c22025f09eb5f1e78a75aa63be5b160c | 0 | 0 | 53 | 0 | 0 | 0 | f__Caulobacteraceae |
| 125948970c265c1fd0ef0d4dc1192c57 | 0 | 0 | 53 | 0 | 0 | 0 | f__Oxalobacteraceae |
| 702dedf591f0fa1c272e4b123059c28b | 0 | 0 | 53 | 0 | 0 | 0 | f__uncultured |
| 8b9e3ee5e8f133db10a412971adca3f4 | 0 | 0 | 0 | 0 | 53 | 0 | f__Nocardioidaceae |
| 97c01b3ece53398798092b242d98c090 | 0 | 0 | 0 | 0 | 53 | 0 | f__Geodermatophilaceae |
| 01990585b45e17df990dbb5384971406 | 0 | 0 | 0 | 0 | 0 | 52 | f__Hyphomicrobiaceae |
| efdbde2e098c28865367d44a998f0cb5 | 52 | 0 | 0 | 0 | 0 | 0 | f__Xanthobacteraceae |
| 3f6d0b3485db5cd259a1c31c5c9361c5 | 0 | 52 | 0 | 0 | 0 | 0 | f__Nocardioidaceae |
| f7fc3c784eed80a44aa41949519494d9 | 0 | 0 | 0 | 0 | 0 | 51 | f__Microscillaceae |
| 2e5b5e2d81f7b276d03a272e0d8854ff | 0 | 0 | 0 | 0 | 0 | 51 | f__Microscillaceae |
| c16be9a8a574e8f694107bf004ad731a | 0 | 0 | 51 | 0 | 0 | 0 | f__Mycobacteriaceae |
| 1a53fdaf19962798d52fde0fe87be470 | 51 | 0 | 0 | 0 | 0 | 0 | f__Xanthomonadaceae |
| 299425985e889226ba78645a9632625f | 0 | 0 | 0 | 0 | 51 | 0 | f__Xanthobacteraceae |
| 7538dbc09a7360938f8df893f8e07d73 | 0 | 0 | 0 | 0 | 51 | 0 | f__Caulobacteraceae |
| 0b79f01dbc564e3f997ed2ed56e10491 | 0 | 51 | 0 | 0 | 0 | 0 | f__Rhizobiaceae |
| c00a4b98032719e55cf7283290a14fd8 | 0 | 0 | 0 | 0 | 0 | 50 | f__S085 |
| 2566e2487d16fecc959f56567675bda7 | 0 | 0 | 50 | 0 | 0 | 0 | f__Flavobacteriaceae |
| 378cb79c32d2a5a05d8e9a7a2b2883b0 | 0 | 0 | 50 | 0 | 0 | 0 | f__Nocardioidaceae |
| 55652458c7c4ffb2db4e9581208be010 | 0 | 0 | 50 | 0 | 0 | 0 | f__Microscillaceae |
| b73f5dccf144623386281bfea004681e | 0 | 0 | 50 | 0 | 0 | 0 | f__Bacillaceae |
| 4ee1f519ea881295e783d7f23dfdb1de | 0 | 50 | 0 | 0 | 0 | 0 | f__Microbacteriaceae |
| ed42a114c72d69e31d2c08535168c778 | 0 | 50 | 0 | 0 | 0 | 0 | f__Halanaerobiaceae |
| dc9481c0920065f838672c05e3d0cd07 | 0 | 0 | 49 | 0 | 0 | 0 | f__Rhizobiaceae |
| b9e881bc1eb53c0eff39cca3f1597f96 | 49 | 0 | 0 | 0 | 0 | 0 | f__Steroidobacteraceae |
| 2b20f121341fc68ec11bd506c94a0281 | 0 | 0 | 0 | 0 | 49 | 0 | f__Streptomycetaceae |
| 763ec14d4ab973ab827c3d05aac9f98b | 0 | 0 | 0 | 49 | 0 | 0 | f__Sphingobacteriaceae |
| 836c33daaf6535ee2641061fd65b81b9 | 0 | 0 | 0 | 49 | 0 | 0 | f__JG30-KF-CM45 |
| 1c4103cc7a0df340b09658e9768b5b7c | 0 | 0 | 48 | 0 | 0 | 0 | f__Flavobacteriaceae |
| 33d57f7cac6c2d38daf26eabed6960a1 | 48 | 0 | 0 | 0 | 0 | 0 | f__Rubritaleaceae |
| ad2a1ad40c0eaadf58cae3b63dc32826 | 0 | 0 | 0 | 0 | 48 | 0 | f__Nocardioidaceae |
| 6b9ce472cf8a92f6691242f02e9961e3 | 0 | 0 | 0 | 48 | 0 | 0 | f__uncultured |
| ddb772a897f302124c79e250b1ba90b2 | 0 | 48 | 0 | 0 | 0 | 0 | f__Steroidobacteraceae |
| 8b5c869a68e2f51383c246aac193837d | 0 | 0 | 0 | 0 | 0 | 47 | f__Alicyclobacillaceae |
| 301493fa2212357b7789de81afc28981 | 0 | 0 | 47 | 0 | 0 | 0 | f__Myxococcaceae |
| daffdded28f29cfcbc1b561c17ac8185 | 0 | 0 | 0 | 0 | 47 | 0 | f__Ilumatobacteraceae |
| bcb0052db81230562c235ea4c07c1b00 | 0 | 0 | 0 | 0 | 47 | 0 | f__Microscillaceae |
| 24e26d94379a9dff41373048ad52aec9 | 0 | 0 | 0 | 26 | 21 | 0 | f__Alcaligenaceae |
| 082b96163cdecb3c2519aa1e2ff554f2 | 0 | 0 | 0 | 47 | 0 | 0 | f__Microscillaceae |
| 05e386f173205e17481822d4c77a7990 | 0 | 0 | 0 | 0 | 0 | 46 | f__Sandaracinaceae |
| e9252ed1fbe56eb1884dd4b2b501c054 | 46 | 0 | 0 | 0 | 0 | 0 | f__Cellulomonadaceae |
| 3e8a03caf9c6c228dcebd6b1a352d303 | 0 | 0 | 0 | 46 | 0 | 0 | f__Cyclobacteriaceae |
| 091a0dbf947e3d56d93f9f19ab976fab | 0 | 0 | 45 | 0 | 0 | 0 | f__Bacillaceae |
| 23dc8815d75c13b48028cdbdb3669fee | 45 | 0 | 0 | 0 | 0 | 0 | f__Rhodanobacteraceae |
| e5b6913f0a0baddb3ff6d0a2e7e295d4 | 0 | 0 | 0 | 0 | 45 | 0 |  |
| d649d80c6f15016f8644b7cfe08d238e | 0 | 0 | 0 | 45 | 0 | 0 | f__67-14 |
| 82de8cb25c53f963c2b6a6f3ef81e229 | 0 | 45 | 0 | 0 | 0 | 0 | f__Verrucomicrobiaceae |
| 036581ca41f9ab0a2979d638f3fbfdb7 | 0 | 0 | 0 | 0 | 0 | 44 | f__WD2101_soil_group |
| 03facaf499a0a68fdf985a1e29b2ebcf | 0 | 0 | 44 | 0 | 0 | 0 | f__Xanthobacteraceae |
| b7d77124eadd3aac038bbb189f927707 | 0 | 0 | 44 | 0 | 0 | 0 | f__Rhizobiaceae |
| 5e9fea0d0f12736643e0a0b58a431399 | 0 | 0 | 44 | 0 | 0 | 0 | f__Hyphomicrobiaceae |
| 1d3a083f3fce2192213a2d9f647a5424 | 44 | 0 | 0 | 0 | 0 | 0 | f__67-14 |
| b1bc01a5f9bdf17392aeb5cd03b5f3a4 | 0 | 0 | 0 | 0 | 44 | 0 | f__Nocardioidaceae |
| 4c09b1fa079132e973521a5599ec79b2 | 0 | 0 | 0 | 0 | 44 | 0 | f__Xanthomonadaceae |
| fc54a2c127cfe20a922746866d664c4a | 0 | 0 | 0 | 0 | 44 | 0 | f__Nocardioidaceae |
| 409a197a0ff95563ade9b4820fa03c66 | 0 | 0 | 0 | 44 | 0 | 0 | f__Nannocystaceae |
| db0b2325bf82bd9f160db091fc437d7d | 0 | 44 | 0 | 0 | 0 | 0 | f__Saccharimonadales |
| 0826bb50cc27946a1c1a1822db9916b2 | 0 | 0 | 0 | 0 | 0 | 43 | f__Nocardioidaceae |
| d8727caf21afb588622ad7e2e70b7a34 | 0 | 0 | 0 | 0 | 0 | 43 | f__Nocardioidaceae |
| ceded2d31498771c1f305c6ad74b7fa9 | 0 | 0 | 0 | 0 | 0 | 43 | f__Xanthomonadaceae |
| 7e494e7eac0aa0a774894ace43432ce1 | 0 | 0 | 43 | 0 | 0 | 0 | f__S085 |
| 97419902850ae622d16300fd0b77db03 | 0 | 0 | 43 | 0 | 0 | 0 | f__Nocardioidaceae |
| 36d887bcc30554a07f57e55e2d4753e1 | 15 | 0 | 28 | 0 | 0 | 0 | f__Pseudomonadaceae |
| 37f65782c2bcdae5d10cb54b61fb1d35 | 43 | 0 | 0 | 0 | 0 | 0 | f__uncultured |
| 1a08bf77f716d31b41bcbadc79db4d36 | 0 | 0 | 0 | 0 | 43 | 0 | f__Micromonosporaceae |
| c2a65d3df280acb960865b1680b2c0e9 | 0 | 0 | 0 | 0 | 0 | 42 | f__Nocardioidaceae |
| ef0adae0c9d74c7a24960af3aacb3d3e | 0 | 0 | 42 | 0 | 0 | 0 | f__Rhizobiaceae |
| e0b5e14221c52958903c97ec9ae3fc2e | 0 | 0 | 42 | 0 | 0 | 0 | f__BIrii41 |
| 47ee1e29e0f7db07e44f07b9207fba22 | 42 | 0 | 0 | 0 | 0 | 0 | f__Xanthomonadaceae |
| fd97dfd310810e3b5e1f2237390d810e | 42 | 0 | 0 | 0 | 0 | 0 | f__Sphingomonadaceae |
| c318e8b99ec0fa8fa9e260da01b976db | 42 | 0 | 0 | 0 | 0 | 0 | f__Rhodanobacteraceae |
| 9b9e6f34f7357568fc0700330da651ce | 0 | 0 | 0 | 0 | 42 | 0 | f__Pirellulaceae |
| 68b80a5bbece6eb88a5252cc7ed4558c | 0 | 0 | 0 | 0 | 42 | 0 | f__Nocardioidaceae |
| d757a3995b879e6dd701c91f3ef7a310 | 0 | 0 | 0 | 42 | 0 | 0 | f__Thermoanaerobaculaceae |
| a943a33a9da9a8728e8ba49140349898 | 0 | 42 | 0 | 0 | 0 | 0 | f__uncultured |
| ef8042cdcd8c441a0793b4c72d150962 | 0 | 0 | 0 | 0 | 0 | 41 | f__Pseudomonadaceae |
| 475538e673aafb18c1327b87a25528cb | 0 | 0 | 41 | 0 | 0 | 0 | f__Microbacteriaceae |
| 957b7b13e31f150e0da15d4fddb3fa18 | 0 | 0 | 41 | 0 | 0 | 0 | f__Nocardioidaceae |
| 55d33be07f696f45fd9485edfa81b773 | 0 | 0 | 41 | 0 | 0 | 0 | f__Sphingobacteriaceae |
| 616092cc1ad2a87b7e5eea7c16043776 | 0 | 0 | 0 | 0 | 41 | 0 | f__Nocardiaceae |
| ffa6a345879b133f0909cbb1f2542033 | 0 | 41 | 0 | 0 | 0 | 0 | f__Hyphomicrobiaceae |
| 53f78cd25cfe3ff7fb7e2086d1263fe7 | 0 | 41 | 0 | 0 | 0 | 0 | f__Pseudomonadaceae |
| 37fa774a4c91ef318e66373db12ca34a | 0 | 0 | 0 | 0 | 0 | 40 | f__Rhizobiaceae |
| 0e09ea7da3f2c8fef353e07ccfcc8fa0 | 0 | 0 | 40 | 0 | 0 | 0 | f__Sphingomonadaceae |
| 94a2e34306b8e9539e9d7b6a500efda3 | 0 | 0 | 40 | 0 | 0 | 0 | f__Bacillaceae |
| 02b1b2a418ea490789b27ad2c2cd7087 | 40 | 0 | 0 | 0 | 0 | 0 | f__KD4-96 |
| 29b03a752fb7536ad86bcd5db1bbc5d5 | 0 | 0 | 0 | 0 | 40 | 0 | f__Xanthomonadaceae |
| c9fad30a91f421a919e97dfe2751ab08 | 0 | 0 | 0 | 0 | 40 | 0 | f__Xanthobacteraceae |
| 565db7ee1a2d26ab6a0a1bb29a232d93 | 0 | 0 | 0 | 0 | 40 | 0 | f__D05-2 |
| a52c924757e376e0924c90c9c3bb8e14 | 0 | 0 | 0 | 0 | 40 | 0 | f__Caulobacteraceae |
| 8446c09ead8c6600737ed629355e1245 | 0 | 0 | 0 | 40 | 0 | 0 | f__Micrococcaceae |
| d4cfeef3b8e9715b695f740b50a5dd8b | 0 | 40 | 0 | 0 | 0 | 0 | f__Xanthobacteraceae |
| 9b05bfc0551b241f29a56737ba4451d5 | 0 | 40 | 0 | 0 | 0 | 0 | f__Bacillaceae |
| daceb9f23f795a99e1e846f4f9fae339 | 0 | 40 | 0 | 0 | 0 | 0 | f__Chitinophagaceae |
| aac96ec24a3eb607f2736c56af8d5644 | 0 | 40 | 0 | 0 | 0 | 0 | f__Nocardioidaceae |
| 7d19e16206ae5a213065cb75318857af | 39 | 0 | 0 | 0 | 0 | 0 | f__Pseudohongiellaceae |
| b8efc12945a51a28894544a8018eb2c9 | 0 | 0 | 0 | 0 | 39 | 0 | f__D05-2 |
| f21aa1757672afb0686842e7ae858f66 | 0 | 39 | 0 | 0 | 0 | 0 | f__67-14 |
| 0fbd93e29ac66c3844217a6fcbd2bf95 | 0 | 39 | 0 | 0 | 0 | 0 | f__Beijerinckiaceae |
| 26a5087e8b88f695bf0d2d916dc85c03 | 0 | 0 | 0 | 0 | 0 | 38 | f__Planococcaceae |
| eb4227c2821375a378a60f35e6759809 | 38 | 0 | 0 | 0 | 0 | 0 | f__Xanthomonadaceae |
| 63126a6afdf3ba6668fe8a158e4971a8 | 0 | 0 | 0 | 38 | 0 | 0 | f__Terrimicrobiaceae |
| 4357e8df96a2b35c4903ebff5319b8d5 | 0 | 38 | 0 | 0 | 0 | 0 | f__Weeksellaceae |
| 57f57e26e5b749073359795da5fce860 | 0 | 0 | 37 | 0 | 0 | 0 | f__Xanthomonadaceae |
| 9b43f6bae5466a8aef4bc42444829eab | 37 | 0 | 0 | 0 | 0 | 0 | f__Rhizobiaceae |
| 4bd08219f1d60ab8a14442ffe6abdd4b | 37 | 0 | 0 | 0 | 0 | 0 | f__Microbacteriaceae |
| 8ab8636c490d02332022c18212df4a01 | 0 | 0 | 0 | 0 | 37 | 0 | f__Nocardioidaceae |
| d5b03f3e5e1575073f88ec105faa4881 | 0 | 0 | 0 | 0 | 37 | 0 | f__Vicinamibacteraceae |
| 4d29d0685ec98f5878835cf2ab784d9e | 0 | 0 | 0 | 0 | 37 | 0 | f__Chthoniobacteraceae |
| 3d94322cdbd5313445329a27bf78a923 | 0 | 37 | 0 | 0 | 0 | 0 | f__AKYG1722 |
| ed9c4b0e142044efaaace57b57158b4e | 0 | 37 | 0 | 0 | 0 | 0 | f__Geminicoccaceae |
| bb34c25c16288e8c33cd9dc61a21ba45 | 0 | 37 | 0 | 0 | 0 | 0 | f__Sphingobacteriaceae |
| 442fad2715993c386088afa9566a2fad | 0 | 0 | 0 | 0 | 0 | 36 | f__Bacillaceae |
| 88e9135512bbaa450d95ef46dc23e41d | 0 | 0 | 0 | 0 | 0 | 36 | f__Bryobacteraceae |
| 6f35e34f7121ac8842eb120a0fcc25e9 | 0 | 0 | 0 | 0 | 0 | 36 | f__Xanthomonadaceae |
| 3456ba7dff876afff47c8c043a31eb5c | 36 | 0 | 0 | 0 | 0 | 0 | f__Xanthobacteraceae |
| 2f12d0beebb0c686bd32b3e0e80dae0e | 36 | 0 | 0 | 0 | 0 | 0 | f__Nocardioidaceae |
| 89a8bdd5818dc76514c0696b8e2de257 | 0 | 0 | 0 | 36 | 0 | 0 | f__Xanthomonadaceae |
| 058ac5f94b8e9961d60f819cfdbafac0 | 0 | 36 | 0 | 0 | 0 | 0 | f__uncultured |
| 7cb236e86347af3e46b519c95e41b124 | 0 | 36 | 0 | 0 | 0 | 0 | f__Nannocystaceae |
| db51fc4976e3e94aa6ea7dc40fb62f3a | 0 | 36 | 0 | 0 | 0 | 0 | f__Opitutaceae |
| 170caf383789ab5640fee8c0b5e516f9 | 0 | 0 | 0 | 0 | 0 | 35 |  |
| b632afe835fe2cf13ea433ba9798bbec | 0 | 0 | 35 | 0 | 0 | 0 | f__uncultured |
| 774aee1a9dcd409268879da3e2377984 | 0 | 0 | 35 | 0 | 0 | 0 | f__Bacillaceae |
| f77fc3695c851de86f7bf796dbdf46ef | 0 | 0 | 35 | 0 | 0 | 0 | f__Nocardioidaceae |
| 0b03d68b75c46f3a82614a0469c016b4 | 35 | 0 | 0 | 0 | 0 | 0 | f__Chthoniobacteraceae |
| aca56c04e959495a1bddfb22841fd474 | 35 | 0 | 0 | 0 | 0 | 0 | f__Peptostreptococcaceae |
| 6fc4344edd5af4ff589ca5479512ef98 | 0 | 0 | 0 | 0 | 35 | 0 | f__Xanthomonadaceae |
| eb2e7db9525da686e72004fc0164a66f | 0 | 0 | 34 | 0 | 0 | 0 | f__Solirubrobacteraceae |
| 265bb8f30a62f9239a39be5eed031404 | 34 | 0 | 0 | 0 | 0 | 0 | f__A4b |
| c9e939ff9dda4df762a0244439655424 | 34 | 0 | 0 | 0 | 0 | 0 | f__Chitinophagaceae |
| b04f6e1e206a0b97ba4feec002036275 | 0 | 0 | 0 | 0 | 34 | 0 | f__Beijerinckiaceae |
| 2c5c16380592aceb44b5e291195b67a3 | 0 | 0 | 0 | 0 | 34 | 0 | f__Hyphomicrobiaceae |
| acb2053362351a35e905866dd7c99f02 | 0 | 0 | 0 | 34 | 0 | 0 | f__Bacillaceae |
| 1090f60c754803879cd1df137ff19e76 | 0 | 0 | 0 | 34 | 0 | 0 | f__Xanthomonadaceae |
| 8dd731d5b74745678feb5b86a63e82a3 | 0 | 34 | 0 | 0 | 0 | 0 | f__Bacillaceae |
| b1b3e0fc7669e51277d57b143d3d3c02 | 0 | 34 | 0 | 0 | 0 | 0 | f__Sphingomonadaceae |
| 24cfe1660f834297e06578c15f588ab9 | 0 | 0 | 0 | 0 | 0 | 33 | f__Microscillaceae |
| 034dfdc73e9c0b0edc318a327c8bceb2 | 33 | 0 | 0 | 0 | 0 | 0 | f__Microscillaceae |
| 543a11f78a2f72ed1fcc8d0890ba8ac6 | 0 | 0 | 0 | 0 | 33 | 0 | f__Sphingomonadaceae |
| dd7739455eff662ce752fb80702ef215 | 0 | 0 | 0 | 0 | 33 | 0 | f__Azospirillaceae |
| 34a9f6b7115bb84bf00bcfe5c2e0b7c4 | 0 | 0 | 0 | 33 | 0 | 0 | f__Opitutaceae |
| 6eee36e8f353332a1e42044b105ddf72 | 0 | 0 | 0 | 33 | 0 | 0 | f__Rhodobacteraceae |
| 49e83ff55ff07a1944e6211476fdb517 | 0 | 0 | 0 | 33 | 0 | 0 | f__Sphingomonadaceae |
| e2626cf325151b8d2f22dece42f78dc9 | 0 | 0 | 0 | 33 | 0 | 0 | f__JG30-KF-CM45 |
| 755efc4dbe2514637e77a75400c17809 | 32 | 0 | 0 | 0 | 0 | 0 | f__Alicyclobacillaceae |
| 5c160e1019c3a98ed9b97ee361895a61 | 0 | 0 | 0 | 0 | 32 | 0 | f__Bacillaceae |
| 8fa382c6f5deb5a0c1f8f2c68dec4ac2 | 0 | 32 | 0 | 0 | 0 | 0 | f__Unknown_Family |
| 1de256e9c3573c60e6f5a0983152d562 | 0 | 0 | 0 | 0 | 0 | 31 | f__Nocardioidaceae |
| 08d1893ac3c7d25d9ffd2133d15a3790 | 0 | 0 | 0 | 0 | 0 | 31 | f__Pseudomonadaceae |
| 21816d04512cf74158fa9970e1d3df25 | 0 | 0 | 0 | 0 | 0 | 31 | f__Comamonadaceae |
| bce37c7518e1ff8276ed3fd44a787004 | 0 | 0 | 31 | 0 | 0 | 0 | f__Bacillaceae |
| 46e1a82e908d7499e2341b41b71e7bf1 | 31 | 0 | 0 | 0 | 0 | 0 | f__Vicinamibacteraceae |
| b1e8beaa3165b55ccc6ac019bccdb8a9 | 31 | 0 | 0 | 0 | 0 | 0 | f__Microscillaceae |
| 559c9d474c39ea16b9e4a187ef5eac21 | 31 | 0 | 0 | 0 | 0 | 0 | f__Rhodanobacteraceae |
| 8b93583f18d59b67ef6a986f9019a26d | 0 | 0 | 0 | 0 | 31 | 0 | f__uncultured |
| 1c83d8bce17796730e8704a305bbd891 | 0 | 0 | 0 | 0 | 31 | 0 | f__Rhizobiaceae |
| 5bf35e8f563416a304847154f1a806cc | 0 | 0 | 0 | 0 | 31 | 0 | f__A4b |
| f68da4197f91d789c82fde6c954df425 | 0 | 0 | 0 | 31 | 0 | 0 | f__Saccharimonadales |
| 0e1fa9096fc083611be72d8482aa24c3 | 0 | 0 | 0 | 31 | 0 | 0 | f__AKYG1722 |
| 641e3a63e57e31d61c6d0cf4b2e4440b | 0 | 14 | 0 | 17 | 0 | 0 | f__Flavobacteriaceae |
| 23127bdfd7b76580805c24545c5a3b77 | 0 | 0 | 30 | 0 | 0 | 0 | f__Devosiaceae |
| 3af1b88700b3da7372e9e02541d0d7d0 | 0 | 0 | 30 | 0 | 0 | 0 | f__Pirellulaceae |
| f4b7a305ddeca705102c5e35f89dfac4 | 0 | 0 | 0 | 0 | 30 | 0 | f__Rubritaleaceae |
| 2323465fcacd86faa676ac45244b67cc | 0 | 0 | 0 | 0 | 30 | 0 | f__Xanthomonadaceae |
| 99041f5d90d86e8559eb97416b414f79 | 0 | 30 | 0 | 0 | 0 | 0 | f__Nocardioidaceae |
| 1268edecb82c2ef1cb680f19d8e4e1f7 | 0 | 30 | 0 | 0 | 0 | 0 | f__Caldicoprobacteraceae |
| c79557c032e5a24b7fa53b3c493899e7 | 0 | 30 | 0 | 0 | 0 | 0 | f__Sphingomonadaceae |
| ac8d95ca086ff7940660678863e97172 | 0 | 30 | 0 | 0 | 0 | 0 | f__Caulobacteraceae |
| 924fcac7008ef08f89986b14715d1e75 | 0 | 30 | 0 | 0 | 0 | 0 | f__BIrii41 |
| 5181dd6d8354a07d1cd18101baf44d37 | 0 | 30 | 0 | 0 | 0 | 0 | f__Sphingomonadaceae |
| 82a0fcad6c8f781b816eb4b561985d7b | 0 | 0 | 0 | 0 | 0 | 29 | f__Nocardioidaceae |
| 62b24d99d6655d8840d09da639ced042 | 0 | 0 | 0 | 0 | 0 | 29 | f__0319-7L14 |
| 52e4678c76d2e016dfc7372e07889811 | 0 | 0 | 29 | 0 | 0 | 0 | f__Xanthomonadaceae |
| e7837779dab545e3ee2169fb54bc3335 | 29 | 0 | 0 | 0 | 0 | 0 | f__Steroidobacteraceae |
| 6bd0b6bb01899a839510712b79584b7d | 29 | 0 | 0 | 0 | 0 | 0 | f__Microscillaceae |
| 24b90cee5e9b7af86bada395225afce7 | 29 | 0 | 0 | 0 | 0 | 0 | f__Sphingomonadaceae |
| 46c2eafc9dcf9bdd115fca6a5be09d20 | 0 | 0 | 0 | 0 | 29 | 0 | f__Xanthomonadaceae |
| 1b5d14c5063ea10e442b1670d1798f0c | 0 | 0 | 0 | 29 | 0 | 0 | f__Myxococcaceae |
| 491ead851fd8a9f72b359b90a24bd4d2 | 0 | 0 | 0 | 29 | 0 | 0 | f__Nocardiaceae |
| e4d4f08edf396a75876ad6a9f3c9755b | 0 | 29 | 0 | 0 | 0 | 0 | f__Caulobacteraceae |
| 0134bcba4f5411c7a3c86c097d6598d9 | 0 | 0 | 28 | 0 | 0 | 0 | f__Cellvibrionaceae |
| 73f670089aa9d6e14b5613a397bb67ab | 0 | 0 | 28 | 0 | 0 | 0 | f__Planococcaceae |
| 31a62a20f8841a81874ed29d49d4f529 | 0 | 28 | 0 | 0 | 0 | 0 | f__Pedosphaeraceae |
| 2def7f461f4234b2d503a4cc44d3ee08 | 0 | 0 | 0 | 0 | 0 | 27 | f__Rubritaleaceae |
| 66a62b47da8042a54d3b05eb8c01e800 | 0 | 0 | 0 | 0 | 0 | 27 | f__Myxococcaceae |
| 847566bbbb0a908f259f31e1eb79b8df | 0 | 0 | 0 | 0 | 0 | 27 | f__MB-A2-108 |
| 610b667f9ce60e516076fd17e4effbb3 | 27 | 0 | 0 | 0 | 0 | 0 | f__Xanthomonadaceae |
| e21dd49b4b82449e59d6a8fe919dea76 | 0 | 0 | 0 | 0 | 27 | 0 | f__Nocardioidaceae |
| e8b703a3de294d1864caf0fa0146a6fd | 0 | 27 | 0 | 0 | 0 | 0 | f__Pirellulaceae |
| 7f36a6cc5990426dad8eaf7fe6385c22 | 0 | 0 | 0 | 0 | 0 | 26 | f__Planococcaceae |
| bcea5501fcdfd3802ab2c9e5baa01eb7 | 0 | 0 | 0 | 0 | 0 | 26 | f__Cytophagaceae |
| 6dbc1dd1e68d34eb7eb28aabad5411dc | 0 | 0 | 26 | 0 | 0 | 0 | f__Mycobacteriaceae |
| 7fc345c0158169106bfc1a0ffa1b9c73 | 0 | 0 | 0 | 0 | 26 | 0 | f__Nocardioidaceae |
| 4448f112d492e013511502369f1413f1 | 0 | 0 | 0 | 26 | 0 | 0 | f__Alcaligenaceae |
| dcab2b04386c6da827709bc763615323 | 0 | 26 | 0 | 0 | 0 | 0 | f__Devosiaceae |
| 6f211326358e32ca9544ac066d9a8045 | 0 | 26 | 0 | 0 | 0 | 0 | f__Bacillaceae |
| 20ff930548d9a9cfc5281994d6901c33 | 0 | 26 | 0 | 0 | 0 | 0 | f__Rhodobacteraceae |
| 7d7694941b762230f3e33b007385c9a7 | 0 | 26 | 0 | 0 | 0 | 0 | f__Bacillaceae |
| 2a17244bb5bdeefa6364dfb29877f17f | 0 | 26 | 0 | 0 | 0 | 0 | f__Sandaracinaceae |
| 3dd06d1624de65528644e48e8f645cdb | 0 | 0 | 0 | 0 | 0 | 25 | f__uncultured |
| 4e9b1333524d5c49fd28b3413ce34316 | 0 | 0 | 0 | 0 | 0 | 25 | f__Myxococcaceae |
| 94b3df234d04155d10de52611516105d | 0 | 0 | 0 | 0 | 0 | 25 | f__Solibacteraceae |
| 829d4770bd0394ef2cb7e98acb47acd9 | 0 | 0 | 25 | 0 | 0 | 0 | f__Spirosomaceae |
| c336bd6fe2a43819bdb2608a5b5b2cec | 25 | 0 | 0 | 0 | 0 | 0 | f__Microscillaceae |
| 9562893e53bb65158bdc529582652ac0 | 25 | 0 | 0 | 0 | 0 | 0 | f__Rhodanobacteraceae |
| a9d08b2a939918750c52bec0f47b70fe | 0 | 0 | 0 | 0 | 25 | 0 | f__WD2101_soil_group |
| bdfef8c6ab3509b74c3460e498d65943 | 0 | 0 | 0 | 0 | 25 | 0 | f__67-14 |
| 3896a18e365af936d25fbbadf18841c6 | 0 | 0 | 0 | 25 | 0 | 0 | f__Verrucomicrobiaceae |
| ac7507ef36d389ed090d24725b1b163d | 0 | 0 | 0 | 25 | 0 | 0 | f__Chitinophagaceae |
| 8023e69a1ccc961175d332b4f9fa4708 | 0 | 0 | 0 | 25 | 0 | 0 | f__LWQ8 |
| c886f2afcbbd14250b7338bce771e2de | 0 | 25 | 0 | 0 | 0 | 0 | f__Rhodomicrobiaceae |
| 790b9949db8e07ea9a217703df7bc4fe | 0 | 0 | 0 | 0 | 0 | 24 |  |
| a0e85027f8b357ee5d79fa507a02210a | 0 | 0 | 0 | 0 | 0 | 24 | f__Pirellulaceae |
| 427f47e24def85712bb922f6e82a1fa1 | 0 | 0 | 0 | 0 | 0 | 24 | f__Thermoanaerobaculaceae |
| 0aecdd6f98633745571525a08df22bf7 | 0 | 0 | 0 | 0 | 0 | 24 | f__AKYG1722 |
| dff01aa63991995b4c1f2e25292c57c9 | 0 | 0 | 24 | 0 | 0 | 0 | f__Defluviitaleaceae |
| 2323ff19cecafd863323a38740113668 | 0 | 0 | 24 | 0 | 0 | 0 | f__AKYG1722 |
| b0970fe24d0bebbef19ac4f4e4c228d8 | 0 | 0 | 24 | 0 | 0 | 0 | f__Parachlamydiaceae |
| 34a2705f400c2c8d005840544cc9c210 | 0 | 0 | 24 | 0 | 0 | 0 | f__Chitinophagaceae |
| 76408052f8a04b57e4dc368560a79d3d | 24 | 0 | 0 | 0 | 0 | 0 | f__Paenibacillaceae |
| 1621223b336c4807be9d68a70e230049 | 24 | 0 | 0 | 0 | 0 | 0 | f__Parachlamydiaceae |
| b3544bba9977a858f70fc6abfa9826c3 | 24 | 0 | 0 | 0 | 0 | 0 | f__Pirellulaceae |
| 68503a51bc6d9dd22d7db16e68241526 | 24 | 0 | 0 | 0 | 0 | 0 | f__Solirubrobacteraceae |
| 5434f86c7eb1a2640de61caf3a084234 | 0 | 0 | 0 | 0 | 24 | 0 | f__Xanthomonadaceae |
| 9f604fdda3a97f8acb9b789d7cdea9ec | 0 | 0 | 0 | 0 | 24 | 0 | f__Pseudomonadaceae |
| 587e5b7994592ccaecd44753ee6691a5 | 0 | 0 | 0 | 0 | 24 | 0 | f__Micrococcaceae |
| 46260265fb48a9c61904e45cf2b2e7b2 | 0 | 0 | 0 | 0 | 24 | 0 | f__Euzebyaceae |
| cc9a6d389df4a5f519b1c34ac76e8e81 | 0 | 0 | 0 | 0 | 24 | 0 | f__AKYG1722 |
| bb1b32192f7bdcf361b97cf603e8c85e | 0 | 0 | 0 | 24 | 0 | 0 | f__01D2Z36 |
| e76944647d1ea7ce7826f3ff8956aa70 | 0 | 0 | 0 | 24 | 0 | 0 | f__Rhodanobacteraceae |
| 5fc69cc2919de3001b06518eb7ca8ff3 | 0 | 24 | 0 | 0 | 0 | 0 | f__Microbulbiferaceae |
| bde3f6102358e264e61c6864e2aa0a9a | 0 | 24 | 0 | 0 | 0 | 0 | f__Bacillaceae |
| 06ee736d515a0251d1be42669265d85b | 0 | 0 | 0 | 0 | 0 | 23 | f__Reyranellaceae |
| e6085f501f8377e4fdad58404346d070 | 0 | 0 | 0 | 0 | 0 | 23 | f__Nocardioidaceae |
| 65d18a46c90011d9fcfc1aff637ce9c4 | 0 | 0 | 23 | 0 | 0 | 0 | f__Flavobacteriaceae |
| 27e6975adf1fb17b02386e3c1be90733 | 23 | 0 | 0 | 0 | 0 | 0 | f__Chitinophagaceae |
| fb634d0909278acb544d523ebcf4db89 | 0 | 0 | 0 | 0 | 23 | 0 | f__Sericytochromatia |
| 30bddedb66a118ac3121911289669cd0 | 0 | 0 | 0 | 23 | 0 | 0 | f__Blastocatellaceae |
| 397dc9b06e12ec4d21ad1ef638e66f65 | 0 | 0 | 0 | 23 | 0 | 0 | f__Spirosomaceae |
| 62409c5f703b30fb8aea2f63fd48646f | 0 | 23 | 0 | 0 | 0 | 0 | f__Pirellulaceae |
| a55ae1c48c673bc3e466cdb9ae039020 | 0 | 23 | 0 | 0 | 0 | 0 | f__Cytophagaceae |
| d4baf26d43d51fc82e6398f1c9161263 | 0 | 0 | 0 | 0 | 0 | 22 | f__Xanthomonadaceae |
| e72437102356def129a86230f0ac66b2 | 0 | 0 | 0 | 0 | 0 | 22 | f__Nitrospiraceae |
| 92d90afd41a26b72a91675496af776f9 | 0 | 0 | 22 | 0 | 0 | 0 | f__Rubinisphaeraceae |
| 4f8b33cba71b62998227b3370ee7e5ff | 0 | 0 | 0 | 0 | 22 | 0 | f__Hymenobacteraceae |
| 1180c1d797dcbd52f7273cfe2fbbf28a | 0 | 0 | 0 | 0 | 22 | 0 | f__Legionellaceae |
| d200f2bb50fe966f352a35e73bb0a947 | 0 | 0 | 0 | 0 | 22 | 0 | f__Rhizobiaceae |
| a05a5b64dd5784c6f52e27f3d4e26956 | 0 | 0 | 0 | 0 | 22 | 0 | f__Chitinophagaceae |
| a03bbc6e4bf43297cf251c93761f2fbc | 0 | 0 | 0 | 22 | 0 | 0 | f__0319-7L14 |
| 2451089bc93bb16103b18fff8f8a8419 | 0 | 22 | 0 | 0 | 0 | 0 | f__Bacillaceae |
| b2b09ee44d97bba051bd1cf3721d0c57 | 21 | 0 | 0 | 0 | 0 | 0 | f__Rhodanobacteraceae |
| 86d253a47ceb6163b16ad19420e51816 | 21 | 0 | 0 | 0 | 0 | 0 | f__Rhodanobacteraceae |
| a50e8a330efcc4649c11d77ed1879199 | 21 | 0 | 0 | 0 | 0 | 0 | f__SJA-28 |
| d693f9bba7393cda1f039262c77f8c8a | 0 | 0 | 0 | 0 | 21 | 0 | f__uncultured |
| e81c107a8e15c3ce93290f54388aa0f1 | 0 | 0 | 0 | 0 | 21 | 0 | f__Pirellulaceae |
| 2119d9b3793b88e0461c12c6acc47f8f | 0 | 21 | 0 | 0 | 0 | 0 | f__Spirosomaceae |
| 5fd7d1cdee63c03d7f4e15d73cd6122f | 0 | 0 | 0 | 0 | 0 | 20 | f__Vicinamibacteraceae |
| fdb47e07e2be193f994b5d664998f37f | 0 | 0 | 0 | 0 | 0 | 20 | f__Caldicoprobacteraceae |
| 3c8ed7edc98da6ea8d5e03b3ff56d52c | 0 | 0 | 0 | 0 | 0 | 20 | f__Pyrinomonadaceae |
| 25d650e01ee7406487f995cc005abcf2 | 20 | 0 | 0 | 0 | 0 | 0 | f__AKIW781 |
| b60aeaf5c7da85d5e960cc26ba82b914 | 20 | 0 | 0 | 0 | 0 | 0 | f__Terrimicrobiaceae |
| ee49f673f6f4f4acaad91c4847a1c5b9 | 0 | 0 | 0 | 0 | 20 | 0 | f__Xanthomonadaceae |
| dc999dc6ed84b55cb23c9180bd7a2f42 | 0 | 0 | 0 | 0 | 20 | 0 | f__Spirosomaceae |
| ff556263ff71cc2add4f8e1e9258f295 | 0 | 0 | 0 | 0 | 20 | 0 | f__Herpetosiphonaceae |
| a203fb05a05d925f83ba713f79d49b4a | 0 | 0 | 0 | 20 | 0 | 0 | f__Xanthomonadaceae |
| c0bbcdd1d710c9140e732cc6aac6ef1e | 0 | 20 | 0 | 0 | 0 | 0 | f__LWQ8 |
| 8359d4af19abed779829233c8bf68fcd | 0 | 0 | 0 | 0 | 0 | 19 | f__Burkholderiaceae |
| f7ece90a79c6595a9c91e2170760b1f9 | 0 | 0 | 19 | 0 | 0 | 0 | f__Xanthomonadaceae |
| 4b8cc1812b7f10be92e55b95d2cbd6b0 | 0 | 0 | 19 | 0 | 0 | 0 | f__KD3-93 |
| 246f0139bfc416ffda30ff2360964775 | 0 | 0 | 0 | 0 | 19 | 0 | f__Rubinisphaeraceae |
| 8fb07b4abdac176d698c05e20d46ee36 | 0 | 0 | 0 | 0 | 19 | 0 | f__Nocardioidaceae |
| 9425fc85500288a91c7bf398b5adb178 | 0 | 0 | 0 | 0 | 19 | 0 | f__Nocardioidaceae |
| 67774cf10498c6d09e1b166422f05f78 | 0 | 0 | 0 | 0 | 0 | 18 | f__Sphingobacteriaceae |
| 4bd9fed54c374ed8a8093577a8954273 | 0 | 0 | 18 | 0 | 0 | 0 | f__Solibacteraceae |
| df545a01b5dd5e46dc83744784306616 | 0 | 0 | 18 | 0 | 0 | 0 | f__Vicinamibacteraceae |
| e46b204dc7aa9755c1b772175874cc8f | 0 | 0 | 18 | 0 | 0 | 0 | f__Sandaracinaceae |
| a50eb30bb40407940dafa7edf3cd4928 | 18 | 0 | 0 | 0 | 0 | 0 | f__Xanthomonadaceae |
| ee7563698b07041ea57e4c17f81c6898 | 18 | 0 | 0 | 0 | 0 | 0 | f__Pirellulaceae |
| 3445b76d5dd7f1a12df78c13e479138a | 18 | 0 | 0 | 0 | 0 | 0 | f__Nannocystaceae |
| 0f9b4de595ccb42696dffa72979f06d1 | 0 | 0 | 0 | 0 | 18 | 0 | f__Pedosphaeraceae |
| 3f7a983c9c0beef7c6b2cbf768b51bb2 | 0 | 0 | 0 | 0 | 18 | 0 | f__EPR3968-O8a-Bc78 |
| 8936a3f9273a8b5b98d18c587dc41fb7 | 0 | 0 | 0 | 0 | 18 | 0 | f__Nocardiaceae |
| c54c83158d33abd9ecff8afef9e2e0a5 | 0 | 0 | 0 | 0 | 18 | 0 | f__Beijerinckiaceae |
| f84b77af824e7c5ab7b3612147b9e903 | 0 | 0 | 0 | 18 | 0 | 0 | f__Verrucomicrobiaceae |
| 3dcf81beeb354d338448420b2f001df6 | 0 | 0 | 0 | 18 | 0 | 0 | f__Cytophagaceae |
| 2945a2a6ef818028eec33a73aeee6a40 | 0 | 0 | 0 | 18 | 0 | 0 | f__Verrucomicrobiaceae |
| 332fe6563f0300c5fcba135371dabfac | 0 | 18 | 0 | 0 | 0 | 0 | f__Gemmatimonadaceae |
| e6039e98b945ef0bcf9fd6638efc3c47 | 0 | 18 | 0 | 0 | 0 | 0 | f__EPR3968-O8a-Bc78 |
| f013df3b9936e08468c30dc6f5035ce2 | 0 | 18 | 0 | 0 | 0 | 0 | f__bacteriap25 |
| 26a7f7c8ffbc4cd2ee8ffc6a74429f46 | 0 | 0 | 0 | 0 | 0 | 17 | f__MBA03 |
| 4f42410ac288cb3c61aeb996b9cc7c2a | 0 | 0 | 0 | 0 | 0 | 17 | f__Steroidobacteraceae |
| 010418f9d561452f9484801ec822cf04 | 0 | 0 | 0 | 0 | 0 | 17 | f__Spirosomaceae |
| cd927f0f8ae3a6510b8e77c3dce0b0c6 | 0 | 0 | 0 | 0 | 0 | 17 | f__Methyloligellaceae |
| 26d400e58473ceac6280e9dffd40f075 | 0 | 0 | 0 | 0 | 0 | 17 | f__Sphingomonadaceae |
| f204eb2f48f337cfcf72756971ae1775 | 0 | 0 | 17 | 0 | 0 | 0 | f__Nocardiopsaceae |
| 101f7b7479ebca22c0f286a481a64a1a | 0 | 0 | 17 | 0 | 0 | 0 | f__Flavobacteriaceae |
| c31ae0a05d514303fd922d7086a9c879 | 17 | 0 | 0 | 0 | 0 | 0 | f__Spirosomaceae |
| b1e73ab8bcaafebc13ff331f21be6386 | 0 | 0 | 0 | 0 | 17 | 0 | f__Geminicoccaceae |
| 52fd4822bdc4818c84859522d756f7ff | 0 | 0 | 0 | 17 | 0 | 0 | f__Alcaligenaceae |
| 105eac749ebb78e1c896f3b3f4ddc8c6 | 0 | 0 | 0 | 17 | 0 | 0 | f__Rubritaleaceae |
| 100ebe062087a18dfff8b79922252462 | 0 | 17 | 0 | 0 | 0 | 0 | f__Glycomycetaceae |
| edb3f486cc685635a52b8bd9323504db | 0 | 0 | 0 | 0 | 0 | 16 | f__Cyclobacteriaceae |
| e315cc5585a5a19786d94ac253228437 | 0 | 0 | 0 | 0 | 0 | 16 | f__Hymenobacteraceae |
| 55e039959b48b22f9b18a85498a37361 | 0 | 0 | 16 | 0 | 0 | 0 | f__Verrucomicrobiaceae |
| dcb3d5d3271e247e99bf00f995723b43 | 16 | 0 | 0 | 0 | 0 | 0 | f__Rubinisphaeraceae |
| e335b3cb6a0a7ec66a1e400493bc1b64 | 16 | 0 | 0 | 0 | 0 | 0 | f__Chitinophagaceae |
| e314c8618d24d8f6c1bab506c3ba5a1f | 0 | 0 | 0 | 0 | 16 | 0 | f__Pirellulaceae |
| 812a36601863be8b4bd517482121faf0 | 0 | 16 | 0 | 0 | 0 | 0 | f__Pseudohongiellaceae |
| 367f28a13f40e5b23c6d47d83251245c | 0 | 16 | 0 | 0 | 0 | 0 | f__Nitrospiraceae |
| 21e85b70562dc5b9a8b8d2c755b36ecf | 0 | 16 | 0 | 0 | 0 | 0 | f__Sedimentibacteraceae |
| 8efdf5a3efad6550d75f8b3bd213a3ac | 0 | 16 | 0 | 0 | 0 | 0 | f__Flavobacteriaceae |
| 35f4d014228747cf07bf9ab17dd2261a | 0 | 16 | 0 | 0 | 0 | 0 | f__Garciellaceae |
| 2e4366b720a1bdb8c9c9f3dd8565d227 | 0 | 0 | 0 | 0 | 0 | 15 | f__Sedimentibacteraceae |
| 0d169753f7ed6aa243a8156cab2205d3 | 0 | 0 | 15 | 0 | 0 | 0 | f__Flavobacteriaceae |
| b33c80424116e05b1db25f27012b2a78 | 0 | 0 | 15 | 0 | 0 | 0 | f__Chthoniobacteraceae |
| e2bdc59b3c20402b928af2b490b08e89 | 0 | 0 | 15 | 0 | 0 | 0 | f__Rhodobacteraceae |
| ff1d7231f3e174029688301e0d223822 | 15 | 0 | 0 | 0 | 0 | 0 | f__Microscillaceae |
| 270d58af18a822ef1e42ad4a88ec6b98 | 15 | 0 | 0 | 0 | 0 | 0 | f__Verrucomicrobiaceae |
| 7fd193ff4dfdccdd00382b9e2b303b27 | 15 | 0 | 0 | 0 | 0 | 0 | f__Solibacteraceae |
| 1b50a89f32973fce5f352fb5d02bbccc | 0 | 0 | 0 | 0 | 15 | 0 | f__Pirellulaceae |
| 5fc754b2dc6b257877cb486d06956b0e | 0 | 0 | 0 | 15 | 0 | 0 | f__KD3-93 |
| a7222e7811b9a472945ed684283ddcd8 | 0 | 0 | 0 | 15 | 0 | 0 | f__Xanthomonadaceae |
| bbd45d19bdc5136610f2514f29c02bc7 | 0 | 0 | 0 | 15 | 0 | 0 | f__A4b |
| 41a3cc11486cca0b5ad3f5430ff23d48 | 0 | 15 | 0 | 0 | 0 | 0 | f__Rhodanobacteraceae |
| ea253eb2d1beac7250007c4f92d87be4 | 0 | 15 | 0 | 0 | 0 | 0 | f__Pseudomonadaceae |
| 880e718d096c25b9f82386278a2a2bc9 | 0 | 0 | 0 | 0 | 0 | 14 | f__Lachnospiraceae |
| 1927b5d9e71898b51b11ac2c3e29c4c7 | 0 | 0 | 0 | 0 | 0 | 14 | f__Blastocatellaceae |
| 07e3ca0f87ea6586674fe3e98b137685 | 0 | 0 | 14 | 0 | 0 | 0 | f__Xanthomonadaceae |
| 332bf2747e2b511260b9d3563e238d57 | 0 | 0 | 14 | 0 | 0 | 0 | f__PLTA13 |
| b3f10179e4170c7af41b191e2667eeb6 | 0 | 0 | 0 | 14 | 0 | 0 | f__Xanthomonadaceae |
| cf16c715abc409cad2c34bab2359182a | 0 | 14 | 0 | 0 | 0 | 0 | f__Xanthomonadaceae |
| 40eccc8db51de1d4570fd50a02cff756 | 0 | 14 | 0 | 0 | 0 | 0 | f__Xanthomonadaceae |
| 8d9f392d4b6c76c2150f55bbf4b01c34 | 0 | 14 | 0 | 0 | 0 | 0 | f__Sphingobacteriaceae |
| 1283856ec58d752e331d2bad375ded28 | 0 | 14 | 0 | 0 | 0 | 0 | f__Limnochordia |
| 9dce8a04a834ff36ce9377f523863a40 | 0 | 0 | 0 | 0 | 0 | 13 | f__Bacillaceae |
| 8c4c9fe80284ea37fe9aa12c4559ef60 | 0 | 0 | 0 | 0 | 0 | 13 | f__Sphingobacteriaceae |
| 88fb9d774927d0cc80b6e975e1b71b0a | 0 | 0 | 13 | 0 | 0 | 0 | f__Sandaracinaceae |
| 4ba984bffd49e433c61b0bc6a986b6a1 | 13 | 0 | 0 | 0 | 0 | 0 | f__Xiphinematobacteraceae |
| f1a3a5f386cf831c4c048ef321f2bfe0 | 13 | 0 | 0 | 0 | 0 | 0 | f__Cytophagaceae |
| 0e4597d8abcc317f3964e18978dce264 | 0 | 0 | 0 | 0 | 13 | 0 | f__Iamiaceae |
| 066c7ee45449a4dbd8ff34a2055937ce | 0 | 0 | 0 | 13 | 0 | 0 | f__Chitinophagaceae |
| 9be49d92463136ff5ce9cfdc134fac8e | 0 | 13 | 0 | 0 | 0 | 0 | f__Bacillaceae |
| 772ca3e860a057d6a32032d904c42923 | 0 | 13 | 0 | 0 | 0 | 0 | f__Pseudomonadaceae |
| 9bac1d16797047c9325ee00fde4c1bb1 | 0 | 13 | 0 | 0 | 0 | 0 | f__uncultured |
| dc2a85ee8dadfe82d826f98eca70d5c7 | 0 | 13 | 0 | 0 | 0 | 0 | f__Pseudomonadaceae |
| fb31a154780fbea0cb713a61ec49c69c | 0 | 0 | 12 | 0 | 0 | 0 | f__Chitinophagaceae |
| 31248b502232034ee44a44e84444e8af | 0 | 0 | 12 | 0 | 0 | 0 | f__Pseudomonadaceae |
| 6897fb6f4f84aed3631c3e4a020809a8 | 0 | 0 | 0 | 0 | 12 | 0 | f__Parachlamydiaceae |
| 41dba7d9a4a841cfdf1412af0a5512c9 | 0 | 0 | 0 | 12 | 0 | 0 | f__Solirubrobacteraceae |
| 92e671610cf47d461d679c1d3b8a34de | 0 | 0 | 0 | 12 | 0 | 0 | f__Trueperaceae |
| ca191acfe0e8f6bb06353a64760a30bd | 0 | 0 | 0 | 12 | 0 | 0 | f__Phycisphaeraceae |
| e2775983ba4a1d2785703c5d6acde812 | 0 | 12 | 0 | 0 | 0 | 0 | f__Hungateiclostridiaceae |
| 74a45d346138602e5a22a50e25b73d30 | 0 | 12 | 0 | 0 | 0 | 0 | f__uncultured |
| 4d7a26b612c3d138f213cb3eec1cd060 | 0 | 12 | 0 | 0 | 0 | 0 | f__Nocardioidaceae |
| 20eb988269bdf0bece3242710d8d1c68 | 0 | 12 | 0 | 0 | 0 | 0 | f__Haloplasmataceae |
| a1e02f14d9fcbd9ececcfd385d0a55aa | 0 | 12 | 0 | 0 | 0 | 0 | f__Sphingobacteriaceae |
| db026d48b2d0049256cd127189afe448 | 11 | 0 | 0 | 0 | 0 | 0 | f__Inquilinaceae |
| 84601cce08c450864e0f66158d6f8363 | 11 | 0 | 0 | 0 | 0 | 0 | f__PLTA13 |
| 1d6d4ad82c33e9ac24aa5057d927b352 | 11 | 0 | 0 | 0 | 0 | 0 | f__Rhodobacteraceae |
| f1f1eff75df909793509a050d21cce3f | 11 | 0 | 0 | 0 | 0 | 0 | f__Verrucomicrobiaceae |
| 167281d72c4b4400e8cf497f18fe572f | 0 | 0 | 0 | 0 | 11 | 0 | f__Diplorickettsiaceae |
| f45339b9d4b7c48cf1b62d2fbcc81759 | 0 | 11 | 0 | 0 | 0 | 0 | f__Peptococcaceae |
| b006af854d60e5bd6d0db786b1197206 | 0 | 11 | 0 | 0 | 0 | 0 | f__Flavobacteriaceae |
| e0ebbf657796854ca7758213025ac8f5 | 0 | 11 | 0 | 0 | 0 | 0 | f__Rhodomicrobiaceae |
| 7e3509500cb10548fc693b5b924d8be4 | 0 | 0 | 0 | 0 | 0 | 10 | f__Chthoniobacteraceae |
| c9c363a2a6adad464df01a39cbd5e23b | 0 | 0 | 10 | 0 | 0 | 0 | f__Flavobacteriaceae |
| b7480aa98cc5e02b1a174a512004e353 | 0 | 0 | 10 | 0 | 0 | 0 | f__Flavobacteriaceae |
| 4a04fd94e9565ec405142adb27447dc7 | 0 | 0 | 10 | 0 | 0 | 0 | f__Bdellovibrionaceae |
| ace004bd4f7fd5d3ff8eac79c1296dfe | 0 | 0 | 10 | 0 | 0 | 0 | f__Flavobacteriaceae |
| 03b62b009d478c7b1455516f77031d70 | 0 | 0 | 10 | 0 | 0 | 0 | f__A4b |
| dfd119603812fb281d55da5d36505c2d | 0 | 0 | 10 | 0 | 0 | 0 | f__Flavobacteriaceae |
| 30d29e6b524a8df4d7c5a6aa80923f93 | 0 | 0 | 0 | 0 | 10 | 0 | f__Oscillospiraceae |
| a56fbe518e17fd3751ef0b25c341d608 | 0 | 0 | 0 | 0 | 10 | 0 | f__Nocardioidaceae |
| 7bcff0a88e20d3f6ff78dbdfad721c49 | 0 | 0 | 0 | 0 | 10 | 0 | f__Xanthomonadaceae |
| f537a8d618de01591bee605e876c4e94 | 0 | 10 | 0 | 0 | 0 | 0 | f__Chitinophagaceae |
| 242b8cd643c752915a844fefcc32f5c7 | 0 | 10 | 0 | 0 | 0 | 0 | f__MB-A2-108 |
| 56805ee4ecb80dec9676c3d3c1bf2616 | 0 | 10 | 0 | 0 | 0 | 0 | f__S32 |
| e99274ddf9a7df3475fcfc66f7a7ce2c | 0 | 10 | 0 | 0 | 0 | 0 | f__Planococcaceae |
| ec227142a0e52b0e0f49fc85e2e52aa1 | 0 | 0 | 0 | 0 | 0 | 9 | f__Chitinophagaceae |
| 0d8655a795da36529e66c9dab096c0d1 | 0 | 0 | 9 | 0 | 0 | 0 | f__Pirellulaceae |
| 97a5bc3bc1ae318bb32767f551da581f | 9 | 0 | 0 | 0 | 0 | 0 | f__Xanthomonadaceae |
| 1522b1184cffce9b32216718df11235b | 9 | 0 | 0 | 0 | 0 | 0 | f__Burkholderiaceae |
| 38eb3e0dc549a6885b444c7e787552f4 | 0 | 0 | 0 | 0 | 9 | 0 | f__Nitrospiraceae |
| 7f395137f5d20ba75381c0c34e9bb9ea | 0 | 0 | 0 | 0 | 9 | 0 | f__Saccharimonadales |
| b6c21a68f3cbd2902d84c0f74f286d6d | 0 | 0 | 0 | 0 | 9 | 0 | f__Acidiferrobacteraceae |
| bdff6cc635ef34f0da5fe791c14c6847 | 0 | 0 | 0 | 0 | 9 | 0 | f__Nocardioidaceae |
| f5ae2ede5dbbf23fd0b6c12893752e0a | 0 | 0 | 0 | 0 | 9 | 0 | f__Pseudonocardiaceae |
| 26da850eb5152c88958b208a7761457e | 0 | 0 | 0 | 9 | 0 | 0 | f__Iamiaceae |
| c181dd76340dbf2d2e699aa09d5f0f39 | 0 | 9 | 0 | 0 | 0 | 0 | f__Legionellaceae |
| ba30214498972e29794c0f270e175683 | 0 | 9 | 0 | 0 | 0 | 0 | f__Xanthomonadaceae |
| 5f230246559c73ad08aa418567235286 | 0 | 9 | 0 | 0 | 0 | 0 | f__Candidatus_Kaiserbacteria |
| 8cf7a4868571bc8b6911fdd3099ad29e | 0 | 9 | 0 | 0 | 0 | 0 | f__uncultured |
| 6c4a8c0a402494b57fb8e50cb5cade1c | 0 | 9 | 0 | 0 | 0 | 0 | f__0319-7L14 |
| 7f1e188e74cf8cf746d2cfc3d965c6e5 | 0 | 0 | 0 | 0 | 0 | 8 | f__Cellvibrionaceae |
| e70a2fc43ca0f9bbd1308b97e20bfdf6 | 0 | 0 | 0 | 0 | 0 | 8 | f__Erysipelotrichaceae |
| a961ff9d56e4915e56b960bc9f84a52e | 0 | 0 | 8 | 0 | 0 | 0 | f__Kapabacteriales |
| d48d978f6db7a6502009ad9ff87ffe39 | 0 | 0 | 8 | 0 | 0 | 0 | f__Sericytochromatia |
| a974be239bcb6bc02833d70bea04f5f6 | 0 | 0 | 8 | 0 | 0 | 0 | f__Flavobacteriaceae |
| 7ac7fca1f53435609e052db0b088ea89 | 0 | 0 | 8 | 0 | 0 | 0 | f__Acetobacteraceae |
| d20bd97441fde75b23688751861e92f6 | 0 | 0 | 0 | 0 | 8 | 0 | f__Micrococcaceae |
| 545eece67c3822f733405fdde9e84273 | 0 | 0 | 0 | 0 | 8 | 0 | f__0319-6G20 |
| e6341748e9776189cfa076e256c08235 | 0 | 0 | 0 | 0 | 8 | 0 | f__Cyclobacteriaceae |
| d21b9a5373cda4c6d19ef5161fef42d0 | 0 | 0 | 0 | 8 | 0 | 0 | f__Cyclobacteriaceae |
| 65083ca473fe51e7ab6f99069f25971c | 0 | 0 | 0 | 8 | 0 | 0 | f__Pseudomonadaceae |
| c50577f4dd590e7e8e62471221408659 | 0 | 8 | 0 | 0 | 0 | 0 | f__Xanthomonadaceae |
| 0a57a2658470ce821a59929d22d5dd9d | 7 | 0 | 0 | 0 | 0 | 0 | f__WD2101_soil_group |
| 2b0b2e42a4ec8f4c2650f0c79e527b69 | 7 | 0 | 0 | 0 | 0 | 0 | f__Devosiaceae |
| 10f9adc57789aaa9c417eb054cb3a5ee | 7 | 0 | 0 | 0 | 0 | 0 | f__Caldilineaceae |
| 47bf9fd2dfa660cd84af1d014cc15513 | 0 | 0 | 0 | 0 | 7 | 0 | f__Micrococcaceae |
| 702a3b9afea20104a03a7dc12b12a682 | 0 | 0 | 0 | 0 | 7 | 0 | f__Bdellovibrionaceae |
| cc88b71dec580d84105ee4cbbb8ddb87 | 0 | 0 | 0 | 0 | 7 | 0 | f__Xiphinematobacteraceae |
| fc47c24cdb970246c8f84157b847804f | 0 | 0 | 0 | 0 | 7 | 0 | f__Saprospiraceae |
| fed1166cf97fbab8e2047a0fa96368be | 0 | 7 | 0 | 0 | 0 | 0 | f__A4b |
| 4ea0b093afe2815a4ecce54966ef93e8 | 0 | 7 | 0 | 0 | 0 | 0 | f__Peptostreptococcales-Tissierellales |
| 4ec75405869c1ff38b7b084000e552c3 | 0 | 7 | 0 | 0 | 0 | 0 | f__Terrimicrobiaceae |
| 2f3217712516e4319523f65812f6b61f | 0 | 7 | 0 | 0 | 0 | 0 | f__Parachlamydiaceae |
| 8ff03db8dac75f0e66da91be47c89b27 | 0 | 0 | 6 | 0 | 0 | 0 | f__uncultured |
| 5c4549d0f511199b40a0a1cd0886dc7b | 0 | 0 | 6 | 0 | 0 | 0 | f__Flavobacteriaceae |
| dab2d95018b2ea62d6cded58921f6f47 | 0 | 0 | 0 | 0 | 6 | 0 | f__Cellvibrionaceae |
| d6b4b23177eca05fcc2f65105112384d | 0 | 0 | 0 | 0 | 6 | 0 | f__uncultured |
| 0f66dcceac4de370aee94ca38431ea68 | 0 | 0 | 0 | 6 | 0 | 0 | f__Pirellulaceae |
| bfddf89109fe3bbe62e49ea81fe91560 | 0 | 6 | 0 | 0 | 0 | 0 | f__Promicromonosporaceae |
| 67f5deb3163fb95e932a6e74c0fb8d41 | 0 | 6 | 0 | 0 | 0 | 0 | f__WD2101_soil_group |
| 68531a07cc38889cc93403b232d3469c | 0 | 0 | 0 | 0 | 0 | 5 | f__Sericytochromatia |
| 79ebeef961207acca29ea7dafbc535f2 | 0 | 0 | 0 | 0 | 0 | 5 | f__Enterobacteriaceae |
| 9ad3e41ba2ebb60837a243236fe8faf4 | 0 | 0 | 5 | 0 | 0 | 0 | f__Cyclobacteriaceae |
| 95d8a0e59c8940dde7841a76a39402d9 | 0 | 0 | 5 | 0 | 0 | 0 | f__Chloroplast |
| fe5262d5c4711a6d4cace2b0c98bab9b | 0 | 0 | 5 | 0 | 0 | 0 | f__uncultured |
| 39ba97d6338f97869200054596804f7e | 0 | 0 | 5 | 0 | 0 | 0 | f__Chitinophagaceae |
| 4330c7a0d941d28ac5f82f0dfa31f52b | 5 | 0 | 0 | 0 | 0 | 0 | f__R7C24 |
| 93944d8e9acf05268a7b7e20cfb555a9 | 0 | 0 | 0 | 5 | 0 | 0 | f__Inquilinaceae |
| 87619634a625cb8dbc1684289676962a | 0 | 0 | 0 | 5 | 0 | 0 | f__Planococcaceae |
| 612e69b616dab3b98fcd21f1d2b41a83 | 0 | 5 | 0 | 0 | 0 | 0 | f__Weeksellaceae |
| fd6d3392205c4d9a56ca2dd24933f90a | 0 | 5 | 0 | 0 | 0 | 0 | f__Pirellulaceae |
| 96ff135116e07eb091d07cea82f31bf4 | 0 | 5 | 0 | 0 | 0 | 0 | f__UA11 |
| 567da236a3800f5350b444fb178c4442 | 0 | 5 | 0 | 0 | 0 | 0 | f__Xanthomonadaceae |
| 8cdbf6f30a70f7f37a21152729f9e5ca | 0 | 5 | 0 | 0 | 0 | 0 | f__Chitinophagaceae |
| dad60521f5a6e8cbbf9c5c2255c3df00 | 0 | 5 | 0 | 0 | 0 | 0 | f__Xanthomonadaceae |
| 34133feebe9627bf24166a547d04abd4 | 0 | 0 | 4 | 0 | 0 | 0 |  |
| 8876ec14dfb694f3420985d7dedc4b03 | 0 | 0 | 4 | 0 | 0 | 0 | f__Rhizobiaceae |
| 87d0c44ef4943b558c5bb48fe27add4c | 0 | 0 | 4 | 0 | 0 | 0 | f__Streptomycetaceae |
| 21774ca9644fd82ac77f12ba0dab4c86 | 0 | 0 | 4 | 0 | 0 | 0 | f__OLB14 |
| 5f8752377227132dafc04c86d7d9725c | 0 | 0 | 4 | 0 | 0 | 0 | f__uncultured |
| 7d3aa99236b0f876b4efa547e1bbe324 | 0 | 0 | 4 | 0 | 0 | 0 | f__Anaerolineaceae |
| 9cc0e4b57b072329dc79a54433120c07 | 4 | 0 | 0 | 0 | 0 | 0 | f__Ilumatobacteraceae |
| 99c62ce1b1cf949b5939e6e303ab78d3 | 0 | 0 | 0 | 0 | 4 | 0 | f__Thermoanaerobaculaceae |
| 40fa4d4fd69978e3b575d879736f841c | 0 | 0 | 0 | 4 | 0 | 0 | f__Chitinophagaceae |
| 8a1768a584146e027df8593de79f05d5 | 0 | 0 | 0 | 0 | 0 | 3 | f__Rhizobiaceae |
| 6ab0f56b6a3669425229eedea73e5f02 | 0 | 0 | 0 | 0 | 0 | 3 | f__Myxococcaceae |
| 43498ab84846397cd6d44ac401f9e3e3 | 0 | 0 | 3 | 0 | 0 | 0 | f__Micrococcaceae |
| 7907fafda2631a812561872bb193a433 | 0 | 0 | 3 | 0 | 0 | 0 | f__MWH-CFBk5 |
| a3bc7f9c94bb6adc06450a7bfe3f1842 | 0 | 0 | 3 | 0 | 0 | 0 | f__Unknown_Family |
| 14510bc8b82ee4c5280e53b83f4b2619 | 0 | 0 | 3 | 0 | 0 | 0 | f__NS11-12_marine_group |
| f6bd789101c9a3aa7b4b4ea96510653b | 0 | 0 | 3 | 0 | 0 | 0 | f__Flavobacteriaceae |
| 4380def8d7b31f96dd6d4f97ee10a2aa | 0 | 0 | 3 | 0 | 0 | 0 | f__Flavobacteriaceae |
| a3ef7b542734d0bbade2073bc4c4badf | 0 | 0 | 3 | 0 | 0 | 0 | f__Clostridiaceae |
| b72e6309f0689f7d2d3d72858ba3b01d | 0 | 0 | 3 | 0 | 0 | 0 |  |
| bf2e0e9ab63e126b1eb71bfab1777bb6 | 0 | 0 | 3 | 0 | 0 | 0 | f__Rhizobiaceae |
| 1da9f0abbd425d52be212b975d7887e6 | 3 | 0 | 0 | 0 | 0 | 0 | f__Solirubrobacteraceae |
| 9f9d51945dacd79960b3eb2568b9b80d | 3 | 0 | 0 | 0 | 0 | 0 | f__Blfdi19 |
| 414254f3f1999b60659bb1db759fe34d | 3 | 0 | 0 | 0 | 0 | 0 | f__Microscillaceae |
| fe951b6dbc8c34622d2b89320ba15288 | 0 | 0 | 0 | 0 | 3 | 0 | f__Beijerinckiaceae |
| 6b835e9b50885289dafb6e9b1477fb37 | 0 | 0 | 0 | 0 | 3 | 0 | f__Limnochordaceae |
| 12f515dac195f0264d6a386d5ae2e6ea | 0 | 0 | 0 | 0 | 3 | 0 | f__Saccharimonadales |
| 46c8b1a17a10015095f9cae4c959bf4d | 0 | 0 | 0 | 3 | 0 | 0 | f__Hyphomicrobiaceae |
| b8fef76d73489eecb09395abe5aa55fe | 0 | 0 | 0 | 3 | 0 | 0 | f__Cyclobacteriaceae |
| 719bf33bbce046eea2df90846f314d29 | 0 | 0 | 0 | 3 | 0 | 0 | f__Rhizobiaceae |
| d34192dfb18c15d2b41ef6c580a85671 | 0 | 3 | 0 | 0 | 0 | 0 | f__Bacillaceae |
| eb0dbb229496489c81d8b5b90d5c6d99 | 0 | 3 | 0 | 0 | 0 | 0 | f__Bacillaceae |
| 94ea4c9cfcc094dd45f9d0248badce87 | 0 | 3 | 0 | 0 | 0 | 0 | f__Fibrobacteraceae |
| 46c2cba4e1a6677c2be655f309da4852 | 0 | 3 | 0 | 0 | 0 | 0 | f__Bacillaceae |
| f8348381a5553235e7b219d9b1b3f517 | 0 | 0 | 0 | 0 | 0 | 2 | f__Sphingomonadaceae |
| e2c8d37f2bfb99d924334f64c2872d23 | 0 | 0 | 0 | 0 | 0 | 2 | f__Kapabacteriales |
| 777c24947fab6565dabf4b18cdb98cc8 | 0 | 0 | 0 | 0 | 0 | 2 | f__Diplorickettsiaceae |
| 9ec1c214fc430953b5e8945ffa39bf79 | 0 | 0 | 2 | 0 | 0 | 0 | f__Flavobacteriaceae |
| b0a2b7286898e8ef9e2b895628fd3cbd | 0 | 0 | 2 | 0 | 0 | 0 | f__Ilumatobacteraceae |
| 49b5e64a02cc2763595c13c52f8a9d45 | 0 | 0 | 2 | 0 | 0 | 0 | f__Cyclobacteriaceae |
| 68e9443f944fee0086ba9a50b38949c4 | 0 | 0 | 2 | 0 | 0 | 0 | f__Sandaracinaceae |
| 2d59e96c717565de83bd90b1ca6839a9 | 0 | 0 | 2 | 0 | 0 | 0 | f__Devosiaceae |
| 30af83150a37978c11d60b44f423b056 | 0 | 0 | 2 | 0 | 0 | 0 | f__Promicromonosporaceae |
| 5d2290f81534f73c1ed8bd7a19a51c66 | 0 | 0 | 2 | 0 | 0 | 0 | f__Flavobacteriaceae |
| bb8663ef84833d0d80746074ccbdc931 | 0 | 0 | 2 | 0 | 0 | 0 | f__Peptostreptococcales-Tissierellales |
| 64bf264dbe83f97c600c9266327de810 | 0 | 0 | 2 | 0 | 0 | 0 | f__Gaiellaceae |
| c3416fe092f0e5f22997c7fef9d06e24 | 0 | 0 | 2 | 0 | 0 | 0 | f__bacteriap25 |
| 9160962eca4aa7a5f65d3f2a1ed86949 | 2 | 0 | 0 | 0 | 0 | 0 | f__Oxalobacteraceae |
| 63e5b2ab6d2d5dd8e92a1af1b4a7f706 | 2 | 0 | 0 | 0 | 0 | 0 | f__Blfdi19 |
| e29da22e468957cc393f1b07b7c9aab9 | 2 | 0 | 0 | 0 | 0 | 0 | f__Xanthomonadaceae |
| 21ca0b30f130fa1c5443d4b434a8957f | 2 | 0 | 0 | 0 | 0 | 0 | f__Verrucomicrobiaceae |
| 6bc1e7ddf54bc60e4395925871a331bf | 2 | 0 | 0 | 0 | 0 | 0 | f__SJA-28 |
| 258806159dd3521efffb23072cea52d8 | 2 | 0 | 0 | 0 | 0 | 0 | f__Xanthomonadaceae |
| 2a9fb08e2531ad45c1f4f66dc8a23263 | 2 | 0 | 0 | 0 | 0 | 0 | f__Opitutaceae |
| 59f945de75375025da74c3c5e8819791 | 2 | 0 | 0 | 0 | 0 | 0 | f__Steroidobacteraceae |
| 80035208b13cdcaa445e4abf32bed7b7 | 2 | 0 | 0 | 0 | 0 | 0 | f__Thermoanaerobaculaceae |
| 12aba1ff53af96277d29c4e031603062 | 2 | 0 | 0 | 0 | 0 | 0 | f__Neisseriaceae |
| 8800f227bab6aa89fa7dacb8223947a0 | 0 | 0 | 0 | 0 | 2 | 0 | f__Xanthomonadaceae |
| da63114c508350893c0f47a97d6d0e3d | 0 | 0 | 0 | 2 | 0 | 0 | f__Microscillaceae |
| f9f843cb5f01463aacdf1c7b4e980e05 | 0 | 0 | 0 | 2 | 0 | 0 | f__env.OPS_17 |
| 4ade451ffe8ebd722b5c9b77ff6535bf | 0 | 0 | 0 | 2 | 0 | 0 | f__Oxalobacteraceae |
| e0c711f56cf9422fb0e387c3d1904c3a | 0 | 0 | 0 | 2 | 0 | 0 | f__Xanthomonadaceae |
| ad886de3f8ee8c53e5d085d8374c163c | 0 | 2 | 0 | 0 | 0 | 0 | f__Caulobacteraceae |
| f40e228976975a135947d3c88b377199 | 0 | 2 | 0 | 0 | 0 | 0 | f__Bdellovibrionaceae |
| fe6d81d1e4af5a71071b8ef0d2b6cc51 | 0 | 2 | 0 | 0 | 0 | 0 | f__Xanthomonadaceae |
| 9cf5f2b72a044b35e78fde181b131eb1 | 0 | 2 | 0 | 0 | 0 | 0 | f__Pseudomonadaceae |
| 905ab1f69f45fded8fde72c1365f63ba | 0 | 2 | 0 | 0 | 0 | 0 | f__Cyclobacteriaceae |
| 03108aaaeca88690873c16a27d3e3c8c | 0 | 2 | 0 | 0 | 0 | 0 | f__Babeliaceae |
| 71b5d35741d3a25bb817bdb01fb7a1f6 | 0 | 0 | 0 | 1 | 0 | 0 | f__Caulobacteraceae |
| 941665f340a19543a69a2a3340036bec | 0 | 1 | 0 | 0 | 0 | 0 | f__Bacillaceae |

| Table S 11: Bacterial species found in various samples | | | | | | | |
| --- | --- | --- | --- | --- | --- | --- | --- |
| #OTU ID | SG-B-NS | SG-B-S | PM-B-NS | PM-B-S | Saline soil | Non-saline soil | species |
| d7185fab0700d464cf2457d47b5dc082 | 0 | 0 | 124 | 0 | 0 | 234 |  |
| 7fe4da75dc9ae89419b82ef36d1a0fff | 0 | 0 | 0 | 214 | 0 | 0 |  |
| cc4b0f8272e5e7be2afde61ec81c1f4a | 0 | 0 | 91 | 0 | 116 | 0 |  |
| e1cd7e492e853fb66f916798f9838d34 | 196 | 0 | 0 | 0 | 0 | 0 |  |
| 7923980b51ffb4ccb6f18d1e53a0be86 | 19 | 25 | 31 | 28 | 62 | 26 |  |
| c5428c6f0aae1473b56d2e4cf67f84ab | 0 | 0 | 0 | 0 | 0 | 172 |  |
| bc1430bdc9a2b6cb953436e71f969745 | 0 | 33 | 59 | 73 | 0 | 0 |  |
| c55225af0e75ad87bc10c0fc3e719948 | 9 | 29 | 33 | 34 | 31 | 25 |  |
| de234ba064107c25f290664f1d2a912b | 0 | 151 | 0 | 0 | 0 | 0 |  |
| f3be38725a30178af3c615367d39b1da | 0 | 146 | 0 | 0 | 0 | 0 |  |
| fc99988b65c9772f674e63a73d5c2b40 | 0 | 0 | 142 | 0 | 0 | 0 |  |
| b3cf4a5c3531d61ec80a52fa950714d9 | 0 | 0 | 0 | 142 | 0 | 0 |  |
| c170a275313d3b07b992368d4f362923 | 0 | 0 | 135 | 0 | 0 | 0 |  |
| eb24f9a8cb47003458aaf1f3dd293264 | 0 | 0 | 0 | 0 | 0 | 131 |  |
| 10df45687fc10d18327f470e38e431b6 | 0 | 0 | 0 | 0 | 0 | 131 |  |
| 244d781e5d3b4ae6cb73675661d064de | 0 | 48 | 23 | 0 | 16 | 44 |  |
| 06f91809646e68eec9ed50029b03255b | 0 | 0 | 0 | 71 | 60 | 0 |  |
| 30be997dfe7489085e7833c10bb7eb1c | 0 | 125 | 0 | 0 | 0 | 0 |  |
| ea0a61307d6dc17c662000b0199e23ba | 0 | 125 | 0 | 0 | 0 | 0 |  |
| c97397ac6462bb8ec967290f811dfddd | 0 | 0 | 0 | 85 | 0 | 39 | s__Pseudorhodoplanes_sinuspersici |
| 8aa0db72991b55d6b082999f4a9dab6d | 0 | 0 | 122 | 0 | 0 | 0 |  |
| 8f8075c02cab44a7cd0b8e7d852abd25 | 0 | 0 | 0 | 0 | 0 | 120 |  |
| a6baa6926204fad76b8acb0d12de5afe | 0 | 0 | 0 | 114 | 0 | 0 | s__uncultured_bacterium |
| f907e399af9e0dbf1a6bb91263e382cf | 0 | 0 | 0 | 0 | 113 | 0 | s__uncultured_Chloroflexi |
| 556b3495ddee561a8676044529b8f6f8 | 0 | 0 | 0 | 113 | 0 | 0 | s__Arthrobacter_crystallopoietes |
| 0e6fe837f17fd744fedf314ee992ea39 | 0 | 0 | 109 | 0 | 0 | 0 |  |
| bbd5fd0bd3c1c5a80ba40caeb392bad3 | 0 | 0 | 108 | 0 | 0 | 0 |  |
| bff45b8c5c070a2cb7698645ea1d52fd | 0 | 0 | 0 | 0 | 106 | 0 |  |
| 8aa161f26698c863f7d0b457e8d23abd | 0 | 0 | 66 | 0 | 39 | 0 | s__uncultured_Erythrobacteraceae |
| 0091a0d823647c3159434d7c42ecce36 | 40 | 0 | 64 | 0 | 0 | 0 | s__Pseudomonas_sp. |
| 916e822008220f5af0f5bb24fab388d0 | 0 | 104 | 0 | 0 | 0 | 0 |  |
| 2855119d0b771458ba21dbb711039ead | 39 | 0 | 0 | 0 | 0 | 63 | s__uncultured_Alphaproteobacteria |
| 8d157bda3235ad6a63998384e053fc65 | 0 | 0 | 102 | 0 | 0 | 0 |  |
| b31bcd8ffe56b005b89aaa69e376246a | 0 | 0 | 102 | 0 | 0 | 0 |  |
| 5a5d78bfd48bf801bf611deb7620eb20 | 0 | 0 | 0 | 0 | 0 | 101 |  |
| d05e89d615a1acf9f70ce2d6b1b7fb1f | 0 | 0 | 0 | 0 | 0 | 100 |  |
| 396a9909455aceef07c8c680d602fdaf | 0 | 0 | 100 | 0 | 0 | 0 |  |
| aa92f210f241812cd569c30968caceb9 | 0 | 0 | 0 | 0 | 0 | 97 |  |
| 37e8d1991f498e6688512607af8f4823 | 0 | 0 | 0 | 0 | 97 | 0 | s__Olivibacter_soli |
| 658919892336d3e1d6dfa44c147ed95b | 96 | 0 | 0 | 0 | 0 | 0 |  |
| 9b598230c51aab782c6b2f446ad94061 | 0 | 0 | 0 | 96 | 0 | 0 |  |
| 3b2413b4c009bb6fba27b8e014196baf | 0 | 0 | 0 | 0 | 0 | 93 |  |
| 9c4932717b4611696362a46ea5831906 | 0 | 0 | 0 | 93 | 0 | 0 |  |
| a7a716d3f1f5068d75ff52092da5b868 | 0 | 0 | 0 | 92 | 0 | 0 |  |
| 2cb9b8cc10da9544ff8ac2e97be0388f | 0 | 0 | 57 | 0 | 0 | 34 |  |
| f7fea7e337301dbc31032687018b70e0 | 91 | 0 | 0 | 0 | 0 | 0 |  |
| ff8f1635fad3fc4962045c700d3ca8ec | 0 | 91 | 0 | 0 | 0 | 0 |  |
| 599acc6f1afdd44cc78904c0fe8980fb | 0 | 0 | 0 | 0 | 0 | 89 |  |
| cc2f9378d537491cd5630efc9fd73f2c | 89 | 0 | 0 | 0 | 0 | 0 | s__metagenome |
| 11a00aa173e1a059179353abbcdd385e | 0 | 89 | 0 | 0 | 0 | 0 |  |
| eb817b665babf101b39a77112cd45eac | 0 | 0 | 0 | 88 | 0 | 0 |  |
| 598a91b2745e606e2592a69cb543f1c1 | 0 | 0 | 87 | 0 | 0 | 0 |  |
| f28bbc4f2f8cb3acefc461c8fc4f6897 | 0 | 0 | 34 | 0 | 53 | 0 |  |
| a9def51ab2c51af3ab6012116acd76ea | 45 | 0 | 41 | 0 | 0 | 0 | s__Algoriphagus_terrigena |
| de5b441374ec6d5677e1f5ae3c5a6262 | 0 | 86 | 0 | 0 | 0 | 0 | s__uncultured_Ohtaekwangia |
| 61c062f4b290724e46f5b65d74767639 | 0 | 0 | 0 | 0 | 0 | 84 | s__uncultured_Planctomycetaceae |
| 294378e89161a13b5f0fb35a1f5179af | 84 | 0 | 0 | 0 | 0 | 0 |  |
| b2812018067a3f2f9905a48848b8b3bc | 84 | 0 | 0 | 0 | 0 | 0 |  |
| b4dd5a553d1ee799cd4cd784e4eee36c | 0 | 0 | 0 | 84 | 0 | 0 |  |
| 3afd6400a21c5af9e56f7b383af9375f | 83 | 0 | 0 | 0 | 0 | 0 |  |
| 648c3ac221be7bc96b685d5dd5c51c0a | 0 | 0 | 0 | 83 | 0 | 0 |  |
| 777cbdcfaecb19ab4a39d7a442743153 | 0 | 83 | 0 | 0 | 0 | 0 |  |
| b76cb035aa71a3ea186e1108e04155a6 | 0 | 0 | 54 | 28 | 0 | 0 |  |
| 3dd174f634f3ab7817fd9be3ad8d209e | 0 | 82 | 0 | 0 | 0 | 0 |  |
| b11c5875d09a0b64518a469d346716a3 | 81 | 0 | 0 | 0 | 0 | 0 |  |
| b8fc8a43954916ba7a1e3e2649af45f9 | 81 | 0 | 0 | 0 | 0 | 0 |  |
| 043210b0814a3e729fca885444189b3f | 0 | 0 | 0 | 81 | 0 | 0 |  |
| 5500942e158a52f90622d6d4ce9bcfea | 0 | 0 | 0 | 81 | 0 | 0 | s__Nannocystis_exedens |
| 815ad6a35693289b5090a5a4f67f8710 | 0 | 0 | 80 | 0 | 0 | 0 |  |
| b87467abc21050a7cc8f7fcdfc8e67b1 | 80 | 0 | 0 | 0 | 0 | 0 |  |
| 55e2a73b4d6c091290157e49e6f5caa3 | 0 | 80 | 0 | 0 | 0 | 0 |  |
| 9d788648abe8c195c8de0c390ad7e7a0 | 0 | 0 | 79 | 0 | 0 | 0 |  |
| 557fcab2f879b90df5d27d489e5648ee | 0 | 0 | 79 | 0 | 0 | 0 |  |
| d0b6598c22877613069d58dbab2ee899 | 0 | 79 | 0 | 0 | 0 | 0 |  |
| c886aac54206b432a0974d3701274ba7 | 0 | 79 | 0 | 0 | 0 | 0 |  |
| 9206a600e32dfa88c94eea9b2f4969c1 | 14 | 7 | 12 | 16 | 20 | 9 |  |
| a3f470b531f45487fe79b5daa3c7f1f9 | 0 | 78 | 0 | 0 | 0 | 0 | s__uncultured_Erythrobacteraceae |
| 462cadae2665f8d5b0c3a70455629eeb | 0 | 78 | 0 | 0 | 0 | 0 |  |
| caec11b476ff87554acff397ea9e9a08 | 77 | 0 | 0 | 0 | 0 | 0 |  |
| 9ae85ad1250e7ded525a397258a28b5e | 77 | 0 | 0 | 0 | 0 | 0 |  |
| 7924307411823b85c3907b1be1b28c62 | 0 | 77 | 0 | 0 | 0 | 0 |  |
| cf6e6aa8c2d7c329b2496aa077998c8e | 76 | 0 | 0 | 0 | 0 | 0 |  |
| 1db2422455e8f3df912ab3437a00f06a | 75 | 0 | 0 | 0 | 0 | 0 |  |
| 2acd6450737e25c9699c8a72e442a172 | 75 | 0 | 0 | 0 | 0 | 0 |  |
| 30f43081afcb8086a42aca8e27398e3f | 0 | 75 | 0 | 0 | 0 | 0 |  |
| b3dd863b19db3801af3fa2149563606d | 0 | 0 | 74 | 0 | 0 | 0 |  |
| 90d7f11719d67def2d2e5cebad369428 | 0 | 0 | 74 | 0 | 0 | 0 |  |
| fe63b9e034690a4076b090684effbab3 | 73 | 0 | 0 | 0 | 0 | 0 |  |
| 1fefcfaf79cbc4101da0d9f1aa8b1332 | 0 | 0 | 0 | 73 | 0 | 0 |  |
| cc425fb21f457644b34c6c1678c4ac34 | 72 | 0 | 0 | 0 | 0 | 0 | s__metagenome |
| 1d6238af1c4b79ad0a4d301ec45edcf1 | 0 | 0 | 71 | 0 | 0 | 0 |  |
| add8bef816320532820580b08ef57a47 | 0 | 0 | 71 | 0 | 0 | 0 |  |
| c283c69f6716a7ece7e92615d30d2ced | 0 | 0 | 0 | 0 | 71 | 0 |  |
| 0d8ce88f3f9fad127f790f08e52b3514 | 0 | 0 | 0 | 71 | 0 | 0 |  |
| 27a48a5fe4071678e76b832fa505fc42 | 70 | 0 | 0 | 0 | 0 | 0 |  |
| cdb0a1d1d4bc081806b44029bcecd72b | 0 | 0 | 0 | 0 | 70 | 0 |  |
| c5c3cb2784127776f95dbb2f117ca221 | 0 | 0 | 0 | 70 | 0 | 0 | s__uncultured_Planctomycetaceae |
| cd1eda4643c23b038819442c68c58a4c | 0 | 0 | 0 | 0 | 0 | 69 |  |
| 965c32de4c91bac3aa5b343df8e79d2c | 0 | 0 | 0 | 0 | 69 | 0 |  |
| 6535e32087e6a29af670d5599131e6d2 | 0 | 0 | 0 | 0 | 69 | 0 | s__Agromyces_fucosus |
| f681268aecb67982c8dbea9141dfd0b8 | 0 | 0 | 68 | 0 | 0 | 0 | s__uncultured_Planctomycetia |
| 4bb0726e20f79116635a615f2dd9cc1f | 67 | 0 | 0 | 0 | 0 | 0 |  |
| 360eaffcc796450933e87c41417f5372 | 67 | 0 | 0 | 0 | 0 | 0 | s__Nocardioides_dilutus |
| ab795859bfb221917c4869bcda97a327 | 67 | 0 | 0 | 0 | 0 | 0 | s__uncultured_bacterium |
| 9c99727d43bff46769b70b91054f5b25 | 0 | 0 | 0 | 67 | 0 | 0 |  |
| 6501bceab3629a591b9deb7b1f07761c | 0 | 0 | 0 | 0 | 0 | 66 |  |
| bc73c66027bacd5cd2d789ef11d440ce | 0 | 0 | 0 | 0 | 0 | 66 |  |
| 2299fb9ffd84737e9ff4ed08a30bb20a | 0 | 0 | 0 | 0 | 0 | 66 |  |
| afa225e647e496ec745521370ed62431 | 0 | 0 | 0 | 0 | 0 | 66 |  |
| 3b5ebf65eecebf5c7736046f87ad8cd7 | 66 | 0 | 0 | 0 | 0 | 0 |  |
| 3e3e4eec9e50a4872136f86f2c241a2b | 0 | 66 | 0 | 0 | 0 | 0 |  |
| 2783941706df39d4138058184c23af4c | 0 | 0 | 65 | 0 | 0 | 0 |  |
| b87172d81a55fdd290a67f821cd1082a | 65 | 0 | 0 | 0 | 0 | 0 |  |
| ea010fc184c0e4afee84186bfdfc7b63 | 0 | 65 | 0 | 0 | 0 | 0 | s__uncultured_bacterium |
| 5468d90654c8538e615198ffa331bc53 | 0 | 0 | 0 | 0 | 64 | 0 |  |
| 77f278a9b694b4e24d423d3412f2c69c | 0 | 0 | 0 | 0 | 64 | 0 |  |
| 85509d4725df11ea80195e285c31214d | 0 | 64 | 0 | 0 | 0 | 0 |  |
| 6b25eed6df765ce879d98a9a3da2d8ed | 0 | 0 | 63 | 0 | 0 | 0 | s__uncultured_Ohtaekwangia |
| 91e3267f5ccaea0e662547b07ffe92df | 63 | 0 | 0 | 0 | 0 | 0 |  |
| 14f321fed6cc24069aa14c327c90884e | 63 | 0 | 0 | 0 | 0 | 0 |  |
| 0ca447c539007a0b8610419c7178f751 | 0 | 0 | 0 | 0 | 63 | 0 |  |
| b335e6c4eaccc9e34161a6f265e4fa67 | 0 | 0 | 0 | 0 | 63 | 0 |  |
| da1eb90b40a6e4198c0ce7ca54c9d4d1 | 0 | 63 | 0 | 0 | 0 | 0 | s__Allorhizobium_oryzae |
| 5a4b81c23c0c94391937a2d09c11c292 | 0 | 0 | 0 | 0 | 0 | 62 |  |
| 5e813041b43184acd847d8c43daa04e5 | 0 | 0 | 62 | 0 | 0 | 0 |  |
| 03baef37d44e996f98f12ed5050bff87 | 0 | 0 | 0 | 0 | 62 | 0 |  |
| 24dc458655e7885e09183d4401352642 | 0 | 0 | 0 | 62 | 0 | 0 |  |
| 83837c5b6fd6381c9db676256289db72 | 0 | 0 | 0 | 62 | 0 | 0 | s__uncultured_Ohtaekwangia |
| 34eaae98386024cd3ae032af7b1eecc4 | 0 | 62 | 0 | 0 | 0 | 0 | s__uncultured_Chloroflexi |
| 1a9b10d5ed0696d198b1317715ada85b | 0 | 62 | 0 | 0 | 0 | 0 |  |
| e45d7a2e0c415f5f80840d9aeccfddae | 0 | 0 | 0 | 0 | 0 | 61 |  |
| a488390ab16ac5529e166a698e380a33 | 0 | 0 | 61 | 0 | 0 | 0 |  |
| ed4069b2e063a83a43a4b37859c8d15c | 0 | 61 | 0 | 0 | 0 | 0 |  |
| 2be9be387e8549f2a12d85fd8ee169c8 | 0 | 61 | 0 | 0 | 0 | 0 |  |
| c11918a1924a31fff9b6049733dfeb3a | 0 | 61 | 0 | 0 | 0 | 0 |  |
| 4cee34a09f59b236a61d34d5e9f39cdf | 60 | 0 | 0 | 0 | 0 | 0 |  |
| d4e027c4ef517cd601bdde815f88bdc5 | 0 | 0 | 0 | 60 | 0 | 0 | s__uncultured_Hyphomicrobium |
| 9920d00c31da464fa3fe94be8a01a208 | 0 | 0 | 0 | 0 | 0 | 59 |  |
| bcd9700d4fc15c838bfe01d0c6b6f0d2 | 0 | 0 | 0 | 0 | 0 | 59 |  |
| e42d24cfb93825366121de08f3c9fe98 | 0 | 31 | 0 | 0 | 0 | 28 |  |
| 24d566ca93f1c19f72de1f40c4bb0f22 | 0 | 0 | 59 | 0 | 0 | 0 | s__uncultured_Planctomycetaceae |
| 25003d46de6e6a69d3b9867990b94a0f | 0 | 0 | 0 | 0 | 59 | 0 |  |
| 907b33aca73d876cbd2b2c8c98973462 | 0 | 0 | 0 | 59 | 0 | 0 |  |
| c01f26d7a670bebc1b3836841ab30dda | 0 | 59 | 0 | 0 | 0 | 0 | s__uncultured_bacterium |
| 8b71d2a9f93ad9cafc16609a5c19d569 | 0 | 0 | 0 | 0 | 58 | 0 |  |
| cd30c4660f15897daff64ba23c3edf02 | 0 | 0 | 0 | 0 | 58 | 0 |  |
| f3c665d9b597e0775cabaa9c6738c31d | 0 | 58 | 0 | 0 | 0 | 0 |  |
| 6ae126bc191cf9205c1cad45defed455 | 0 | 0 | 0 | 0 | 57 | 0 |  |
| 773266dded97b367a06b5bb7c252cbab | 0 | 0 | 0 | 57 | 0 | 0 | s__uncultured_Chloroflexi |
| 361ad502a751294aa41caa728170bc67 | 0 | 57 | 0 | 0 | 0 | 0 |  |
| 9126defecac389c138629d30c982eb48 | 0 | 57 | 0 | 0 | 0 | 0 | s__uncultured_Acidimicrobiia |
| f3386a8006432d95687523c66a66f751 | 0 | 0 | 0 | 0 | 0 | 56 |  |
| 3c25a6498a98a8800fd7143739770d6a | 0 | 0 | 0 | 0 | 0 | 56 | s__Agromyces_fucosus |
| b98dcf537a4c0c2b395fbfad85512c4d | 0 | 0 | 56 | 0 | 0 | 0 |  |
| 32e75966e275b68c3c07bfa4d095a9da | 56 | 0 | 0 | 0 | 0 | 0 | s__uncultured_gamma |
| 2c3af845cfa49dd79ed12007da5c5122 | 56 | 0 | 0 | 0 | 0 | 0 |  |
| 8cfe07b3d55b8ce346b9cc42e3a5ec47 | 0 | 56 | 0 | 0 | 0 | 0 |  |
| 326ad8a86baac6ad65d81bbed87ae506 | 0 | 0 | 0 | 0 | 0 | 55 | s__uncultured_Firmicutes |
| edd7b582bbc98963401fb7e38d3391a8 | 0 | 0 | 44 | 0 | 11 | 0 |  |
| 69bc3b35ea6dbf843a1e5b9a0beb0bdc | 55 | 0 | 0 | 0 | 0 | 0 |  |
| 594a5ffc5125217753696c0ccb2b8e8b | 55 | 0 | 0 | 0 | 0 | 0 |  |
| 9a116d9b108bf6b6f3626bdb6d5b0137 | 55 | 0 | 0 | 0 | 0 | 0 |  |
| 63dc5d9214c58331a26be0f4dce79c10 | 0 | 55 | 0 | 0 | 0 | 0 | s__Algoriphagus_resistens |
| 460aa5d3918f22d14dfcf5c4be46477f | 0 | 0 | 0 | 0 | 54 | 0 |  |
| 4e3802a01bc841da4da6e1b758f0043b | 0 | 0 | 0 | 0 | 54 | 0 | s__Glycomyces_algeriensis |
| c7128600d15a28d6616da72f3f3dcc5e | 0 | 0 | 0 | 54 | 0 | 0 | s__uncultured_Planctomycetaceae |
| 2bd4c659591dbc6837cc569fac6c6c47 | 0 | 0 | 0 | 0 | 0 | 53 |  |
| 4f62887ed0d53e45540384175e83450b | 0 | 0 | 0 | 0 | 19 | 34 |  |
| c22025f09eb5f1e78a75aa63be5b160c | 0 | 0 | 53 | 0 | 0 | 0 |  |
| 125948970c265c1fd0ef0d4dc1192c57 | 0 | 0 | 53 | 0 | 0 | 0 |  |
| 702dedf591f0fa1c272e4b123059c28b | 0 | 0 | 53 | 0 | 0 | 0 |  |
| 8b9e3ee5e8f133db10a412971adca3f4 | 0 | 0 | 0 | 0 | 53 | 0 | s__Nocardioides_exalbidus |
| 97c01b3ece53398798092b242d98c090 | 0 | 0 | 0 | 0 | 53 | 0 |  |
| 01990585b45e17df990dbb5384971406 | 0 | 0 | 0 | 0 | 0 | 52 |  |
| efdbde2e098c28865367d44a998f0cb5 | 52 | 0 | 0 | 0 | 0 | 0 | s__Pseudorhodoplanes_sinuspersici |
| 3f6d0b3485db5cd259a1c31c5c9361c5 | 0 | 52 | 0 | 0 | 0 | 0 |  |
| f7fc3c784eed80a44aa41949519494d9 | 0 | 0 | 0 | 0 | 0 | 51 | s__uncultured_Ohtaekwangia |
| 2e5b5e2d81f7b276d03a272e0d8854ff | 0 | 0 | 0 | 0 | 0 | 51 |  |
| c16be9a8a574e8f694107bf004ad731a | 0 | 0 | 51 | 0 | 0 | 0 |  |
| 1a53fdaf19962798d52fde0fe87be470 | 51 | 0 | 0 | 0 | 0 | 0 |  |
| 299425985e889226ba78645a9632625f | 0 | 0 | 0 | 0 | 51 | 0 | s__Pseudorhodoplanes_sinuspersici |
| 7538dbc09a7360938f8df893f8e07d73 | 0 | 0 | 0 | 0 | 51 | 0 |  |
| 0b79f01dbc564e3f997ed2ed56e10491 | 0 | 51 | 0 | 0 | 0 | 0 |  |
| c00a4b98032719e55cf7283290a14fd8 | 0 | 0 | 0 | 0 | 0 | 50 | s__metagenome |
| 2566e2487d16fecc959f56567675bda7 | 0 | 0 | 50 | 0 | 0 | 0 | s__Flavobacteriaceae_bacterium |
| 378cb79c32d2a5a05d8e9a7a2b2883b0 | 0 | 0 | 50 | 0 | 0 | 0 |  |
| 55652458c7c4ffb2db4e9581208be010 | 0 | 0 | 50 | 0 | 0 | 0 |  |
| b73f5dccf144623386281bfea004681e | 0 | 0 | 50 | 0 | 0 | 0 |  |
| 4ee1f519ea881295e783d7f23dfdb1de | 0 | 50 | 0 | 0 | 0 | 0 | s__Agromyces_indicus |
| ed42a114c72d69e31d2c08535168c778 | 0 | 50 | 0 | 0 | 0 | 0 | s__uncultured_Firmicutes |
| dc9481c0920065f838672c05e3d0cd07 | 0 | 0 | 49 | 0 | 0 | 0 |  |
| b9e881bc1eb53c0eff39cca3f1597f96 | 49 | 0 | 0 | 0 | 0 | 0 |  |
| 2b20f121341fc68ec11bd506c94a0281 | 0 | 0 | 0 | 0 | 49 | 0 |  |
| 763ec14d4ab973ab827c3d05aac9f98b | 0 | 0 | 0 | 49 | 0 | 0 |  |
| 836c33daaf6535ee2641061fd65b81b9 | 0 | 0 | 0 | 49 | 0 | 0 | s__uncultured_soil |
| 1c4103cc7a0df340b09658e9768b5b7c | 0 | 0 | 48 | 0 | 0 | 0 | s__Flavobacterium_qiangtangense |
| 33d57f7cac6c2d38daf26eabed6960a1 | 48 | 0 | 0 | 0 | 0 | 0 | s__Luteolibacter_arcticus |
| ad2a1ad40c0eaadf58cae3b63dc32826 | 0 | 0 | 0 | 0 | 48 | 0 |  |
| 6b9ce472cf8a92f6691242f02e9961e3 | 0 | 0 | 0 | 48 | 0 | 0 |  |
| ddb772a897f302124c79e250b1ba90b2 | 0 | 48 | 0 | 0 | 0 | 0 |  |
| 8b5c869a68e2f51383c246aac193837d | 0 | 0 | 0 | 0 | 0 | 47 |  |
| 301493fa2212357b7789de81afc28981 | 0 | 0 | 47 | 0 | 0 | 0 |  |
| daffdded28f29cfcbc1b561c17ac8185 | 0 | 0 | 0 | 0 | 47 | 0 | s__Desertimonas_flava |
| bcb0052db81230562c235ea4c07c1b00 | 0 | 0 | 0 | 0 | 47 | 0 | s__uncultured_Cytophagia |
| 24e26d94379a9dff41373048ad52aec9 | 0 | 0 | 0 | 26 | 21 | 0 |  |
| 082b96163cdecb3c2519aa1e2ff554f2 | 0 | 0 | 0 | 47 | 0 | 0 | s__uncultured_Ohtaekwangia |
| 05e386f173205e17481822d4c77a7990 | 0 | 0 | 0 | 0 | 0 | 46 | s__delta_proteobacterium |
| e9252ed1fbe56eb1884dd4b2b501c054 | 46 | 0 | 0 | 0 | 0 | 0 |  |
| 3e8a03caf9c6c228dcebd6b1a352d303 | 0 | 0 | 0 | 46 | 0 | 0 | s__Algoriphagus_resistens |
| 091a0dbf947e3d56d93f9f19ab976fab | 0 | 0 | 45 | 0 | 0 | 0 | s__Bacillus_funiculus |
| 23dc8815d75c13b48028cdbdb3669fee | 45 | 0 | 0 | 0 | 0 | 0 | s__uncultured_bacterium |
| e5b6913f0a0baddb3ff6d0a2e7e295d4 | 0 | 0 | 0 | 0 | 45 | 0 |  |
| d649d80c6f15016f8644b7cfe08d238e | 0 | 0 | 0 | 45 | 0 | 0 | s__Solirubrobacterales_bacterium |
| 82de8cb25c53f963c2b6a6f3ef81e229 | 0 | 45 | 0 | 0 | 0 | 0 |  |
| 036581ca41f9ab0a2979d638f3fbfdb7 | 0 | 0 | 0 | 0 | 0 | 44 | s__uncultured_Planctomycetia |
| 03facaf499a0a68fdf985a1e29b2ebcf | 0 | 0 | 44 | 0 | 0 | 0 |  |
| b7d77124eadd3aac038bbb189f927707 | 0 | 0 | 44 | 0 | 0 | 0 |  |
| 5e9fea0d0f12736643e0a0b58a431399 | 0 | 0 | 44 | 0 | 0 | 0 |  |
| 1d3a083f3fce2192213a2d9f647a5424 | 44 | 0 | 0 | 0 | 0 | 0 | s__metagenome |
| b1bc01a5f9bdf17392aeb5cd03b5f3a4 | 0 | 0 | 0 | 0 | 44 | 0 |  |
| 4c09b1fa079132e973521a5599ec79b2 | 0 | 0 | 0 | 0 | 44 | 0 |  |
| fc54a2c127cfe20a922746866d664c4a | 0 | 0 | 0 | 0 | 44 | 0 |  |
| 409a197a0ff95563ade9b4820fa03c66 | 0 | 0 | 0 | 44 | 0 | 0 |  |
| db0b2325bf82bd9f160db091fc437d7d | 0 | 44 | 0 | 0 | 0 | 0 | s__candidate_division |
| 0826bb50cc27946a1c1a1822db9916b2 | 0 | 0 | 0 | 0 | 0 | 43 |  |
| d8727caf21afb588622ad7e2e70b7a34 | 0 | 0 | 0 | 0 | 0 | 43 |  |
| ceded2d31498771c1f305c6ad74b7fa9 | 0 | 0 | 0 | 0 | 0 | 43 | s__uncultured_Lysobacter |
| 7e494e7eac0aa0a774894ace43432ce1 | 0 | 0 | 43 | 0 | 0 | 0 | s__metagenome |
| 97419902850ae622d16300fd0b77db03 | 0 | 0 | 43 | 0 | 0 | 0 |  |
| 36d887bcc30554a07f57e55e2d4753e1 | 15 | 0 | 28 | 0 | 0 | 0 |  |
| 37f65782c2bcdae5d10cb54b61fb1d35 | 43 | 0 | 0 | 0 | 0 | 0 | s__uncultured_bacterium |
| 1a08bf77f716d31b41bcbadc79db4d36 | 0 | 0 | 0 | 0 | 43 | 0 | s__Catelliglobosispora_koreensis |
| c2a65d3df280acb960865b1680b2c0e9 | 0 | 0 | 0 | 0 | 0 | 42 |  |
| ef0adae0c9d74c7a24960af3aacb3d3e | 0 | 0 | 42 | 0 | 0 | 0 |  |
| e0b5e14221c52958903c97ec9ae3fc2e | 0 | 0 | 42 | 0 | 0 | 0 | s__uncultured_proteobacterium |
| 47ee1e29e0f7db07e44f07b9207fba22 | 42 | 0 | 0 | 0 | 0 | 0 |  |
| fd97dfd310810e3b5e1f2237390d810e | 42 | 0 | 0 | 0 | 0 | 0 |  |
| c318e8b99ec0fa8fa9e260da01b976db | 42 | 0 | 0 | 0 | 0 | 0 | s__Luteibacter_sp. |
| 9b9e6f34f7357568fc0700330da651ce | 0 | 0 | 0 | 0 | 42 | 0 | s__uncultured_Planctomycetaceae |
| 68b80a5bbece6eb88a5252cc7ed4558c | 0 | 0 | 0 | 0 | 42 | 0 |  |
| d757a3995b879e6dd701c91f3ef7a310 | 0 | 0 | 0 | 42 | 0 | 0 |  |
| a943a33a9da9a8728e8ba49140349898 | 0 | 42 | 0 | 0 | 0 | 0 |  |
| ef8042cdcd8c441a0793b4c72d150962 | 0 | 0 | 0 | 0 | 0 | 41 |  |
| 475538e673aafb18c1327b87a25528cb | 0 | 0 | 41 | 0 | 0 | 0 | s__Agromyces_indicus |
| 957b7b13e31f150e0da15d4fddb3fa18 | 0 | 0 | 41 | 0 | 0 | 0 |  |
| 55d33be07f696f45fd9485edfa81b773 | 0 | 0 | 41 | 0 | 0 | 0 | s__Pedobacter_borealis |
| 616092cc1ad2a87b7e5eea7c16043776 | 0 | 0 | 0 | 0 | 41 | 0 | s__Nocardia_wallacei |
| ffa6a345879b133f0909cbb1f2542033 | 0 | 41 | 0 | 0 | 0 | 0 |  |
| 53f78cd25cfe3ff7fb7e2086d1263fe7 | 0 | 41 | 0 | 0 | 0 | 0 |  |
| 37fa774a4c91ef318e66373db12ca34a | 0 | 0 | 0 | 0 | 0 | 40 |  |
| 0e09ea7da3f2c8fef353e07ccfcc8fa0 | 0 | 0 | 40 | 0 | 0 | 0 |  |
| 94a2e34306b8e9539e9d7b6a500efda3 | 0 | 0 | 40 | 0 | 0 | 0 |  |
| 02b1b2a418ea490789b27ad2c2cd7087 | 40 | 0 | 0 | 0 | 0 | 0 |  |
| 29b03a752fb7536ad86bcd5db1bbc5d5 | 0 | 0 | 0 | 0 | 40 | 0 |  |
| c9fad30a91f421a919e97dfe2751ab08 | 0 | 0 | 0 | 0 | 40 | 0 |  |
| 565db7ee1a2d26ab6a0a1bb29a232d93 | 0 | 0 | 0 | 0 | 40 | 0 | s__uncultured_bacterium |
| a52c924757e376e0924c90c9c3bb8e14 | 0 | 0 | 0 | 0 | 40 | 0 |  |
| 8446c09ead8c6600737ed629355e1245 | 0 | 0 | 0 | 40 | 0 | 0 |  |
| d4cfeef3b8e9715b695f740b50a5dd8b | 0 | 40 | 0 | 0 | 0 | 0 | s__Pseudorhodoplanes_sinuspersici |
| 9b05bfc0551b241f29a56737ba4451d5 | 0 | 40 | 0 | 0 | 0 | 0 | s__Bacillus_sinesaloumensis |
| daceb9f23f795a99e1e846f4f9fae339 | 0 | 40 | 0 | 0 | 0 | 0 |  |
| aac96ec24a3eb607f2736c56af8d5644 | 0 | 40 | 0 | 0 | 0 | 0 |  |
| 7d19e16206ae5a213065cb75318857af | 39 | 0 | 0 | 0 | 0 | 0 | s__uncultured_soil |
| b8efc12945a51a28894544a8018eb2c9 | 0 | 0 | 0 | 0 | 39 | 0 |  |
| f21aa1757672afb0686842e7ae858f66 | 0 | 39 | 0 | 0 | 0 | 0 | s__Solirubrobacterales_bacterium |
| 0fbd93e29ac66c3844217a6fcbd2bf95 | 0 | 39 | 0 | 0 | 0 | 0 |  |
| 26a5087e8b88f695bf0d2d916dc85c03 | 0 | 0 | 0 | 0 | 0 | 38 |  |
| eb4227c2821375a378a60f35e6759809 | 38 | 0 | 0 | 0 | 0 | 0 | s__Pseudoxanthomonas_dokdonensis |
| 63126a6afdf3ba6668fe8a158e4971a8 | 0 | 0 | 0 | 38 | 0 | 0 | s__uncultured_Verrucomicrobiales |
| 4357e8df96a2b35c4903ebff5319b8d5 | 0 | 38 | 0 | 0 | 0 | 0 |  |
| 57f57e26e5b749073359795da5fce860 | 0 | 0 | 37 | 0 | 0 | 0 | s__uncultured_Thermomonas |
| 9b43f6bae5466a8aef4bc42444829eab | 37 | 0 | 0 | 0 | 0 | 0 |  |
| 4bd08219f1d60ab8a14442ffe6abdd4b | 37 | 0 | 0 | 0 | 0 | 0 | s__Agromyces_indicus |
| 8ab8636c490d02332022c18212df4a01 | 0 | 0 | 0 | 0 | 37 | 0 |  |
| d5b03f3e5e1575073f88ec105faa4881 | 0 | 0 | 0 | 0 | 37 | 0 | s__uncultured_Acidobacteriaceae |
| 4d29d0685ec98f5878835cf2ab784d9e | 0 | 0 | 0 | 0 | 37 | 0 | s__uncultured_Verrucomicrobiales |
| 3d94322cdbd5313445329a27bf78a923 | 0 | 37 | 0 | 0 | 0 | 0 | s__uncultured_Alphaproteobacteria |
| ed9c4b0e142044efaaace57b57158b4e | 0 | 37 | 0 | 0 | 0 | 0 | s__uncultured_Rhodospirillales |
| bb34c25c16288e8c33cd9dc61a21ba45 | 0 | 37 | 0 | 0 | 0 | 0 | s__uncultured_Pedobacter |
| 442fad2715993c386088afa9566a2fad | 0 | 0 | 0 | 0 | 0 | 36 | s__Bacillus_infernus |
| 88e9135512bbaa450d95ef46dc23e41d | 0 | 0 | 0 | 0 | 0 | 36 |  |
| 6f35e34f7121ac8842eb120a0fcc25e9 | 0 | 0 | 0 | 0 | 0 | 36 |  |
| 3456ba7dff876afff47c8c043a31eb5c | 36 | 0 | 0 | 0 | 0 | 0 |  |
| 2f12d0beebb0c686bd32b3e0e80dae0e | 36 | 0 | 0 | 0 | 0 | 0 | s__Nocardioides_exalbidus |
| 89a8bdd5818dc76514c0696b8e2de257 | 0 | 0 | 0 | 36 | 0 | 0 |  |
| 058ac5f94b8e9961d60f819cfdbafac0 | 0 | 36 | 0 | 0 | 0 | 0 |  |
| 7cb236e86347af3e46b519c95e41b124 | 0 | 36 | 0 | 0 | 0 | 0 |  |
| db51fc4976e3e94aa6ea7dc40fb62f3a | 0 | 36 | 0 | 0 | 0 | 0 | s__uncultured_Verrucomicrobia |
| 170caf383789ab5640fee8c0b5e516f9 | 0 | 0 | 0 | 0 | 0 | 35 |  |
| b632afe835fe2cf13ea433ba9798bbec | 0 | 0 | 35 | 0 | 0 | 0 |  |
| 774aee1a9dcd409268879da3e2377984 | 0 | 0 | 35 | 0 | 0 | 0 |  |
| f77fc3695c851de86f7bf796dbdf46ef | 0 | 0 | 35 | 0 | 0 | 0 | s__Nocardioides_furvisabuli |
| 0b03d68b75c46f3a82614a0469c016b4 | 35 | 0 | 0 | 0 | 0 | 0 | s__uncultured_Verrucomicrobiales |
| aca56c04e959495a1bddfb22841fd474 | 35 | 0 | 0 | 0 | 0 | 0 |  |
| 6fc4344edd5af4ff589ca5479512ef98 | 0 | 0 | 0 | 0 | 35 | 0 |  |
| eb2e7db9525da686e72004fc0164a66f | 0 | 0 | 34 | 0 | 0 | 0 |  |
| 265bb8f30a62f9239a39be5eed031404 | 34 | 0 | 0 | 0 | 0 | 0 | s__uncultured_soil |
| c9e939ff9dda4df762a0244439655424 | 34 | 0 | 0 | 0 | 0 | 0 |  |
| b04f6e1e206a0b97ba4feec002036275 | 0 | 0 | 0 | 0 | 34 | 0 | s__Microvirga_flocculans |
| 2c5c16380592aceb44b5e291195b67a3 | 0 | 0 | 0 | 0 | 34 | 0 |  |
| acb2053362351a35e905866dd7c99f02 | 0 | 0 | 0 | 34 | 0 | 0 | s__Bacillus_alkalitelluris |
| 1090f60c754803879cd1df137ff19e76 | 0 | 0 | 0 | 34 | 0 | 0 | s__uncultured_Thermomonas |
| 8dd731d5b74745678feb5b86a63e82a3 | 0 | 34 | 0 | 0 | 0 | 0 |  |
| b1b3e0fc7669e51277d57b143d3d3c02 | 0 | 34 | 0 | 0 | 0 | 0 |  |
| 24cfe1660f834297e06578c15f588ab9 | 0 | 0 | 0 | 0 | 0 | 33 |  |
| 034dfdc73e9c0b0edc318a327c8bceb2 | 33 | 0 | 0 | 0 | 0 | 0 | s__uncultured_Bacteroidetes |
| 543a11f78a2f72ed1fcc8d0890ba8ac6 | 0 | 0 | 0 | 0 | 33 | 0 |  |
| dd7739455eff662ce752fb80702ef215 | 0 | 0 | 0 | 0 | 33 | 0 |  |
| 34a9f6b7115bb84bf00bcfe5c2e0b7c4 | 0 | 0 | 0 | 33 | 0 | 0 | s__uncultured_Verrucomicrobia |
| 6eee36e8f353332a1e42044b105ddf72 | 0 | 0 | 0 | 33 | 0 | 0 | s__uncultured_bacterium |
| 49e83ff55ff07a1944e6211476fdb517 | 0 | 0 | 0 | 33 | 0 | 0 |  |
| e2626cf325151b8d2f22dece42f78dc9 | 0 | 0 | 0 | 33 | 0 | 0 | s__uncultured_bacterium |
| 755efc4dbe2514637e77a75400c17809 | 32 | 0 | 0 | 0 | 0 | 0 |  |
| 5c160e1019c3a98ed9b97ee361895a61 | 0 | 0 | 0 | 0 | 32 | 0 |  |
| 8fa382c6f5deb5a0c1f8f2c68dec4ac2 | 0 | 32 | 0 | 0 | 0 | 0 |  |
| 1de256e9c3573c60e6f5a0983152d562 | 0 | 0 | 0 | 0 | 0 | 31 |  |
| 08d1893ac3c7d25d9ffd2133d15a3790 | 0 | 0 | 0 | 0 | 0 | 31 |  |
| 21816d04512cf74158fa9970e1d3df25 | 0 | 0 | 0 | 0 | 0 | 31 |  |
| bce37c7518e1ff8276ed3fd44a787004 | 0 | 0 | 31 | 0 | 0 | 0 | s__Bacillus_alkalitelluris |
| 46e1a82e908d7499e2341b41b71e7bf1 | 31 | 0 | 0 | 0 | 0 | 0 | s__Acidobacteria_bacterium |
| b1e8beaa3165b55ccc6ac019bccdb8a9 | 31 | 0 | 0 | 0 | 0 | 0 | s__uncultured_Ohtaekwangia |
| 559c9d474c39ea16b9e4a187ef5eac21 | 31 | 0 | 0 | 0 | 0 | 0 | s__uncultured_bacterium |
| 8b93583f18d59b67ef6a986f9019a26d | 0 | 0 | 0 | 0 | 31 | 0 | s__uncultured_Acidimicrobiia |
| 1c83d8bce17796730e8704a305bbd891 | 0 | 0 | 0 | 0 | 31 | 0 |  |
| 5bf35e8f563416a304847154f1a806cc | 0 | 0 | 0 | 0 | 31 | 0 |  |
| f68da4197f91d789c82fde6c954df425 | 0 | 0 | 0 | 31 | 0 | 0 | s__unidentified |
| 0e1fa9096fc083611be72d8482aa24c3 | 0 | 0 | 0 | 31 | 0 | 0 | s__uncultured_Alphaproteobacteria |
| 641e3a63e57e31d61c6d0cf4b2e4440b | 0 | 14 | 0 | 17 | 0 | 0 | s__uncultured_Flavobacteriaceae |
| 23127bdfd7b76580805c24545c5a3b77 | 0 | 0 | 30 | 0 | 0 | 0 |  |
| 3af1b88700b3da7372e9e02541d0d7d0 | 0 | 0 | 30 | 0 | 0 | 0 | s__uncultured_Planctomycetales |
| f4b7a305ddeca705102c5e35f89dfac4 | 0 | 0 | 0 | 0 | 30 | 0 |  |
| 2323465fcacd86faa676ac45244b67cc | 0 | 0 | 0 | 0 | 30 | 0 | s__Lysobacter_terricola |
| 99041f5d90d86e8559eb97416b414f79 | 0 | 30 | 0 | 0 | 0 | 0 |  |
| 1268edecb82c2ef1cb680f19d8e4e1f7 | 0 | 30 | 0 | 0 | 0 | 0 | s__uncultured_Firmicutes |
| c79557c032e5a24b7fa53b3c493899e7 | 0 | 30 | 0 | 0 | 0 | 0 | s__Sphingomonas_sanxanigenens |
| ac8d95ca086ff7940660678863e97172 | 0 | 30 | 0 | 0 | 0 | 0 |  |
| 924fcac7008ef08f89986b14715d1e75 | 0 | 30 | 0 | 0 | 0 | 0 | s__uncultured_delta |
| 5181dd6d8354a07d1cd18101baf44d37 | 0 | 30 | 0 | 0 | 0 | 0 | s__Sphingomonas_sanxanigenens |
| 82a0fcad6c8f781b816eb4b561985d7b | 0 | 0 | 0 | 0 | 0 | 29 | s__Nocardioides_furvisabuli |
| 62b24d99d6655d8840d09da639ced042 | 0 | 0 | 0 | 0 | 0 | 29 |  |
| 52e4678c76d2e016dfc7372e07889811 | 0 | 0 | 29 | 0 | 0 | 0 | s__Arenimonas_daejeonensis |
| e7837779dab545e3ee2169fb54bc3335 | 29 | 0 | 0 | 0 | 0 | 0 |  |
| 6bd0b6bb01899a839510712b79584b7d | 29 | 0 | 0 | 0 | 0 | 0 | s__uncultured_bacterium |
| 24b90cee5e9b7af86bada395225afce7 | 29 | 0 | 0 | 0 | 0 | 0 |  |
| 46c2eafc9dcf9bdd115fca6a5be09d20 | 0 | 0 | 0 | 0 | 29 | 0 |  |
| 1b5d14c5063ea10e442b1670d1798f0c | 0 | 0 | 0 | 29 | 0 | 0 |  |
| 491ead851fd8a9f72b359b90a24bd4d2 | 0 | 0 | 0 | 29 | 0 | 0 |  |
| e4d4f08edf396a75876ad6a9f3c9755b | 0 | 29 | 0 | 0 | 0 | 0 |  |
| 0134bcba4f5411c7a3c86c097d6598d9 | 0 | 0 | 28 | 0 | 0 | 0 |  |
| 73f670089aa9d6e14b5613a397bb67ab | 0 | 0 | 28 | 0 | 0 | 0 |  |
| 31a62a20f8841a81874ed29d49d4f529 | 0 | 28 | 0 | 0 | 0 | 0 | s__uncultured_soil |
| 2def7f461f4234b2d503a4cc44d3ee08 | 0 | 0 | 0 | 0 | 0 | 27 | s__uncultured_soil |
| 66a62b47da8042a54d3b05eb8c01e800 | 0 | 0 | 0 | 0 | 0 | 27 | s__Archangium_gephyra |
| 847566bbbb0a908f259f31e1eb79b8df | 0 | 0 | 0 | 0 | 0 | 27 |  |
| 610b667f9ce60e516076fd17e4effbb3 | 27 | 0 | 0 | 0 | 0 | 0 |  |
| e21dd49b4b82449e59d6a8fe919dea76 | 0 | 0 | 0 | 0 | 27 | 0 |  |
| e8b703a3de294d1864caf0fa0146a6fd | 0 | 27 | 0 | 0 | 0 | 0 | s__uncultured_Planctomycetaceae |
| 7f36a6cc5990426dad8eaf7fe6385c22 | 0 | 0 | 0 | 0 | 0 | 26 |  |
| bcea5501fcdfd3802ab2c9e5baa01eb7 | 0 | 0 | 0 | 0 | 0 | 26 | s__uncultured_Bacteroidetes |
| 6dbc1dd1e68d34eb7eb28aabad5411dc | 0 | 0 | 26 | 0 | 0 | 0 |  |
| 7fc345c0158169106bfc1a0ffa1b9c73 | 0 | 0 | 0 | 0 | 26 | 0 |  |
| 4448f112d492e013511502369f1413f1 | 0 | 0 | 0 | 26 | 0 | 0 |  |
| dcab2b04386c6da827709bc763615323 | 0 | 26 | 0 | 0 | 0 | 0 | s__Devosia_neptuniae |
| 6f211326358e32ca9544ac066d9a8045 | 0 | 26 | 0 | 0 | 0 | 0 |  |
| 20ff930548d9a9cfc5281994d6901c33 | 0 | 26 | 0 | 0 | 0 | 0 |  |
| 7d7694941b762230f3e33b007385c9a7 | 0 | 26 | 0 | 0 | 0 | 0 |  |
| 2a17244bb5bdeefa6364dfb29877f17f | 0 | 26 | 0 | 0 | 0 | 0 |  |
| 3dd06d1624de65528644e48e8f645cdb | 0 | 0 | 0 | 0 | 0 | 25 | s__uncultured_Actinomycetales |
| 4e9b1333524d5c49fd28b3413ce34316 | 0 | 0 | 0 | 0 | 0 | 25 |  |
| 94b3df234d04155d10de52611516105d | 0 | 0 | 0 | 0 | 0 | 25 | s__uncultured_Acidobacteriales |
| 829d4770bd0394ef2cb7e98acb47acd9 | 0 | 0 | 25 | 0 | 0 | 0 |  |
| c336bd6fe2a43819bdb2608a5b5b2cec | 25 | 0 | 0 | 0 | 0 | 0 | s__uncultured_bacterium |
| 9562893e53bb65158bdc529582652ac0 | 25 | 0 | 0 | 0 | 0 | 0 |  |
| a9d08b2a939918750c52bec0f47b70fe | 0 | 0 | 0 | 0 | 25 | 0 | s__uncultured_Planctomycetia |
| bdfef8c6ab3509b74c3460e498d65943 | 0 | 0 | 0 | 0 | 25 | 0 | s__metagenome |
| 3896a18e365af936d25fbbadf18841c6 | 0 | 0 | 0 | 25 | 0 | 0 |  |
| ac7507ef36d389ed090d24725b1b163d | 0 | 0 | 0 | 25 | 0 | 0 | s__uncultured_bacterium |
| 8023e69a1ccc961175d332b4f9fa4708 | 0 | 0 | 0 | 25 | 0 | 0 | s__uncultured_bacterium |
| c886f2afcbbd14250b7338bce771e2de | 0 | 25 | 0 | 0 | 0 | 0 | s__uncultured_Alphaproteobacteria |
| 790b9949db8e07ea9a217703df7bc4fe | 0 | 0 | 0 | 0 | 0 | 24 |  |
| a0e85027f8b357ee5d79fa507a02210a | 0 | 0 | 0 | 0 | 0 | 24 | s__uncultured_soil |
| 427f47e24def85712bb922f6e82a1fa1 | 0 | 0 | 0 | 0 | 0 | 24 |  |
| 0aecdd6f98633745571525a08df22bf7 | 0 | 0 | 0 | 0 | 0 | 24 |  |
| dff01aa63991995b4c1f2e25292c57c9 | 0 | 0 | 24 | 0 | 0 | 0 |  |
| 2323ff19cecafd863323a38740113668 | 0 | 0 | 24 | 0 | 0 | 0 | s__uncultured_Alphaproteobacteria |
| b0970fe24d0bebbef19ac4f4e4c228d8 | 0 | 0 | 24 | 0 | 0 | 0 |  |
| 34a2705f400c2c8d005840544cc9c210 | 0 | 0 | 24 | 0 | 0 | 0 | s__uncultured_bacterium |
| 76408052f8a04b57e4dc368560a79d3d | 24 | 0 | 0 | 0 | 0 | 0 |  |
| 1621223b336c4807be9d68a70e230049 | 24 | 0 | 0 | 0 | 0 | 0 | s__Parachlamydia_sp. |
| b3544bba9977a858f70fc6abfa9826c3 | 24 | 0 | 0 | 0 | 0 | 0 | s__uncultured_Pirellula |
| 68503a51bc6d9dd22d7db16e68241526 | 24 | 0 | 0 | 0 | 0 | 0 |  |
| 5434f86c7eb1a2640de61caf3a084234 | 0 | 0 | 0 | 0 | 24 | 0 |  |
| 9f604fdda3a97f8acb9b789d7cdea9ec | 0 | 0 | 0 | 0 | 24 | 0 |  |
| 587e5b7994592ccaecd44753ee6691a5 | 0 | 0 | 0 | 0 | 24 | 0 |  |
| 46260265fb48a9c61904e45cf2b2e7b2 | 0 | 0 | 0 | 0 | 24 | 0 |  |
| cc9a6d389df4a5f519b1c34ac76e8e81 | 0 | 0 | 0 | 0 | 24 | 0 | s__uncultured_Alphaproteobacteria |
| bb1b32192f7bdcf361b97cf603e8c85e | 0 | 0 | 0 | 24 | 0 | 0 | s__metagenome |
| e76944647d1ea7ce7826f3ff8956aa70 | 0 | 0 | 0 | 24 | 0 | 0 | s__uncultured_bacterium |
| 5fc69cc2919de3001b06518eb7ca8ff3 | 0 | 24 | 0 | 0 | 0 | 0 | s__Microbulbifer_okinawensis |
| bde3f6102358e264e61c6864e2aa0a9a | 0 | 24 | 0 | 0 | 0 | 0 |  |
| 06ee736d515a0251d1be42669265d85b | 0 | 0 | 0 | 0 | 0 | 23 |  |
| e6085f501f8377e4fdad58404346d070 | 0 | 0 | 0 | 0 | 0 | 23 |  |
| 65d18a46c90011d9fcfc1aff637ce9c4 | 0 | 0 | 23 | 0 | 0 | 0 | s__uncultured_bacterium |
| 27e6975adf1fb17b02386e3c1be90733 | 23 | 0 | 0 | 0 | 0 | 0 | s__uncultured_bacterium |
| fb634d0909278acb544d523ebcf4db89 | 0 | 0 | 0 | 0 | 23 | 0 | s__Cyanobacteria/Melainabacteria_group |
| 30bddedb66a118ac3121911289669cd0 | 0 | 0 | 0 | 23 | 0 | 0 | s__metagenome |
| 397dc9b06e12ec4d21ad1ef638e66f65 | 0 | 0 | 0 | 23 | 0 | 0 |  |
| 62409c5f703b30fb8aea2f63fd48646f | 0 | 23 | 0 | 0 | 0 | 0 | s__uncultured_Planctomycetales |
| a55ae1c48c673bc3e466cdb9ae039020 | 0 | 23 | 0 | 0 | 0 | 0 | s__uncultured_Bacteroidetes |
| d4baf26d43d51fc82e6398f1c9161263 | 0 | 0 | 0 | 0 | 0 | 22 |  |
| e72437102356def129a86230f0ac66b2 | 0 | 0 | 0 | 0 | 0 | 22 |  |
| 92d90afd41a26b72a91675496af776f9 | 0 | 0 | 22 | 0 | 0 | 0 | s__Planctomyces_sp. |
| 4f8b33cba71b62998227b3370ee7e5ff | 0 | 0 | 0 | 0 | 22 | 0 |  |
| 1180c1d797dcbd52f7273cfe2fbbf28a | 0 | 0 | 0 | 0 | 22 | 0 |  |
| d200f2bb50fe966f352a35e73bb0a947 | 0 | 0 | 0 | 0 | 22 | 0 |  |
| a05a5b64dd5784c6f52e27f3d4e26956 | 0 | 0 | 0 | 0 | 22 | 0 | s__Flavisolibacter_ginsengisoli |
| a03bbc6e4bf43297cf251c93761f2fbc | 0 | 0 | 0 | 22 | 0 | 0 | s__uncultured_Actinomycetales |
| 2451089bc93bb16103b18fff8f8a8419 | 0 | 22 | 0 | 0 | 0 | 0 |  |
| b2b09ee44d97bba051bd1cf3721d0c57 | 21 | 0 | 0 | 0 | 0 | 0 | s__Aquimonas_sp. |
| 86d253a47ceb6163b16ad19420e51816 | 21 | 0 | 0 | 0 | 0 | 0 | s__Aquimonas_sp. |
| a50e8a330efcc4649c11d77ed1879199 | 21 | 0 | 0 | 0 | 0 | 0 | s__uncultured_bacterium |
| d693f9bba7393cda1f039262c77f8c8a | 0 | 0 | 0 | 0 | 21 | 0 |  |
| e81c107a8e15c3ce93290f54388aa0f1 | 0 | 0 | 0 | 0 | 21 | 0 | s__uncultured_Planctomycetaceae |
| 2119d9b3793b88e0461c12c6acc47f8f | 0 | 21 | 0 | 0 | 0 | 0 |  |
| 5fd7d1cdee63c03d7f4e15d73cd6122f | 0 | 0 | 0 | 0 | 0 | 20 |  |
| fdb47e07e2be193f994b5d664998f37f | 0 | 0 | 0 | 0 | 0 | 20 | s__uncultured_Firmicutes |
| 3c8ed7edc98da6ea8d5e03b3ff56d52c | 0 | 0 | 0 | 0 | 0 | 20 | s__uncultured_Acidobacteriaceae |
| 25d650e01ee7406487f995cc005abcf2 | 20 | 0 | 0 | 0 | 0 | 0 |  |
| b60aeaf5c7da85d5e960cc26ba82b914 | 20 | 0 | 0 | 0 | 0 | 0 |  |
| ee49f673f6f4f4acaad91c4847a1c5b9 | 0 | 0 | 0 | 0 | 20 | 0 | s__Luteimonas_marina |
| dc999dc6ed84b55cb23c9180bd7a2f42 | 0 | 0 | 0 | 0 | 20 | 0 |  |
| ff556263ff71cc2add4f8e1e9258f295 | 0 | 0 | 0 | 0 | 20 | 0 | s__uncultured_bacterium |
| a203fb05a05d925f83ba713f79d49b4a | 0 | 0 | 0 | 20 | 0 | 0 |  |
| c0bbcdd1d710c9140e732cc6aac6ef1e | 0 | 20 | 0 | 0 | 0 | 0 | s__uncultured_Candidatus |
| 8359d4af19abed779829233c8bf68fcd | 0 | 0 | 0 | 0 | 0 | 19 | s__metagenome |
| f7ece90a79c6595a9c91e2170760b1f9 | 0 | 0 | 19 | 0 | 0 | 0 |  |
| 4b8cc1812b7f10be92e55b95d2cbd6b0 | 0 | 0 | 19 | 0 | 0 | 0 | s__metagenome |
| 246f0139bfc416ffda30ff2360964775 | 0 | 0 | 0 | 0 | 19 | 0 | s__metagenome |
| 8fb07b4abdac176d698c05e20d46ee36 | 0 | 0 | 0 | 0 | 19 | 0 |  |
| 9425fc85500288a91c7bf398b5adb178 | 0 | 0 | 0 | 0 | 19 | 0 |  |
| 67774cf10498c6d09e1b166422f05f78 | 0 | 0 | 0 | 0 | 0 | 18 | s__Pedobacter_borealis |
| 4bd9fed54c374ed8a8093577a8954273 | 0 | 0 | 18 | 0 | 0 | 0 | s__uncultured_Acidobacteriales |
| df545a01b5dd5e46dc83744784306616 | 0 | 0 | 18 | 0 | 0 | 0 | s__uncultured_sludge |
| e46b204dc7aa9755c1b772175874cc8f | 0 | 0 | 18 | 0 | 0 | 0 |  |
| a50eb30bb40407940dafa7edf3cd4928 | 18 | 0 | 0 | 0 | 0 | 0 |  |
| ee7563698b07041ea57e4c17f81c6898 | 18 | 0 | 0 | 0 | 0 | 0 | s__uncultured_Planctomycetales |
| 3445b76d5dd7f1a12df78c13e479138a | 18 | 0 | 0 | 0 | 0 | 0 |  |
| 0f9b4de595ccb42696dffa72979f06d1 | 0 | 0 | 0 | 0 | 18 | 0 | s__uncultured_soil |
| 3f7a983c9c0beef7c6b2cbf768b51bb2 | 0 | 0 | 0 | 0 | 18 | 0 |  |
| 8936a3f9273a8b5b98d18c587dc41fb7 | 0 | 0 | 0 | 0 | 18 | 0 |  |
| c54c83158d33abd9ecff8afef9e2e0a5 | 0 | 0 | 0 | 0 | 18 | 0 |  |
| f84b77af824e7c5ab7b3612147b9e903 | 0 | 0 | 0 | 18 | 0 | 0 | s__Roseimicrobium_gellanilyticum |
| 3dcf81beeb354d338448420b2f001df6 | 0 | 0 | 0 | 18 | 0 | 0 |  |
| 2945a2a6ef818028eec33a73aeee6a40 | 0 | 0 | 0 | 18 | 0 | 0 |  |
| 332fe6563f0300c5fcba135371dabfac | 0 | 18 | 0 | 0 | 0 | 0 |  |
| e6039e98b945ef0bcf9fd6638efc3c47 | 0 | 18 | 0 | 0 | 0 | 0 |  |
| f013df3b9936e08468c30dc6f5035ce2 | 0 | 18 | 0 | 0 | 0 | 0 |  |
| 26a7f7c8ffbc4cd2ee8ffc6a74429f46 | 0 | 0 | 0 | 0 | 0 | 17 |  |
| 4f42410ac288cb3c61aeb996b9cc7c2a | 0 | 0 | 0 | 0 | 0 | 17 | s__Steroidobacter_sp. |
| 010418f9d561452f9484801ec822cf04 | 0 | 0 | 0 | 0 | 0 | 17 |  |
| cd927f0f8ae3a6510b8e77c3dce0b0c6 | 0 | 0 | 0 | 0 | 0 | 17 |  |
| 26d400e58473ceac6280e9dffd40f075 | 0 | 0 | 0 | 0 | 0 | 17 |  |
| f204eb2f48f337cfcf72756971ae1775 | 0 | 0 | 17 | 0 | 0 | 0 |  |
| 101f7b7479ebca22c0f286a481a64a1a | 0 | 0 | 17 | 0 | 0 | 0 |  |
| c31ae0a05d514303fd922d7086a9c879 | 17 | 0 | 0 | 0 | 0 | 0 | s__metagenome |
| b1e73ab8bcaafebc13ff331f21be6386 | 0 | 0 | 0 | 0 | 17 | 0 | s__uncultured_bacterium |
| 52fd4822bdc4818c84859522d756f7ff | 0 | 0 | 0 | 17 | 0 | 0 |  |
| 105eac749ebb78e1c896f3b3f4ddc8c6 | 0 | 0 | 0 | 17 | 0 | 0 | s__uncultured_soil |
| 100ebe062087a18dfff8b79922252462 | 0 | 17 | 0 | 0 | 0 | 0 |  |
| edb3f486cc685635a52b8bd9323504db | 0 | 0 | 0 | 0 | 0 | 16 | s__Algoriphagus_terrigena |
| e315cc5585a5a19786d94ac253228437 | 0 | 0 | 0 | 0 | 0 | 16 | s__uncultured_soil |
| 55e039959b48b22f9b18a85498a37361 | 0 | 0 | 16 | 0 | 0 | 0 | s__metagenome |
| dcb3d5d3271e247e99bf00f995723b43 | 16 | 0 | 0 | 0 | 0 | 0 | s__Planctomyces_sp. |
| e335b3cb6a0a7ec66a1e400493bc1b64 | 16 | 0 | 0 | 0 | 0 | 0 | s__uncultured_bacterium |
| e314c8618d24d8f6c1bab506c3ba5a1f | 0 | 0 | 0 | 0 | 16 | 0 | s__uncultured_Planctomycetaceae |
| 812a36601863be8b4bd517482121faf0 | 0 | 16 | 0 | 0 | 0 | 0 | s__uncultured_soil |
| 367f28a13f40e5b23c6d47d83251245c | 0 | 16 | 0 | 0 | 0 | 0 |  |
| 21e85b70562dc5b9a8b8d2c755b36ecf | 0 | 16 | 0 | 0 | 0 | 0 |  |
| 8efdf5a3efad6550d75f8b3bd213a3ac | 0 | 16 | 0 | 0 | 0 | 0 | s__uncultured_Flavobacteriaceae |
| 35f4d014228747cf07bf9ab17dd2261a | 0 | 16 | 0 | 0 | 0 | 0 | s__uncultured_bacterium |
| 2e4366b720a1bdb8c9c9f3dd8565d227 | 0 | 0 | 0 | 0 | 0 | 15 |  |
| 0d169753f7ed6aa243a8156cab2205d3 | 0 | 0 | 15 | 0 | 0 | 0 | s__uncultured_bacterium |
| b33c80424116e05b1db25f27012b2a78 | 0 | 0 | 15 | 0 | 0 | 0 | s__uncultured_Verrucomicrobiales |
| e2bdc59b3c20402b928af2b490b08e89 | 0 | 0 | 15 | 0 | 0 | 0 | s__uncultured_bacterium |
| ff1d7231f3e174029688301e0d223822 | 15 | 0 | 0 | 0 | 0 | 0 | s__uncultured_bacterium |
| 270d58af18a822ef1e42ad4a88ec6b98 | 15 | 0 | 0 | 0 | 0 | 0 |  |
| 7fd193ff4dfdccdd00382b9e2b303b27 | 15 | 0 | 0 | 0 | 0 | 0 | s__uncultured_Acidobacteriales |
| 1b50a89f32973fce5f352fb5d02bbccc | 0 | 0 | 0 | 0 | 15 | 0 | s__metagenome |
| 5fc754b2dc6b257877cb486d06956b0e | 0 | 0 | 0 | 15 | 0 | 0 | s__uncultured_bacterium |
| a7222e7811b9a472945ed684283ddcd8 | 0 | 0 | 0 | 15 | 0 | 0 |  |
| bbd45d19bdc5136610f2514f29c02bc7 | 0 | 0 | 0 | 15 | 0 | 0 | s__uncultured_Chloroflexi |
| 41a3cc11486cca0b5ad3f5430ff23d48 | 0 | 15 | 0 | 0 | 0 | 0 | s__uncultured_bacterium |
| ea253eb2d1beac7250007c4f92d87be4 | 0 | 15 | 0 | 0 | 0 | 0 |  |
| 880e718d096c25b9f82386278a2a2bc9 | 0 | 0 | 0 | 0 | 0 | 14 |  |
| 1927b5d9e71898b51b11ac2c3e29c4c7 | 0 | 0 | 0 | 0 | 0 | 14 | s__uncultured_soil |
| 07e3ca0f87ea6586674fe3e98b137685 | 0 | 0 | 14 | 0 | 0 | 0 |  |
| 332bf2747e2b511260b9d3563e238d57 | 0 | 0 | 14 | 0 | 0 | 0 | s__uncultured_gamma |
| b3f10179e4170c7af41b191e2667eeb6 | 0 | 0 | 0 | 14 | 0 | 0 |  |
| cf16c715abc409cad2c34bab2359182a | 0 | 14 | 0 | 0 | 0 | 0 |  |
| 40eccc8db51de1d4570fd50a02cff756 | 0 | 14 | 0 | 0 | 0 | 0 |  |
| 8d9f392d4b6c76c2150f55bbf4b01c34 | 0 | 14 | 0 | 0 | 0 | 0 |  |
| 1283856ec58d752e331d2bad375ded28 | 0 | 14 | 0 | 0 | 0 | 0 |  |
| 9dce8a04a834ff36ce9377f523863a40 | 0 | 0 | 0 | 0 | 0 | 13 | s__Oceanobacillus_picturae |
| 8c4c9fe80284ea37fe9aa12c4559ef60 | 0 | 0 | 0 | 0 | 0 | 13 | s__Olivibacter_soli |
| 88fb9d774927d0cc80b6e975e1b71b0a | 0 | 0 | 13 | 0 | 0 | 0 | s__uncultured_bacterium |
| 4ba984bffd49e433c61b0bc6a986b6a1 | 13 | 0 | 0 | 0 | 0 | 0 |  |
| f1a3a5f386cf831c4c048ef321f2bfe0 | 13 | 0 | 0 | 0 | 0 | 0 | s__uncultured_bacterium |
| 0e4597d8abcc317f3964e18978dce264 | 0 | 0 | 0 | 0 | 13 | 0 | s__uncultured_bacterium |
| 066c7ee45449a4dbd8ff34a2055937ce | 0 | 0 | 0 | 13 | 0 | 0 |  |
| 9be49d92463136ff5ce9cfdc134fac8e | 0 | 13 | 0 | 0 | 0 | 0 |  |
| 772ca3e860a057d6a32032d904c42923 | 0 | 13 | 0 | 0 | 0 | 0 |  |
| 9bac1d16797047c9325ee00fde4c1bb1 | 0 | 13 | 0 | 0 | 0 | 0 | s__uncultured_planctomycete |
| dc2a85ee8dadfe82d826f98eca70d5c7 | 0 | 13 | 0 | 0 | 0 | 0 |  |
| fb31a154780fbea0cb713a61ec49c69c | 0 | 0 | 12 | 0 | 0 | 0 |  |
| 31248b502232034ee44a44e84444e8af | 0 | 0 | 12 | 0 | 0 | 0 |  |
| 6897fb6f4f84aed3631c3e4a020809a8 | 0 | 0 | 0 | 0 | 12 | 0 |  |
| 41dba7d9a4a841cfdf1412af0a5512c9 | 0 | 0 | 0 | 12 | 0 | 0 |  |
| 92e671610cf47d461d679c1d3b8a34de | 0 | 0 | 0 | 12 | 0 | 0 | s__uncultured_bacterium |
| ca191acfe0e8f6bb06353a64760a30bd | 0 | 0 | 0 | 12 | 0 | 0 |  |
| e2775983ba4a1d2785703c5d6acde812 | 0 | 12 | 0 | 0 | 0 | 0 |  |
| 74a45d346138602e5a22a50e25b73d30 | 0 | 12 | 0 | 0 | 0 | 0 | s__uncultured_Planctomycetales |
| 4d7a26b612c3d138f213cb3eec1cd060 | 0 | 12 | 0 | 0 | 0 | 0 |  |
| 20eb988269bdf0bece3242710d8d1c68 | 0 | 12 | 0 | 0 | 0 | 0 |  |
| a1e02f14d9fcbd9ececcfd385d0a55aa | 0 | 12 | 0 | 0 | 0 | 0 | s__Parapedobacter_pyrenivorans |
| db026d48b2d0049256cd127189afe448 | 11 | 0 | 0 | 0 | 0 | 0 | s__uncultured_bacterium |
| 84601cce08c450864e0f66158d6f8363 | 11 | 0 | 0 | 0 | 0 | 0 | s__uncultured_gamma |
| 1d6d4ad82c33e9ac24aa5057d927b352 | 11 | 0 | 0 | 0 | 0 | 0 | s__uncultured_bacterium |
| f1f1eff75df909793509a050d21cce3f | 11 | 0 | 0 | 0 | 0 | 0 |  |
| 167281d72c4b4400e8cf497f18fe572f | 0 | 0 | 0 | 0 | 11 | 0 | s__uncultured_bacterium |
| f45339b9d4b7c48cf1b62d2fbcc81759 | 0 | 11 | 0 | 0 | 0 | 0 | s__uncultured_bacterium |
| b006af854d60e5bd6d0db786b1197206 | 0 | 11 | 0 | 0 | 0 | 0 | s__uncultured_bacterium |
| e0ebbf657796854ca7758213025ac8f5 | 0 | 11 | 0 | 0 | 0 | 0 | s__uncultured_bacterium |
| 7e3509500cb10548fc693b5b924d8be4 | 0 | 0 | 0 | 0 | 0 | 10 | s__bacterium_Ellin507 |
| c9c363a2a6adad464df01a39cbd5e23b | 0 | 0 | 10 | 0 | 0 | 0 |  |
| b7480aa98cc5e02b1a174a512004e353 | 0 | 0 | 10 | 0 | 0 | 0 |  |
| 4a04fd94e9565ec405142adb27447dc7 | 0 | 0 | 10 | 0 | 0 | 0 |  |
| ace004bd4f7fd5d3ff8eac79c1296dfe | 0 | 0 | 10 | 0 | 0 | 0 |  |
| 03b62b009d478c7b1455516f77031d70 | 0 | 0 | 10 | 0 | 0 | 0 | s__uncultured_Chloroflexi |
| dfd119603812fb281d55da5d36505c2d | 0 | 0 | 10 | 0 | 0 | 0 |  |
| 30d29e6b524a8df4d7c5a6aa80923f93 | 0 | 0 | 0 | 0 | 10 | 0 | s__uncultured_prokaryote |
| a56fbe518e17fd3751ef0b25c341d608 | 0 | 0 | 0 | 0 | 10 | 0 |  |
| 7bcff0a88e20d3f6ff78dbdfad721c49 | 0 | 0 | 0 | 0 | 10 | 0 |  |
| f537a8d618de01591bee605e876c4e94 | 0 | 10 | 0 | 0 | 0 | 0 | s__Niastella_sp. |
| 242b8cd643c752915a844fefcc32f5c7 | 0 | 10 | 0 | 0 | 0 | 0 | s__uncultured_bacterium |
| 56805ee4ecb80dec9676c3d3c1bf2616 | 0 | 10 | 0 | 0 | 0 | 0 | s__uncultured_Candidatus |
| e99274ddf9a7df3475fcfc66f7a7ce2c | 0 | 10 | 0 | 0 | 0 | 0 |  |
| ec227142a0e52b0e0f49fc85e2e52aa1 | 0 | 0 | 0 | 0 | 0 | 9 | s__uncultured_bacterium |
| 0d8655a795da36529e66c9dab096c0d1 | 0 | 0 | 9 | 0 | 0 | 0 | s__uncultured_bacterium |
| 97a5bc3bc1ae318bb32767f551da581f | 9 | 0 | 0 | 0 | 0 | 0 | s__Arenimonas_daejeonensis |
| 1522b1184cffce9b32216718df11235b | 9 | 0 | 0 | 0 | 0 | 0 | s__Mycoavidus_cysteinexigens |
| 38eb3e0dc549a6885b444c7e787552f4 | 0 | 0 | 0 | 0 | 9 | 0 |  |
| 7f395137f5d20ba75381c0c34e9bb9ea | 0 | 0 | 0 | 0 | 9 | 0 | s__uncultured_bacterium |
| b6c21a68f3cbd2902d84c0f74f286d6d | 0 | 0 | 0 | 0 | 9 | 0 |  |
| bdff6cc635ef34f0da5fe791c14c6847 | 0 | 0 | 0 | 0 | 9 | 0 |  |
| f5ae2ede5dbbf23fd0b6c12893752e0a | 0 | 0 | 0 | 0 | 9 | 0 |  |
| 26da850eb5152c88958b208a7761457e | 0 | 0 | 0 | 9 | 0 | 0 | s__uncultured_bacterium |
| c181dd76340dbf2d2e699aa09d5f0f39 | 0 | 9 | 0 | 0 | 0 | 0 |  |
| ba30214498972e29794c0f270e175683 | 0 | 9 | 0 | 0 | 0 | 0 |  |
| 5f230246559c73ad08aa418567235286 | 0 | 9 | 0 | 0 | 0 | 0 | s__uncultured_bacterium |
| 8cf7a4868571bc8b6911fdd3099ad29e | 0 | 9 | 0 | 0 | 0 | 0 | s__uncultured_Acidimicrobiales |
| 6c4a8c0a402494b57fb8e50cb5cade1c | 0 | 9 | 0 | 0 | 0 | 0 | s__uncultured_Actinomycetales |
| 7f1e188e74cf8cf746d2cfc3d965c6e5 | 0 | 0 | 0 | 0 | 0 | 8 | s__Cellvibrio_diazotrophicus |
| e70a2fc43ca0f9bbd1308b97e20bfdf6 | 0 | 0 | 0 | 0 | 0 | 8 | s__Turicibacter_sp. |
| a961ff9d56e4915e56b960bc9f84a52e | 0 | 0 | 8 | 0 | 0 | 0 | s__uncultured_Chlorobiales |
| d48d978f6db7a6502009ad9ff87ffe39 | 0 | 0 | 8 | 0 | 0 | 0 | s__uncultured_cyanobacterium |
| a974be239bcb6bc02833d70bea04f5f6 | 0 | 0 | 8 | 0 | 0 | 0 |  |
| 7ac7fca1f53435609e052db0b088ea89 | 0 | 0 | 8 | 0 | 0 | 0 | s__Roseomonas_lacus |
| d20bd97441fde75b23688751861e92f6 | 0 | 0 | 0 | 0 | 8 | 0 |  |
| 545eece67c3822f733405fdde9e84273 | 0 | 0 | 0 | 0 | 8 | 0 | s__uncultured_Myxococcales |
| e6341748e9776189cfa076e256c08235 | 0 | 0 | 0 | 0 | 8 | 0 | s__uncultured_bacterium |
| d21b9a5373cda4c6d19ef5161fef42d0 | 0 | 0 | 0 | 8 | 0 | 0 | s__uncultured_Bacteroidetes |
| 65083ca473fe51e7ab6f99069f25971c | 0 | 0 | 0 | 8 | 0 | 0 |  |
| c50577f4dd590e7e8e62471221408659 | 0 | 8 | 0 | 0 | 0 | 0 |  |
| 0a57a2658470ce821a59929d22d5dd9d | 7 | 0 | 0 | 0 | 0 | 0 | s__uncultured_bacterium |
| 2b0b2e42a4ec8f4c2650f0c79e527b69 | 7 | 0 | 0 | 0 | 0 | 0 |  |
| 10f9adc57789aaa9c417eb054cb3a5ee | 7 | 0 | 0 | 0 | 0 | 0 | s__uncultured_bacterium |
| 47bf9fd2dfa660cd84af1d014cc15513 | 0 | 0 | 0 | 0 | 7 | 0 |  |
| 702a3b9afea20104a03a7dc12b12a682 | 0 | 0 | 0 | 0 | 7 | 0 | s__uncultured_bacterium |
| cc88b71dec580d84105ee4cbbb8ddb87 | 0 | 0 | 0 | 0 | 7 | 0 | s__uncultured_Xiphinematobacteriaceae |
| fc47c24cdb970246c8f84157b847804f | 0 | 0 | 0 | 0 | 7 | 0 | s__uncultured_soil |
| fed1166cf97fbab8e2047a0fa96368be | 0 | 7 | 0 | 0 | 0 | 0 |  |
| 4ea0b093afe2815a4ecce54966ef93e8 | 0 | 7 | 0 | 0 | 0 | 0 | s__[Clostridium]_ultunense |
| 4ec75405869c1ff38b7b084000e552c3 | 0 | 7 | 0 | 0 | 0 | 0 | s__metagenome |
| 2f3217712516e4319523f65812f6b61f | 0 | 7 | 0 | 0 | 0 | 0 | s__Parachlamydia_sp. |
| 8ff03db8dac75f0e66da91be47c89b27 | 0 | 0 | 6 | 0 | 0 | 0 | s__uncultured_planctomycete |
| 5c4549d0f511199b40a0a1cd0886dc7b | 0 | 0 | 6 | 0 | 0 | 0 | s__uncultured_bacterium |
| dab2d95018b2ea62d6cded58921f6f47 | 0 | 0 | 0 | 0 | 6 | 0 | s__uncultured_bacterium |
| d6b4b23177eca05fcc2f65105112384d | 0 | 0 | 0 | 0 | 6 | 0 |  |
| 0f66dcceac4de370aee94ca38431ea68 | 0 | 0 | 0 | 6 | 0 | 0 | s__uncultured_bacterium |
| bfddf89109fe3bbe62e49ea81fe91560 | 0 | 6 | 0 | 0 | 0 | 0 |  |
| 67f5deb3163fb95e932a6e74c0fb8d41 | 0 | 6 | 0 | 0 | 0 | 0 | s__uncultured_planctomycete |
| 68531a07cc38889cc93403b232d3469c | 0 | 0 | 0 | 0 | 0 | 5 | s__Cyanobacteria/Melainabacteria_group |
| 79ebeef961207acca29ea7dafbc535f2 | 0 | 0 | 0 | 0 | 0 | 5 |  |
| 9ad3e41ba2ebb60837a243236fe8faf4 | 0 | 0 | 5 | 0 | 0 | 0 |  |
| 95d8a0e59c8940dde7841a76a39402d9 | 0 | 0 | 5 | 0 | 0 | 0 |  |
| fe5262d5c4711a6d4cace2b0c98bab9b | 0 | 0 | 5 | 0 | 0 | 0 | s__wastewater_metagenome |
| 39ba97d6338f97869200054596804f7e | 0 | 0 | 5 | 0 | 0 | 0 | s__uncultured_Bacteroidetes |
| 4330c7a0d941d28ac5f82f0dfa31f52b | 5 | 0 | 0 | 0 | 0 | 0 | s__metagenome |
| 93944d8e9acf05268a7b7e20cfb555a9 | 0 | 0 | 0 | 5 | 0 | 0 |  |
| 87619634a625cb8dbc1684289676962a | 0 | 0 | 0 | 5 | 0 | 0 |  |
| 612e69b616dab3b98fcd21f1d2b41a83 | 0 | 5 | 0 | 0 | 0 | 0 | s__Empedobacter_sp. |
| fd6d3392205c4d9a56ca2dd24933f90a | 0 | 5 | 0 | 0 | 0 | 0 | s__uncultured_Planctomycetales |
| 96ff135116e07eb091d07cea82f31bf4 | 0 | 5 | 0 | 0 | 0 | 0 | s__uncultured_bacterium |
| 567da236a3800f5350b444fb178c4442 | 0 | 5 | 0 | 0 | 0 | 0 |  |
| 8cdbf6f30a70f7f37a21152729f9e5ca | 0 | 5 | 0 | 0 | 0 | 0 | s__uncultured_Sphingobacterium |
| dad60521f5a6e8cbbf9c5c2255c3df00 | 0 | 5 | 0 | 0 | 0 | 0 |  |
| 34133feebe9627bf24166a547d04abd4 | 0 | 0 | 4 | 0 | 0 | 0 |  |
| 8876ec14dfb694f3420985d7dedc4b03 | 0 | 0 | 4 | 0 | 0 | 0 |  |
| 87d0c44ef4943b558c5bb48fe27add4c | 0 | 0 | 4 | 0 | 0 | 0 |  |
| 21774ca9644fd82ac77f12ba0dab4c86 | 0 | 0 | 4 | 0 | 0 | 0 | s__metagenome |
| 5f8752377227132dafc04c86d7d9725c | 0 | 0 | 4 | 0 | 0 | 0 | s__metagenome |
| 7d3aa99236b0f876b4efa547e1bbe324 | 0 | 0 | 4 | 0 | 0 | 0 | s__uncultured_Chloroflexi |
| 9cc0e4b57b072329dc79a54433120c07 | 4 | 0 | 0 | 0 | 0 | 0 | s__uncultured_Actinobacteridae |
| 99c62ce1b1cf949b5939e6e303ab78d3 | 0 | 0 | 0 | 0 | 4 | 0 |  |
| 40fa4d4fd69978e3b575d879736f841c | 0 | 0 | 0 | 4 | 0 | 0 | s__uncultured_bacterium |
| 8a1768a584146e027df8593de79f05d5 | 0 | 0 | 0 | 0 | 0 | 3 |  |
| 6ab0f56b6a3669425229eedea73e5f02 | 0 | 0 | 0 | 0 | 0 | 3 |  |
| 43498ab84846397cd6d44ac401f9e3e3 | 0 | 0 | 3 | 0 | 0 | 0 |  |
| 7907fafda2631a812561872bb193a433 | 0 | 0 | 3 | 0 | 0 | 0 | s__uncultured_forest |
| a3bc7f9c94bb6adc06450a7bfe3f1842 | 0 | 0 | 3 | 0 | 0 | 0 | s__metagenome |
| 14510bc8b82ee4c5280e53b83f4b2619 | 0 | 0 | 3 | 0 | 0 | 0 | s__uncultured_bacterium |
| f6bd789101c9a3aa7b4b4ea96510653b | 0 | 0 | 3 | 0 | 0 | 0 |  |
| 4380def8d7b31f96dd6d4f97ee10a2aa | 0 | 0 | 3 | 0 | 0 | 0 |  |
| a3ef7b542734d0bbade2073bc4c4badf | 0 | 0 | 3 | 0 | 0 | 0 |  |
| b72e6309f0689f7d2d3d72858ba3b01d | 0 | 0 | 3 | 0 | 0 | 0 |  |
| bf2e0e9ab63e126b1eb71bfab1777bb6 | 0 | 0 | 3 | 0 | 0 | 0 |  |
| 1da9f0abbd425d52be212b975d7887e6 | 3 | 0 | 0 | 0 | 0 | 0 | s__Rubrobacterineae_bacterium |
| 9f9d51945dacd79960b3eb2568b9b80d | 3 | 0 | 0 | 0 | 0 | 0 | s__uncultured_bacterium |
| 414254f3f1999b60659bb1db759fe34d | 3 | 0 | 0 | 0 | 0 | 0 |  |
| fe951b6dbc8c34622d2b89320ba15288 | 0 | 0 | 0 | 0 | 3 | 0 |  |
| 6b835e9b50885289dafb6e9b1477fb37 | 0 | 0 | 0 | 0 | 3 | 0 | s__uncultured_bacterium |
| 12f515dac195f0264d6a386d5ae2e6ea | 0 | 0 | 0 | 0 | 3 | 0 | s__uncultured_bacterium |
| 46c8b1a17a10015095f9cae4c959bf4d | 0 | 0 | 0 | 3 | 0 | 0 | s__Hyphomicrobium_sp. |
| b8fef76d73489eecb09395abe5aa55fe | 0 | 0 | 0 | 3 | 0 | 0 | s__Algoriphagus_resistens |
| 719bf33bbce046eea2df90846f314d29 | 0 | 0 | 0 | 3 | 0 | 0 |  |
| d34192dfb18c15d2b41ef6c580a85671 | 0 | 3 | 0 | 0 | 0 | 0 |  |
| eb0dbb229496489c81d8b5b90d5c6d99 | 0 | 3 | 0 | 0 | 0 | 0 |  |
| 94ea4c9cfcc094dd45f9d0248badce87 | 0 | 3 | 0 | 0 | 0 | 0 | s__uncultured_bacterium |
| 46c2cba4e1a6677c2be655f309da4852 | 0 | 3 | 0 | 0 | 0 | 0 |  |
| f8348381a5553235e7b219d9b1b3f517 | 0 | 0 | 0 | 0 | 0 | 2 |  |
| e2c8d37f2bfb99d924334f64c2872d23 | 0 | 0 | 0 | 0 | 0 | 2 |  |
| 777c24947fab6565dabf4b18cdb98cc8 | 0 | 0 | 0 | 0 | 0 | 2 |  |
| 9ec1c214fc430953b5e8945ffa39bf79 | 0 | 0 | 2 | 0 | 0 | 0 |  |
| b0a2b7286898e8ef9e2b895628fd3cbd | 0 | 0 | 2 | 0 | 0 | 0 |  |
| 49b5e64a02cc2763595c13c52f8a9d45 | 0 | 0 | 2 | 0 | 0 | 0 | s__Algoriphagus_terrigena |
| 68e9443f944fee0086ba9a50b38949c4 | 0 | 0 | 2 | 0 | 0 | 0 |  |
| 2d59e96c717565de83bd90b1ca6839a9 | 0 | 0 | 2 | 0 | 0 | 0 |  |
| 30af83150a37978c11d60b44f423b056 | 0 | 0 | 2 | 0 | 0 | 0 |  |
| 5d2290f81534f73c1ed8bd7a19a51c66 | 0 | 0 | 2 | 0 | 0 | 0 |  |
| bb8663ef84833d0d80746074ccbdc931 | 0 | 0 | 2 | 0 | 0 | 0 | s__Tissierella_sp. |
| 64bf264dbe83f97c600c9266327de810 | 0 | 0 | 2 | 0 | 0 | 0 |  |
| c3416fe092f0e5f22997c7fef9d06e24 | 0 | 0 | 2 | 0 | 0 | 0 | s__uncultured_Desulfovibrionales |
| 9160962eca4aa7a5f65d3f2a1ed86949 | 2 | 0 | 0 | 0 | 0 | 0 |  |
| 63e5b2ab6d2d5dd8e92a1af1b4a7f706 | 2 | 0 | 0 | 0 | 0 | 0 |  |
| e29da22e468957cc393f1b07b7c9aab9 | 2 | 0 | 0 | 0 | 0 | 0 |  |
| 21ca0b30f130fa1c5443d4b434a8957f | 2 | 0 | 0 | 0 | 0 | 0 | s__uncultured_Verrucomicrobia |
| 6bc1e7ddf54bc60e4395925871a331bf | 2 | 0 | 0 | 0 | 0 | 0 | s__uncultured_organism |
| 258806159dd3521efffb23072cea52d8 | 2 | 0 | 0 | 0 | 0 | 0 |  |
| 2a9fb08e2531ad45c1f4f66dc8a23263 | 2 | 0 | 0 | 0 | 0 | 0 |  |
| 59f945de75375025da74c3c5e8819791 | 2 | 0 | 0 | 0 | 0 | 0 | s__Steroidobacter_sp. |
| 80035208b13cdcaa445e4abf32bed7b7 | 2 | 0 | 0 | 0 | 0 | 0 |  |
| 12aba1ff53af96277d29c4e031603062 | 2 | 0 | 0 | 0 | 0 | 0 | s__uncultured_beta |
| 8800f227bab6aa89fa7dacb8223947a0 | 0 | 0 | 0 | 0 | 2 | 0 |  |
| da63114c508350893c0f47a97d6d0e3d | 0 | 0 | 0 | 2 | 0 | 0 |  |
| f9f843cb5f01463aacdf1c7b4e980e05 | 0 | 0 | 0 | 2 | 0 | 0 | s__uncultured_bacterium |
| 4ade451ffe8ebd722b5c9b77ff6535bf | 0 | 0 | 0 | 2 | 0 | 0 |  |
| e0c711f56cf9422fb0e387c3d1904c3a | 0 | 0 | 0 | 2 | 0 | 0 |  |
| ad886de3f8ee8c53e5d085d8374c163c | 0 | 2 | 0 | 0 | 0 | 0 |  |
| f40e228976975a135947d3c88b377199 | 0 | 2 | 0 | 0 | 0 | 0 | s__uncultured_bacterium |
| fe6d81d1e4af5a71071b8ef0d2b6cc51 | 0 | 2 | 0 | 0 | 0 | 0 |  |
| 9cf5f2b72a044b35e78fde181b131eb1 | 0 | 2 | 0 | 0 | 0 | 0 |  |
| 905ab1f69f45fded8fde72c1365f63ba | 0 | 2 | 0 | 0 | 0 | 0 | s__uncultured_Flexibacter |
| 03108aaaeca88690873c16a27d3e3c8c | 0 | 2 | 0 | 0 | 0 | 0 | s__uncultured_bacterium |
| 71b5d35741d3a25bb817bdb01fb7a1f6 | 0 | 0 | 0 | 1 | 0 | 0 |  |
| 941665f340a19543a69a2a3340036bec | 0 | 1 | 0 | 0 | 0 | 0 |  |
